# Postcranial skeletal anatomy of the holotype and referred specimens of *Buitreraptor gonzalezorum* Makovicky, Apesteguía and Agnolín 2005 (Theropoda, Dromaeosauridae), from the Late Cretaceous of Patagonia

**DOI:** 10.7717/peerj.4558

**Published:** 2018-03-26

**Authors:** Federico A. Gianechini, Peter J. Makovicky, Sebastián Apesteguía, Ignacio Cerda

**Affiliations:** 1 Instituto Multidisciplinario de Investigaciones Biológicas (IMIBIO-SL), CONICET—Universidad Nacional de San Luis, San Luis, Argentina; 2 Section of Earth Sciences, Integrative Research Center, Field Museum of Natural History, Chicago, IL, USA; 3 CONICET, Fundación de Historia Natural ‘Félix de Azara’, CEBBAD, Universidad Maimónides, Buenos Aires, Argentina; 4 CONICET, Instituto de Investigación en Paleobiología y Geología, Universidad Nacional de Río Negro, General Roca, Río Negro, Argentina

**Keywords:** Patagonia, Late Cretaceous, Paraves, Dromaeosauridae, Unenlagiinae, Osteology, Paleohistology, *Buitreraptor gonzalezorum*

## Abstract

Here we provide a detailed description of the postcranial skeleton of the holotype and referred specimens of *Buitreraptor gonzalezorum*. This taxon was recovered as an unenlagiine dromaeosaurid in several recent phylogenetic studies and is the best represented Gondwanan dromaeosaurid discovered to date. It was preliminarily described in a brief article, but a detailed account of its osteology is emerging in recent works. The holotype is the most complete specimen yet found, so an exhaustive description of it provides much valuable anatomical information. The holotype and referred specimens preserve the axial skeleton, pectoral and pelvic girdles, and both fore- and hindlimbs. Diagnostic postcranial characters of this taxon include: anterior cervical centra exceeding the posterior limit of neural arch; eighth and ninth cervical vertebral centra with lateroventral tubercles; pneumatic foramina only in anteriormost dorsals; middle and posterior caudal centra with a complex of shallow ridges on lateral surfaces; pneumatic furcula with two pneumatic foramina on the ventral surface; scapular blade transversely expanded at mid-length; well-projected flexor process on distal end of the humerus; dorsal rim of the ilium laterally everted; and concave dorsal rim of the postacetabular iliac blade. A paleohistological study of limb bones shows that the holotype represents an earlier ontogenetic stage than one of the referred specimens (MPCA 238), which correlates with the fusion of the last sacral vertebra to the rest of the sacrum in MPCA 238. A revised phylogenetic analysis recovered *Buitreraptor* as an unenlagiine dromaeosaurid, in agreement with previous works. The phylogenetic implications of the unenlagiine synapomorphies and other characters, such as the specialized pedal digit II and the distal ginglymus on metatarsal II, are discussed within the evolutionary framework of Paraves.

## Introduction

*Buitreraptor gonzalezorum* is a paravian theropod whose remains were found in Late Cretaceous outcrops of the Candeleros Formation in the “La Buitrera” fossiliferous area, in Patagonia, Argentina. The paleontological significance of the many transformative discoveries made at this site was already detailed in our previous papers (Apesteguía & Zaher, 2006; Gianechini, Makovicky & Apesteguía, 2011; Rougier, Apesteguía & Gaetano, 2011; Makovicky, Apesteguía & Gianechini, 2012; Gianechini & de Valais, 2016).

Most phylogenetic analyses performed on coelurosaurian theropods recover *Buitreraptor* as member of a monophyletic Dromaeosauridae, within the subfamily Unenlagiinae, although some recent analyses recover *Buitreraptor* and other unenlagiines as stem avialans (Agnolín & Novas, 2011, 2013). Dromaeosauridae has experienced a remarkable increase in diversity since the 2000s, including species with both small and large body sizes, some of them represented by almost complete skeletons preserving plumage (Currie & Varricchio, 2004; Norell et al., 2006; Turner, Hwang & Norell, 2007; Turner et al., 2007; Longrich & Currie, 2009; Xu et al., 2010; Zheng et al., 2010; Evans, Larson & Currie, 2013; Han et al., 2014; DePalma et al., 2015; Lü & Brusatte, 2015). Unfortunately, the Gondwanan record of these theropods remains sparse, and until now, the most significant Gondwanan specimens were found in Patagonia and Madagascar. The Patagonian record of unenlagiines currently includes five taxa, three of which preserve only postcranial remains: *Unenlagia comahuensis*, *Unenlagia paynemili* and *Neuquenraptor argentinus* and two species with cranial remains, i.e., *B. gonzalezorum* and *Austroraptor cabazai*. Recently, a fragmentary coelurosaur represented only by hindlimb remains, but with possible deinonychosaurian affinities was described as *Pamparaptor micros* (Porfiri, Calvo & Dos Santos, 2011). Its potential relationships with unenlagiines have not yet been thoroughly evaluated.

*Buitreraptor* is the best represented unenlagiine to date. Its holotype consists of an almost complete, semi-articulated, and very well-preserved skeleton and at least five referred specimens are known. This taxon was named by Makovicky, Apesteguía & Agnolín (2005), who concluded that it is the earliest dromaeosaurid found so far in Gondwana. The anatomical information provided by this taxon was significant for uniting Gondwanan dromaeosaurids within their own monophyletic clade, and for the understanding of the character distributions and morphological trends in paravian phylogeny. However, some traits of *Buitreraptor*, and also of other unenlagiines, are similar to the anatomy of basal avialans, lending support to the alternate phylogenetic hypothesis proposed by Agnolín & Novas (2011, 2013) mentioned above.

*Buitreraptor* was only briefly described by Makovicky, Apesteguía & Agnolín (2005), and a detailed osteology has been wanting. We recently provided a comperehensive description of the cranial anatomy of this taxon (Gianechini, Makovicky & Apesteguía, 2017), and here we offer a detailed description of the postcranial skeleton. Details on individual parts of the skeleton of *Buitreraptor* have been published elsewhere (Gianechini & Apesteguía, 2011; Agnolín & Novas, 2013; Novas et al., 2018), but an integrated and complete description of the anatomy of this theropod is required to allow comparative studies with other coelurosaurs. Recently, a new specimen was prepared (MPCN-PV-598; Agnolín & Novas, 2013; Novas et al., 2018), which preserves much of the postcranial skeleton and reveals the anatomy of parts not present in the holotype, such as an articulated manus and pes. The number and preservational quality of the specimens, including the holotype, MPCN-PV-598 and other referred material, make *Buitreraptor* the best represented non-avian coelurosaur from Gondwana to date.

## Systematic Paleontology

Theropoda Marsh, 1881
Maniraptora Gauthier, 1986
Deinonychosauria Colbert & Russell, 1969
Dromaeosauridae Matthew & Brown, 1922
Unenlagiinae Bonaparte, 1999
*B. gonzalezorum* Makovicky, Apesteguía & Agnolín, 2005

Holotype—MPCA 245, almost complete and semi articulated skeleton, including the cranium and the postcranium. The postcranium includes incomplete axis and eight cervical vertebrae from the anterior, middle and posterior sections of the neck, some bearing cervical ribs; 15 dorsal vertebrae; incomplete sacrum which includes five fused sacral vertebrae; 15 caudal vertebrae from the anterior, middle and distal zones of the tail, some with chevrons; middle to posterior isolated chevrons; seven dorsal ribs, one of them almost complete; left and right scapula and coracoid; furcula; right humerus and proximal half of the left humerus; right radius and ulna; an incomplete metacarpal and some phalanges of the hand; both ilia, the left one in contact with the sacrum; right ischium; both femora; right tibia and fibula; proximal fragments of the left fibula and tibia; metatarsals; several pedal phalanges; and several indeterminate fragments.

Referred specimens—MPCA 238, corresponding to a second individual, preserves three fused sacral vertebrae; the first two caudal vertebrae in articulation with the sacrum, and bearing the first chevron in articulation between them; right ilium and pubis; right femur; right tibia with fused astragalus and calcaneum; metatarsals I–IV; possible pedal phalanx I-1 and phalanges II-1 and II-2; MPCA 238 also includes a cast of the ungual phalanx of the second digit made from a natural mold preserved in the rock.

MPCA 478, comprises the distal portion of a right metatarsal II, along with its articulated pedal phalanges II-1, II-2 and II-3. This specimen also includes a possible distal articular portion of metatarsal III articulated with the proximal portion of the first phalanx, and a distal portion of a phalanx from digit III or IV articulated with the proximal part of the following phalanx.

MPCA 471, consists of two isolated phalanges possibly from the hand (MPCA 471-A); an indeterminate fragment (MPCA 471-B); several fragments of manual phalanges and possible metacarpals (MPCA 471-C); and a right fragmentary metatarsus, including parts of the articulated matatarsals II, III and IV; an isolated pedal ungual phalanx and an unknown fragment (MPCA 471-D).

MPCN-PV-598, was described (Novas et al., 2018). It preserves cervical, dorsal, sacral and caudal vertebrae, partial pectoral and pelvic girdles, and bones from the forelimbs and hindlimbs, including a nearly complete and articulated hand and foot. Comparisons are made to this specimen where relevant, but its anatomy it is not described here as it was covered extensively by Novas et al. (2018).

## Horizon and Locality

The holotype and the referred specimens of *B. gonzalezorum* come from the fossiliferous area of “La Buitrera,” located in the northwestern part of Río Negro Province, Argentina, between the towns of Villa El Chocón and Cerro Policía, 80 km SW of Cipolletti, and close to the southern coast of Lake Ezequiel Ramos Mexía. The materials were collected from reddish, massive sandstones of the Candeleros Formation (Cenomanian). Although some parts of this formation were deposited by braided fluvial systems (Garrido, 2010), most research supports a major aeolian component to the La Buitrera facies (Spalletti & Gazzera, 1989), interpreting these as ancient dune fields and playa-lake environments. Candia Halupczok et al. (2016a, 2016b, 2018) recognized most of the La Buitrera area as part of a paleodesert formed east of a craton border in the Neuquén Basin, and recently dubbed the Kokorkom Desert (Apesteguía et al., 2016) with an areal extent of around 826 km^2^. The almost horizontal sandstones at La Buitrera form the uppermost 50 m of the Candeleros Formation outcrops there. These sandstones are aeolian in origin and are interpreted as a wet dunefield with evidence for small ephemeral lakes and playa environments between intensely deformed mass wasted sand deposits representing collapsed dunes. This is a common phenomenon in dune fields that are contracting or in areas where phreatic changes during aquifer loading generate high instability and dune collapse/mass wasting episodes. The paleodesert in the central to eastern parts of the basin suggest an arid center for West Gondwana during the Cretaceous greenhouse period, which developed during underfed basin stages (Candia Halupczok et al., 2016a, 2016b, 2018). The age of the Candeleros Formation is not well constrained, but Garrido (2010) sugested a lower Cenomanian age. Its deposition is estimated to have begun about 100 Ma ago (Leanza et al., 2004) whereas deposition of the overlying Huincul Formation was initiated close to 90 Ma ago (Corbella et al., 2004). The age of the Candeleros Formation outcrops at La Buitrera is estimated to be around 95 to 92 Ma (Apesteguía, 2008).

## Revised Diagnosis (Based Only on Postcranial Characters)

Paravian theropod which differs from other non-avian theropods in the following unique combination of postcranial characters (autapomorphies marked with an asterisk): anterior cervical centra extend beyond the posterior limit of neural arch (shared with some other coelurosaurs and with avialans); eighth and ninth cervical vertebrae with ridges on the lateroventral surfaces of the centra terminating as small tubercles posteriorly*; pneumatic foramina present only on the first and second dorsal vertebral centra (*Rahonavis* possibly has a pneumatic opening in the first or second dorsal centrum, whereas *Austroraptor* and *Unenlagia* exhibit well-developed pleurocoels along all the dorsal series); tubercles on the ventral surface of last sacral centrum (possibly shared with some Liaoning paravians such as *Sinornithosaurus*); middle and posterior caudal vertebrae with a complex of ridges on lateral surfaces of centra (shared with *Rahonavis*); pneumatic furcula with two pneumatic foramina on the ventral surface (possible pneumatic foramina also observed in *Bambiraptor*); scapular blade transversely expanded at mid-length* (Novas et al., 2018); well-projected flexor process on the distal margin of the humerus (shared with *Rahonavis* and some avialans); extremely slender manual elements, hand longer than the femur (117% of total femoral length; Novas et al., 2018); dorsal rim of the iliac blade laterally everted extending beyond acetabular rim* (other paravians have a less everted dorsal border); expanded and lobed brevis shelf, projected laterally from the posterior end of the ilium (shared with other unenlagiines); concave dorsal rim of the postacetabular iliac blade (shared with other unenlagiines); subarctometatarsal metatarsus, with projecting flange on the posterolateral rim of metatarsal IV (shared with other unenlagiines, microraptorines and basal troodontids); pedal phalanx II-2 with asymmetrical medial proximoventral process (shared with other unenlagiines); ungual phalanx of pedal digit II markedly developed with respect to the other pedal unguals (shared with dromaeosaurids and troodontids).

## Description and Comparisons

### Axial skeleton

#### Cervical vertebrae

**Figure 1:**
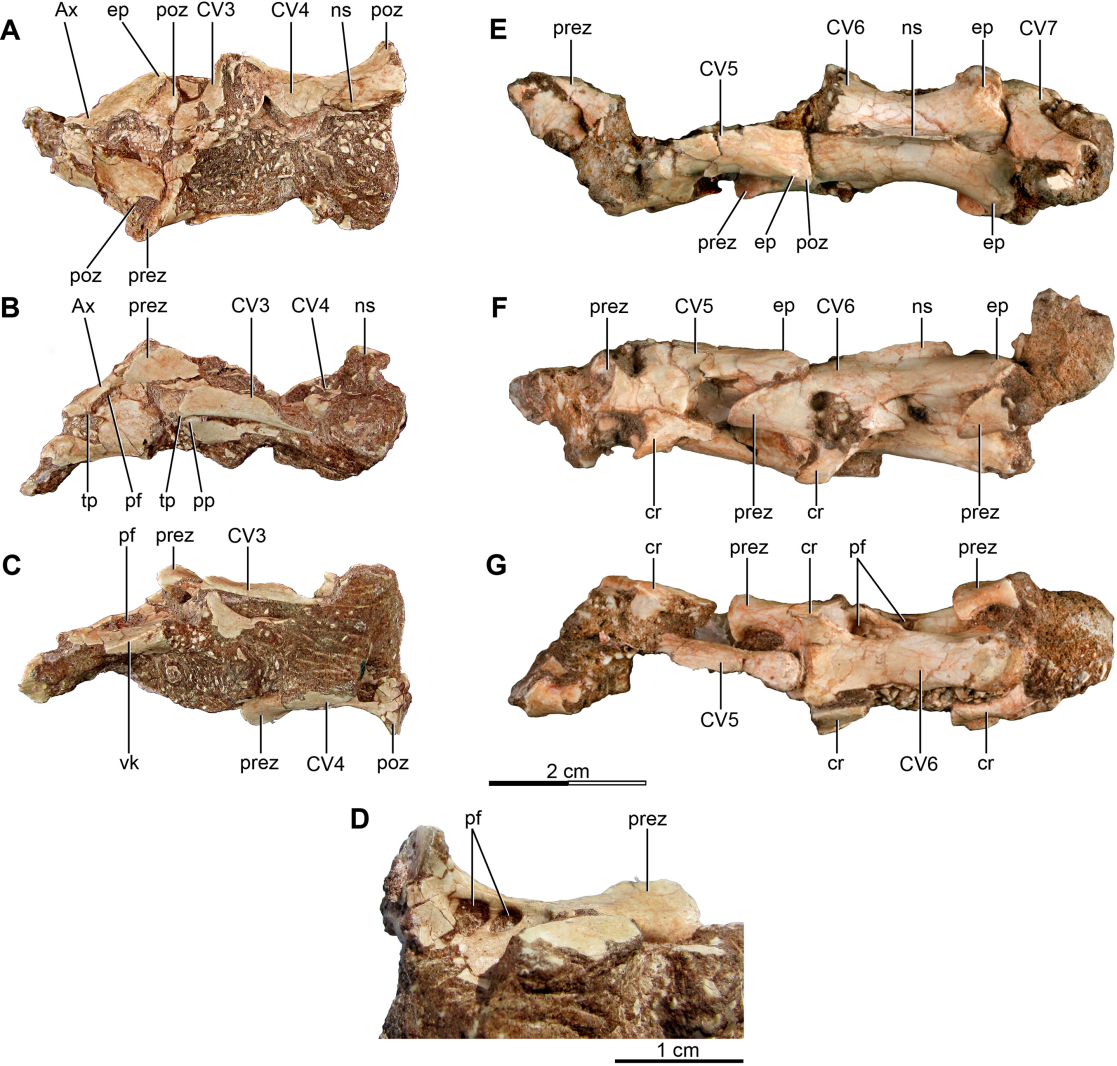
Anterior and mid cervical vertebrae of the holotype of *Buitreraptor gonzalezorum* (MPCA 245). (A–C) Axis and third and fourth cervical vertebrae, in (A) dorsal, (B) left lateral and (C) ventral view. (D) Ventral view of the neural arch of the fourth vertebra, showing pneumatic foramina. (E–G) Fifth to seventh cervical vertebrae, in (E) dorsal, (F) left lateral and (G) ventral view. Scales: 2 cm for A–C and E–G, 1 cm for D. Ax, axis; cr, cervical rib; CV, cervical vertebra; ep, epipophysis; ns, neural spine; pf, pneumatic foramen; poz, postzygapophysis; pp, parapophysis; prez, prezygapophysis; tp, transverse process; vk, ventral keel.

**Figure 2:**
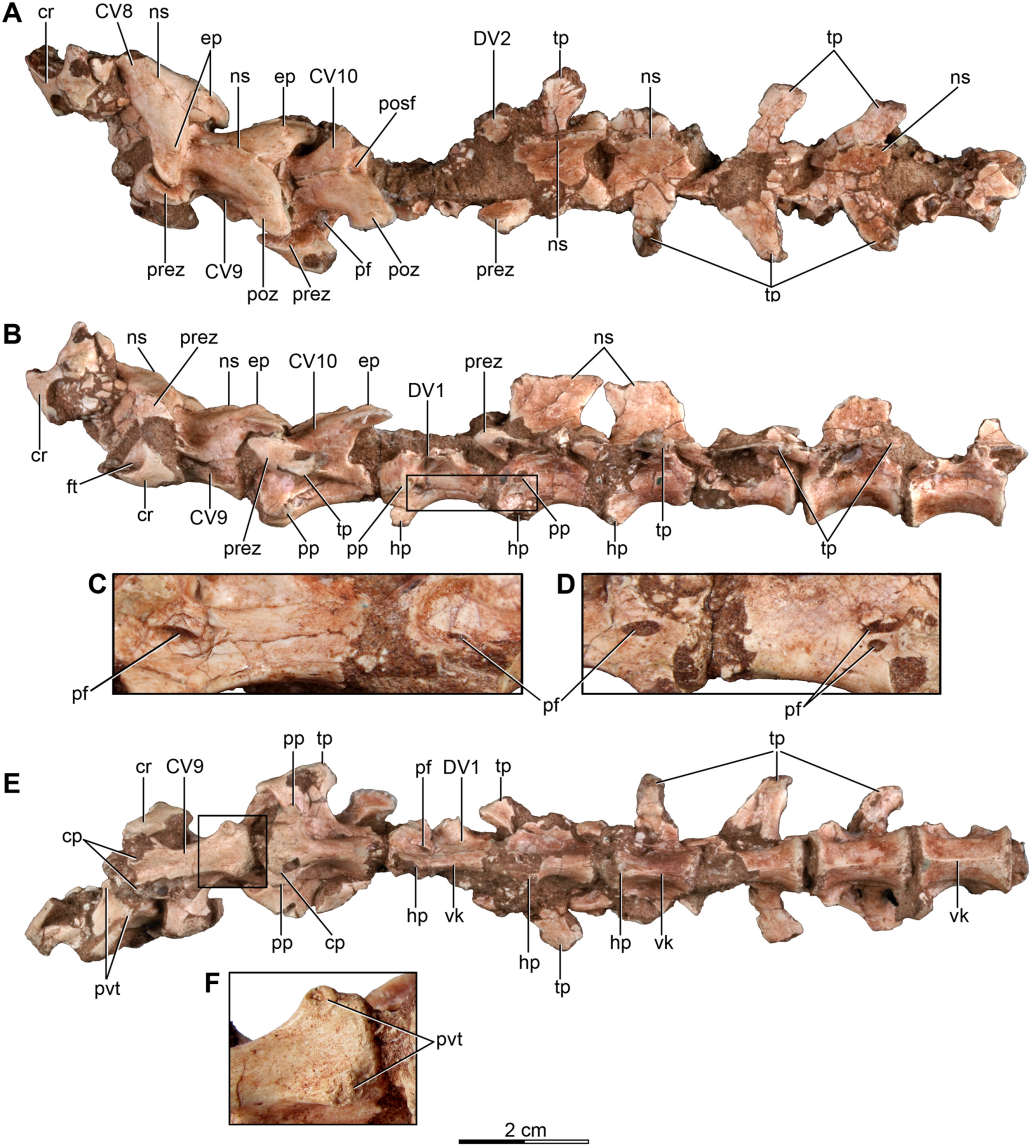
Posterior cervical and anterior dorsal vertebrae of the holotype of *Buitreraptor gonzalezorum* (MPCA 245). (A) Dorsal, (B) left lateral and (E) ventral view. (C) Detail of the box inset in B, showing pneumatic foramina on the first and second dorsal centra. (D) Detail of the centra of the last cervical and first dorsal vertebrae in right lateral view, showing pneumatic foramina. (F) Detail of the box inset in E, showing the posteroventral tubercles on the ninth cervical centrum. Scale: 2 cm for all elements, except for C, D and F. cp, carotid process; cr, cervical rib; CV, cervical vertebra; DV, dorsal vertebra; ep, epipophysis; ft, foramen transversarium; hp, hypapophysis; ns, neural spine; pf, pneumatic foramen; posf, post-spinal fossa; poz, postzygapophysis; pp, parapophysis; prez, prezygapophysis; pvt, posteroventral tubercle; tp, transverse process; vk, ventral keel.

*Buitreraptor* is the only unenlagiine that preserves a nearly complete cervical series, lacking only the atlas. In the holotype, the cervical vertebrae are preserved in three sections. Assuming ten cervical vertebrae were present as in other coelurosaurs, we identify the rostralmost section to comprise part of the axis and cervicals 3–4 (Figs. 1A–1D). The second section includes parts of cervicals 5–7 (Figs. 1E–1G), and cervicals 8–10 are in articulation with the first dorsal (Fig. 2).

##### Axis

The axis is fragmentary and only preserves a portion of the centrum and the posterior part of the neural arch bearing the postzygapophyses (Figs. 1A–1C). Neither the dens nor the axis intercentrum are preserved. The centrum is markedly transversely compressed, as in *Deinonychus* (Ostrom, 1969), and the ventral surface is narrow but rounded. A small knob is observed on the left lateral surface near the preserved rostral end, and is slightly raised and close to the ventral margin. It may correspond to the left parapophysis. The lateral zone of the centrum posterior to the presumptive parapophysis is not preserved and therefore the presence of a pleurocoel cannot be confirmed. Pneumatic openings are present in the axis centrum of dromaeosaurids as *Deinonychus* and *Mahakala* (Ostrom, 1969; Turner, Pol & Norell, 2011).

The neural arch is transversely expanded and posteriorly overhangs the centrum. The diapophysis is located on the anterolateral part of the arch, close to the lateral surface of the centrum and posterodorsal to the likely parapophysis. It is a small protuberance that ends in a sharp, laterally projected edge. A lamina extends between the diapophysis and the postzygapophysis. Ventral to this lamina there is an opening that resembles a pneumatic foramen.

The neural arch swells posterodorsally in lateral view. In dorsal view, it is constricted in the middle zone but expands posteriorly to an approximately triangular shape. The postzygapopyses together with their epipophyses have an inflated appearance. The posterodorsal inclination of the neural arch is observed in other dromaeosaurids as *Deinonychus* and *Tsaagan* (Ostrom, 1969; Norell et al., 2006), but is common in the theropod axis. The postzygapophyses are distinctively extended posterolaterally beyond the posterior border of the centrum, and bear epipophyses on their dorsal surfaces (Fig. 1A). These epipophyses are broad and blunt in dorsal view and their ends do not project past the posterior margin of the postzygapophyses as in *Mahakala* and other coelurosaurs such as *Microvenator* (Makovicky & Sues, 1998; Turner, Pol & Norell, 2011), but in contrast to the more prominent epipophyses of other dromaeosaurids such as *Deinonychus* (Ostrom, 1969). A sheet of bone connects the opposing postzygapophyses as in other coelurosaurs including *Deinonychus*, *Tsaagan* and *Ornithomimus* (Ostrom, 1969; Makovicky, Kobayashi & Currie, 2004; Norell et al., 2006). The neural spine is broken but its base is located toward the posterior zone of the arch.

##### Postaxial cervical vertebrae

The third cervical vertebra only has a small portion of the centrum and the anterior part of the neural arch including both prezygapophyses still in articulation with the axis (Figs. 1A–1C). The left prezygapophysis is the better preserved of the two and extends anterolaterally beyond the anterior end of the centrum as well as beyond the lateral margin of the neural arch in dorsal view. The diapophysis forms a ventrally directed point along the lateral margin of the neural arch about midway between the pre- and postzygapophyses, and is thus similar in morphology and location to those observed in *Deinonychus*, *Tsaagan* and *Austroraptor* (Ostrom, 1969; Norell et al., 2006; Novas et al., 2009). A postzygodiapophyseal lamina extends from the diapophysis and forms the lateral border of an infrapostzygapophyseal fossa that is filled with matrix. The neural arch is incompletely preserved but is about as long as it is wide. It shows evidence of a constriction at midlength and is strongly lateroventrally inclined, as is also observed in *Tsaagan* and *Byronosaurus* (Makovicky et al., 2003; Norell, Makovicky & Clark, 2000; Norell et al., 2006).

The fourth cervical preserves only the right half of the neural arch and part of the centrum (Figs. 1A–1D). The lateral sides of the arch are markedly ventrally expanded, overhanging the centrum. As in the third cervical, the preserved right prezygapophysis overhangs the centrum anteriorly and laterally. The small diapophysis is ventrally and slightly laterally inclined. It remains in articulations with the cervical rib, which is platelike rather than rodlike in appearance. The right postzygapophysis is posterolaterally projected and bears a weakly developed epipophysis, visible only as a small dorsal protrusion that it does not overhang the posterior end of the postzygapophysis. The neural spine is broken. A fossa is observed posteriorly to the diapophysis and ventrally oriented, wherein two openings are located and separated by a very thin bar. These represent the infradiapophyseal and infrapostzygapophyseal pneumatic fossae (Fig. 1D).

The cervicals of the middle zone of the neck are best represented by the sixth one, which is far better preserved than the adjacent elements (Figs. 1E–1G). This vertebra is markedly anteroposteriorly elongated, when compared to more anterior and posterior cervical vertebrae (Table S1). The vertebrae from the middle zone of the neck (cervicals 5–7) are distinctly the longest elements in the neck, as also occurs in other paravians such as *Microraptor*, *Mei*, *Anchiornis* and *Archaeopteryx* (Xu & Norell, 2004; Wellnhofer, 1974; Pei et al., 2014, 2017), although this is a trait not observed in *Austroraptor* (Novas et al., 2009). In some dromaeosaurids including *Deinonychus*, *Sinornithosaurus* and *Bambiraptor* the posterior cervicals are shorter than the middle ones (Ostrom, 1969; Xu, 2002; Burnham, 2004), a feature that also can be observed in ornithomimosaurs (Osmólska, Roniewicz & Barsbold, 1972; Kobayashi & Lü, 2003), alvarezsauroids (Perle et al., 1994; Chiappe, Norell & Clark, 2002) and avialans (Wellnhofer, 1974; Chiappe & Walker, 2002). The preserved cervicals of *Mahakala*, which are considered from the middle section of the neck, have elongate neural arches (Turner, Pol & Norell, 2011).

The sixth cervical has an anteriorly low centrum, that increases in depth posteriorly, as appears to have been the case in the fifth cervical, a feature related to the S-shaped curvature of the neck. The anterior face of the centrum is anteroventrally inclined whereas the posterior one is vertical. Because the vertebrae are articulated to each other the articular faces are partly covered, but in the specimen MPCN-PV-598 the cervicals have been interpreted as heterocoelous by Novas et al. (2018). However, as seen in Fig. 1G, the anterior intercentral articulation is not dorsoventrally compressed, but rather strongly angled, and thus is not fully homologous with the heterocoleus vertebrae of pygostylian birds. The ventral surface has a very faint keel, which runs longitudiunally along the midsection of the centrum but does not reach either the anterior and posterior ends of the centrum, which are gently concave in ventral view.

A cervical rib is fused to this vertebra delimiting a foramen transversarium (*sensu* Baumel & Witmer, 1993). The cervical rib is incomplete but has a pointed rostral process and a horizontal lateral ridge. A large opening that has punctured the area where the rib would meet the diapophysis likely represents a scavenging arthropod trace as indicated by the numerous bone fragments within the opening. Two laminae extend posteriorly from the diapophyseal region, one of which extends obliquely ventrally toward the posterolateral face of the centrum whereas the other forms the postzygodiapophyseal lamina that defines the lateral edge of the small infrapostzygapophyseal fossa. A pneumatic foramen is present anteriorly within the fossa (Fig. 1G). Another opening is observed posteroventrally to this foramen, but probably represents an artifact of taphonomy or scavenging. An additional pneumatic fossa, the infradiapophyseal fossa is observed posterior to the base of the rib and ventral to the lamina that connects the diapophysis with the centrum (Fig. 1G). The neural arch fossae observed in this vertebra are similar in form and location to those present in the middle cervicals of the troodontid *Sinornithoides* (Currie & Dong, 2001). The neural arch is rectangular in dorsal view, but slightly constricted in the middle region and expanded in the anterior and posterior portions. The prezygapophyses are markedly projected beyond the anterior end of the centrum and are slightly laterally oriented. On the other hand, the postzygapophyses do not reach beyond the posterior edge of the centrum and present a slight lateral projection. The epipophyses are very small, similar in size to those of the anterior cervicals (Fig. 1E). The neural spine is anteroposteriorly expanded but mediolaterally thin and is centered on the arch. Although its dorsal edge is broken, it clearly was a low structure. The length of the spine is similar to that of *Austroraptor* but differs from the more elongated neural spines of *Deinonychus* and troodontids such as *Sinovenator* and *Byronosaurus* (Ostrom, 1969; Xu, 2002; Makovicky et al., 2003). In *Deinonychus* the neural spines also are taller and more posteriorly located. A short groove is located posterior to the base of the spine and between the postzygapophyses, which corresponds to the insertion for the interspinous ligament. A full sheet of bone with little or no indent bridges between the postzygapophyses and roofs over the neural canal.

The posterior cervical vertebrae differ from the anterior and middle ones in their more laterally projected zygapophyses, imbuing the neural arch with an “X”-shape in dorsal view (Fig. 2A), as in the posterior cervicals of *Microraptor* and *Tsaagan* (Hwang et al., 2002; Norell et al., 2006), the presumed middle cervical of *Mahakala* (Turner, Pol & Norell, 2011), and the anterior and posterior cervicals of *Austroraptor* (Novas et al., 2009), as well as in many other maniraptorans (Makovicky & Sues, 1998). The centrum in these vertebrae is short and does not exceed either the anterior or the posterior extent of the neural arch (Fig. 2B). The last cervical (the 10th) has a narrower centrum than those of the eighth and ninth cervical. A unique trait of the eighth and ninth vertebrae is the presence of paired, ventral ridges on the posterior half of the centrum, which protrude posterolaterally and terminate in small tubercles (Figs. 2E and 2F). These tubercles are more developed in the ninth vertebra, where they have a ventrally facing flat surface. This vertebra also has two carotid processes in the anteroventral zone of the centrum, which define a groove between them, the carotid canal (sulcus caroticus *sensu* Baumel & Witmer, 1993). The 10th cervical also has carotid processes, but these are more developed and bulging, and the carotid canal is therefore less defined. Well-developed carotid processes flanking a defined carotid canal also are present in the posterior cervicals of *Austroraptor*, *Microraptor*, *Sinornithosaurus*, *Tsaagan*, *Troodon, Sinornithoides*, alvarezsaurids, and avialans (Makovicky, 1995; Currie & Dong, 2001; Chiappe, 2002; Hwang et al., 2002; Xu, 2002; Norell et al., 2006; Novas et al., 2009; Tsuihiji et al., 2014), but in *Austroraptor* these processes are present in more anterior cervicals as well. The parapophyses are robust and are laterally projected with respect to the carotid processes from which they are separated by a notch. On the last cervical, two small foramina separated by a thin bony bar lie posterodorsal to the parapophyses and represent pleurocoels. Although the relevant area is not visible on the left side of the ninth cervical centrum because it is covered by the cervical rib, the right side bears a deep fossa right behind the parapophysis with at least two invasive foramina evident. The cervicals of *Austroraptor* also have pleurocoels with two foramina within them. Pneumatic openings are located in similar positions in the posterior cervicals of troodontids as *Sinornithoides*, *Troodon* and *Sinovenator* (Makovicky, 1995; Currie & Dong, 2001), and possibly in the dromaeosaurid *Sinornithosaurus* (Xu, 2002). However, in *Sinornithosaurus* there is only one foramen per side, unlike the paired foramina observed in the cervicals of *Buitreraptor*.

The neural arches of the posterior cervicals are slightly anteroventrally inclined. The prezygapophyses are markedly laterally projected rather than anteriorly, and are connected to the arch by a short peduncle and a thin prezygapodiapophyseal lamina extending posteriorly. A fossa is located dorsally to this lamina and below the inflated midline region of the neural arch. A foramen is located in the anterior part of this fossa, and is more conspicuous in the 10th vertebra (Fig. 2A). Such a fossa is observed in some paravians including *Troodon*, *Sinovenator* and *Liaoningvenator* (Makovicky, 1995; Xu, 2002; Shen et al., 2017b), but is absent in many Laurasian dromaeosaurids such as *Velociraptor* and *Deinonychus* (Ostrom, 1969; Norell & Makovicky, 1999). The diapophyses project from the posteroventral ends of the prezygapophyseal peduncles, and are knob-shaped and lateroventrally extended. The morphology and location of these diapophyses are similar to those present in *Deinonychus* (Ostrom, 1969). The diapophyses of the posterior cervicals of *Austroraptor* differ significantly, as they are more developed and positioned on the lateral zones of the neural arch (Novas et al., 2009).

The eighth and ninth cervical vertebrae preserve the articulation with their corresponding ribs. The ribs have a concave anterior edge, a slightly convex lateral surface and extend posteriorly as a long process with a pointed end. The holotype of *Austroraptor* preserves no cervical ribs, evidence that ribs were not fused with the vertebrae even in the anterior cervicals. The postzygapophyses are less laterally angled but are long, reaching past the lateral and posterior edges of the centrum. Between each pair of postzygapophyses is a broad and triangular fossa just posterior to the low neural arch marking the insertion of the interspinal ligament. Long, divergent postzygapophyses also are observed in other dromaeosaurids such as *Deinonychus* and *Microraptor* (Ostrom, 1969; Hwang et al., 2002), in troodontids such as *Troodon* (Makovicky, 1995), and oviraptorosaurs such as *Microvenator* (Makovicky & Sues, 1998). Unlike these taxa, in *Austroraptor* the postzygapophyses are less divergent and less posteriorly projected. Epipophyses are present on the dorsal surfaces of the postzygapophyses, with a similar morphology to those of the anterior and middle cervicals but slightly more developed, although not overhanging the posterior edge of the postzygapophyses (Figs. 2A and 2B). The relatively modestly developed epipophyses of *Buitreraptor* differ notably from those of larger dromaeosaurids, such as *Deinonychus* and *Velociraptor*, but are similar to those present in *Sinornithosaurus*, *Microraptor* and *Tsaagan* (Hwang et al., 2002; Xu, 2002; Norell et al., 2006), as well as in troodontids (Makovicky & Sues, 1998; Makovicky et al., 2003), ornithomimosaurs (Osmólska, Roniewicz & Barsbold, 1972; Kobayashi & Barsbold, 2005), oviraptorids (Osmólska, Currie & Barsbold, 2004), and alvarezsauroids (Perle et al., 1994; Novas, 1997). It is not possible to ascertain the presence of epipophyses in the holotype cervicals of *Austroraptor* mainly due to the poor preservation of their external surfaces. The neural spines on the posterior cervicals of *Buitreraptor* are low and anteroposterioly long, as in the middle cervicals.

#### Dorsal vertebrae

**Figure 3:**
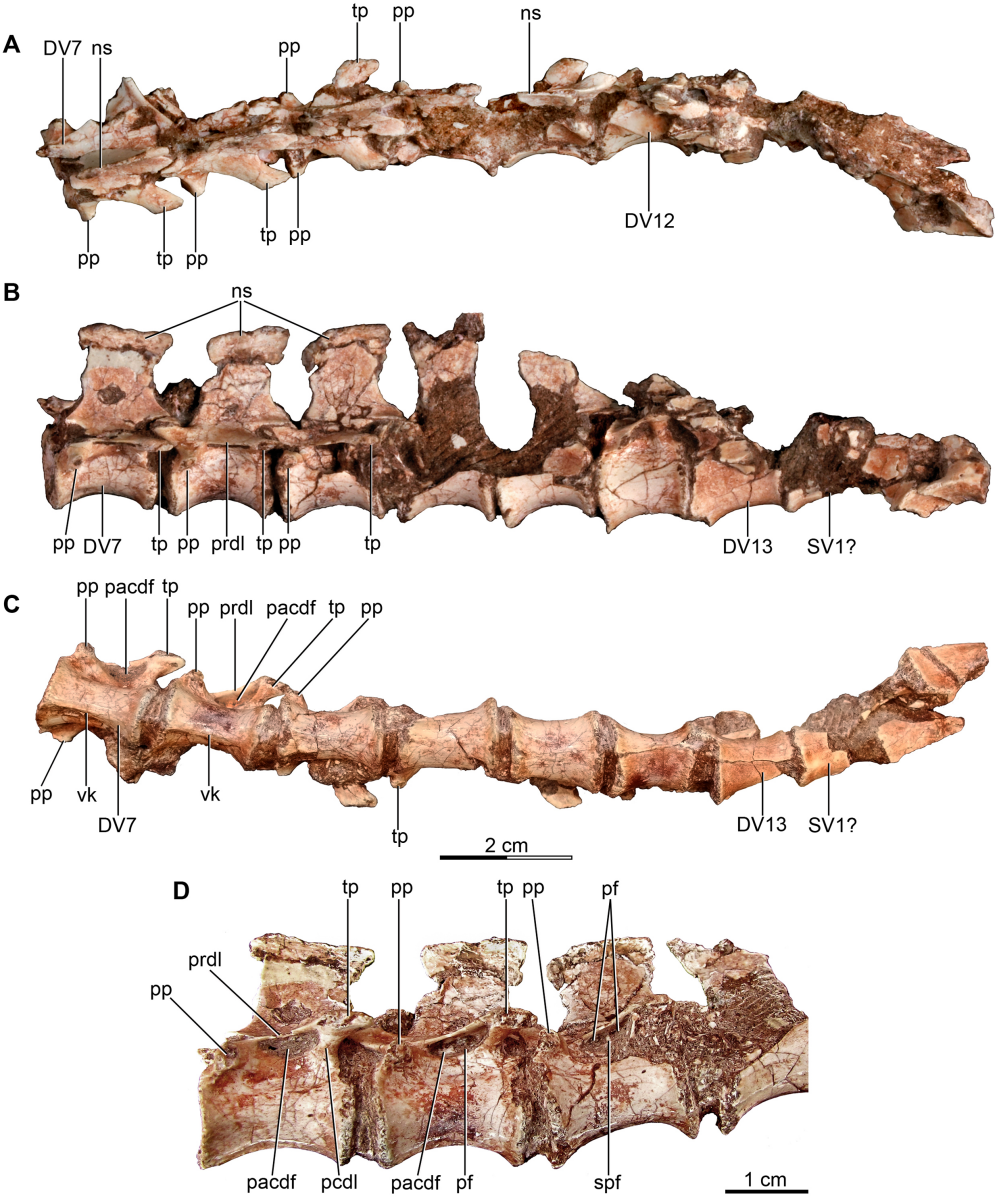
Mid and posterior dorsal vertebrae of the holotype of *Buitreraptor gonzalezorum* (MPCA 245). (A) Dorsal, (B) left lateral and (C) ventral view. (D) Mid dorsal vertebrae, in lateroventral view. Scales: 2 cm for A–C, 1 cm for D. DV, dorsal vertebra; ns, neural spine; pacdf, parapophyseal centrodiapophyseal fossa; pcdl, posterior centrodiapoyseal lamina; pf, pneumatic foramen; pp, parapophysis; prdl, prezygodiapophyseal lamina; spf, septum between pneumatic foramina; SV, sacral vertebra; tp, transverse process; vk, ventral keel.

The dorsal vertebral series of the holotype of *Buitreraptor* is complete, although the most posterior vertebrae are poorly preserved (Figs. 2 and 3), leading to some difficulty in establishing whether the last element belongs to the dorsal series or to the sacrum. If this element is considered to be the first sacral vertebra, then there are a total of thirteen dorsal vertebrae, as in other dromaeosaurids such as *Microraptor* (Hwang et al., 2002) and *Velociraptor* (Norell & Makovicky, 1999). Generally, the dorsals present a fairly homogeneous morphology between them and show no remarkable variation in size, in contrast to the cervical vertebrae.

All dorsal vertebrae are characterized by having elongated, platycoelous, and spool-shaped centra most of which are devoid of pleurocoels. The centra have a length approximately twice their height (Table S1), similar to *Rahonavis*, *Microraptor*, *Sinornithosaurus*, *Sinovenator* and *Archaeopteryx* (Wellnhofer, 1993; Hwang et al., 2002; Xu, 2002), but differing from large-bodied dromaeosaurids like *Deinonychus* and *Austroraptor*, which have dorsal centra taller than long (Ostrom, 1969; Novas et al., 2009), and mid-sized taxa like *Velociraptor* and *U. comahuensis* which have centra that have approximately the same length as height (Novas & Puerta, 1997; Norell & Makovicky, 1999). The intervertebral articular surfaces are circular in end view and always have a horizontal diameter that represents 50% or more of the length of the centra. The diapophyses are well-developed, the neural spines are tall and rectangular.

Pneumatic foramina are only observed on the centra of the first and second dorsal vertebrae (Figs. 2B–2D). These foramina are small and are located on the anteroventral part of the lateral surface of each centrum, ventral and slightly posterior to the parapophyses. Pneumatic foramina in the dorsal centra are not reported in the specimen MPCN-PV-598 (Novas et al., 2018). Other unenlagiines seems to have invasive pneumatic foramina in all dorsals, located within deep fossae on the lateral surfaces of the centra. In *Unenlagia* foramina are observed in the anteriormost and the posteriormost dorsal vertebrae preserved. In *Rahonavis*, there is a small pneumatic foramen on the anteriormost preserved dorsal, which is likely the first or second of the series whereas more posterior dorsal centra lack foramina (Forster et al., 1998). In *Austroraptor* the second and fourth dorsal vertebrae have well-developed, paired foramina on their centra, with each pair separated by a bony lamina. The presence of these traits in more posterior dorsals of *Austroraptor* cannot be corroborated due to lack of preservation.

The three first dorsal vertebrae of the *Buitreraptor* holotype skeleton bear hypapophyses that project from the anteroventral surfaces of the centra (Figs. 2B and 2E) as convex, asymmetrically developed keels. Although the apices of the first two are not well preserved, they appear to grade from deepest in front to shallower farther posteriorly. Unlike the bladelike hypapophyses of some paravians, including many avian species, the bases of the hypapophyses of the two anteriormost dorsals are transversely wide. Hypapophyses are also observed in other dromaeosaurids as *Velociraptor*, *Saurornitholestes*, *Deinonychus*, *Rahonavis*, *Bambiraptor*, *Sinornithosaurus*, *Microraptor* and *Mahakala* (Ostrom, 1969; Sues, 1978; Forster et al., 1998; Norell & Makovicky, 1999; Xu, 2002; Burnham, 2004; Makovicky, Apesteguía & Agnolín, 2005; Turner, Pol & Norell, 2011), but they are generally common in coelurosaurs (Baumel & Witmer, 1993; Perle et al., 1994; Makovicky, 1995; Barsbold et al., 2000; Currie & Dong, 2001; Makovicky et al., 2003). In *Austroraptor* the two preserved dorsals, identified as anterior dorsals, have well-developed keels that may be homologous with hypapophyses, although each has a uniform height along the length of its respective centrum (Novas et al., 2009). The hypapophysis of the second vertebra of the *Buitreraptor* holotype is broken close to its base, but seems to have been more robust than those of the first and third vertebrae, as its base is significantly wider. Moreover, the centrum of the second dorsal is less transversely compressed (Fig. 2E). From the fourth dorsal backwards the centra do not present a hypapophysis, but bear a ventral longitudinal keel which extends along the midline of the centra. These keels become progressively less prominent towards the posterior dorsals, and completely disappear by the 11th one. In *U. comahuensis* the anteriormost dorsal preserved exhibits a small anteroventral process, similar to the middle dorsals of *Buitreraptor*, but it does not extend posteriorly as a keel (Novas & Puerta, 1997). *Rahonavis* has a well-developed hypapophysis in the anteriormost preserved dorsal whereas the middle dorsal vertebrae have a ventral keel, and the posteriormost preserved dorsal, which likely was adjacent to the sacrum, has a rounded ventral surface of the centrum.

The parapophyses are located on the anterolateral surfaces of the centra in the anterior dorsals, slightly posteriorly removed from the rim of the articular surfaces. Farther posteriorly, they acquire a more dorsal position and by the fifth vertebra they are located entirely on the neural arch, anterior to and only slightly ventral to the diapophyses (Fig. 2B). The parapophyseal peduncle has a broad, triangular base in the first two dorsal vertebrae, when viewed ventrally. An oblique, robust ridge of bone extends from the anteroventral end of the parapophyses to the rim of the anterior intercentral articulation forming the anterolateral edge of this triangle. From the sixth dorsal back, the parapophysis is located closer to the anterior face of the centrum, and the parapophysis is more stalk-like in ventral view.

The parapophyses are raised on laterally projected peduncles as is observed in *U. comahuensis*, *Austroraptor* and other dromaeosaurids (Ostrom, 1969; Norell & Makovicky, 1999; Xu, 2002; Makovicky, Apesteguía & Agnolín, 2005), but also in alvarezsauroids (e.g., *Patagonykus puertai*, Novas, 1997; *Mononykus olecranus*, Perle et al., 1994), avialans (e.g., *Confuciusornis sanctus*, Chiappe et al., 1999), and some troodontids (i.e., *Talos sampsoni* and *Mei long*; Xu & Norell, 2004; Zanno et al., 2011).

The neural arch of the first dorsal vertebra is not preserved. The neural arch of the second dorsal is broader in dorsal view than in the remaining dorsals, with laterally projected zygapophyses that overhang the lateral centrum faces. The postzygapophyses diverge markedly from the midline and a triangular spinopostzygapophyseal fossa is located between them. Epipophyses are absent in this and all other dorsal vertebrae preserving the arch region. The zygapophyses of the remaining dorsals are generally small and approximately parallel to the anteroposterior axis. The presence of a hyposphene–hypantrum accessory articulation complex is not confirmed because almost all the dorsals are articulated, but in the sixth vertebra, which is disarticulated from the seventh one, a hyposphene does appear to be present. The dorsals of *U. comahuensis*, *U. paynemili*, *Austroraptor* and also *Rahonavis* exhibit hyposphenes (Calvo, Porfiri & Kellner, 2004; Forster et al., 1998; Novas & Puerta, 1997; Novas et al., 2009), formed by two, shallow vertical laminae ventrally connected by a horizontal groove above the neural canal. Hyposphenes with a similar morphology are present in *Deinonychus* and *Patagonykus* (Ostrom, 1969; Novas, 1997), but in many other theropods this structure is formed by a single, deep, and vertical lamina extending between the postzygapophyses, as in *Tyrannosaurus*, *Allosaurus*, *Carnotaurus*, *Masiakasaurus*, and ornithomimosaurs (Madsen, 1976; Bonaparte, Novas & Coria, 1990; Carrano, Sampson & Forster, 2002; Brochu, 2003; Makovicky, Kobayashi & Currie, 2004).

In the second dorsal, only the right transverse process is preserved. It projects laterally as well as slightly dorsally and posteriorly, and is located approximately at the same level as the articular surfaces of the prezygapophyses. It has a spatulate outline in dorsal view. The transverse processes of the following dorsals also are laterally projected, but exhibit a stronger posterolateral orientation and are not spatulate. The transverse processes diminish in length caudally and become gradually more backswept. From at least the seventh dorsal, the transverse processes are slightly offset ventrally so that they are below the level of the zygapophyses and the diapophyseal articulation is almost level with the parapophysis (Figs. 2A–2D and 3A–3C). In *Austroraptor* the diapophyses have a similar inclination to that of the anterior dorsals of *Buitreraptor*, whereas in *U. comahuensis* they are less inclined. From the fifth dorsal backward, the transverse processes are connected to the prezygapophyses by wide prezygodiapophyseal laminae (prdl, following Wilson et al., 2011). A short lamina joins the parapophysis to the ventral surface of the prezygodiapophyseal lamina, and is here interpreted as the paradiapophyseal lamina (ppdl, *sensu* Wilson et al., 2011). A sharply defined fossa lies ventral to the prezygodiapophyseal lamina (Figs. 3C and 3D), delimited anteriorly by the paradiapophyseal lamina and posteriorly by the posterior centrodiapoyseal lamina (pcdl), and it corresponds to the parapophyseal–centrodiapophyseal, or infraprezygapophyseal fossa (pacdf, *sensu* Wilson et al., 2011). As a result of the ventral displacement, the parapophyseal–centrodiapophyseal fossa (=infradiapophyseal) fossa is very narrow and appears slit-like in lateral aspect when compared to other paravians. A similar slit-like appearance of the parapophyseal–centrodiapophyseal fossa is seen in a posterior dorsal of *Rahonavis* (Forster et al., 1998). Within these fossae a single foramen, or sometimes two foramina separated by a thin bony bar, are observed and are possibly pneumatic in nature (Fig. 3D). Anterior to the paradiapophyseal lamina, a shallow fossa, marks the dorsal surface of the parapophyseal stalk and is defined anteriorly by the prezygoparapophyseal lamina (prpl) and dorsally by the prezygodiapohyseal lamina. This weakly developed fossa is here interpreted as the prezygapophyseal–centrodiapophyseal fossa (prcdf, *sensu* Wilson et al., 2011). The centrodiapophyseal–postzygapophyseal fossa is small and largely obscured by the articulated nature of the vertebral column. It is evident on the fourth dorsal vertebra, where it forms a shallow depression along the posterior edge of the transverse process.

In the posterior dorsals, the paradiapophyseal laminae as well as the prezygapophyseal–centrodiapophyseal fossa are progressively reduced and disappear. Parapophyseal–centrodiapophyseal and prezygapophyseal–centrodiapophyseal fossae are also present but are more prominent in *Austroraptor* (MML 195), and both species of *Unenlagia* (MCF PVPH 78; MUCPv 349). Furthermore, in *Austroraptor* and *U. comahuensis* the prezygapophyseal–centrodiapophyseal fossa increases in size relative to the parapophyseal–centrodiapophyseal fossa moving from the anterior dorsals to the posterior ones. This trend is opposite to that observed in *Buitreraptor*, in which the prezygapophyseal–centrodiapophyseal fossa decreases in size until it disappears in the posterior dorsals. In *Rahonavis* the anteriormost dorsal has a very large prezygapophyseal–centrodiapophyseal fossa with a much reduced parapophyseal–centrodiapophyseal fossa ventral to it. In more posterior dorsals the parapophyseal–centrodiapophyseal fossa is relatively larger, but still smaller than the prezygapophyseal–centrodiapophyseal fossa (FMNH PR 2830).

The neural spines are tall and transversely compressed. In the more anterior dorsals the height of the spine is comparable to that of the centrum, but increases progressively in the middle- and posterior dorsals to be at least twice the depth of the centrum (Figs. 2B and 3B). The distal part of the neural spines is anteroposteriorly expanded acquiring a slightly fan-shaped aspect, as is also observed in MPCN-PV-598, and similar to the neural spines of *U. comahuensis*, *Microraptor*, *Sinornithosaurus* and *Sinovenator* (Novas & Puerta, 1997; Xu, 2002), but they lack the transverse expansions into the “spine tables” observed in *Velociraptor*, *Deinonychus*, *U. comahuensis* and *Austroraptor* (Ostrom, 1969; Novas & Puerta, 1997; Norell & Makovicky, 1999; Novas et al., 2009). The posterior border of the neural spines never extends past the posterior border of the centrum in *Buitreraptor*, in contrast to the condition of the dorsals of *Austroraptor* and *Rahonavis*, and also the anterior dorsal preserved of *U. comahuensis* (Novas & Puerta, 1997; Forster et al., 1998; Novas et al., 2009). Spinoprezygapophyseal and spinopostzygapophyseal fossae located at the anterior and posterior sides of the base of the neural spine respectively, are present in *Buitreraptor*, but are small. On the other hand, in *U. comahuensis*, *U. paynemili* and *Austroraptor* these fossae are much more developed (Novas & Puerta, 1997; Calvo, Porfiri & Kellner, 2004; Novas et al., 2009).

#### Sacral vertebrae

**Figure 4:**
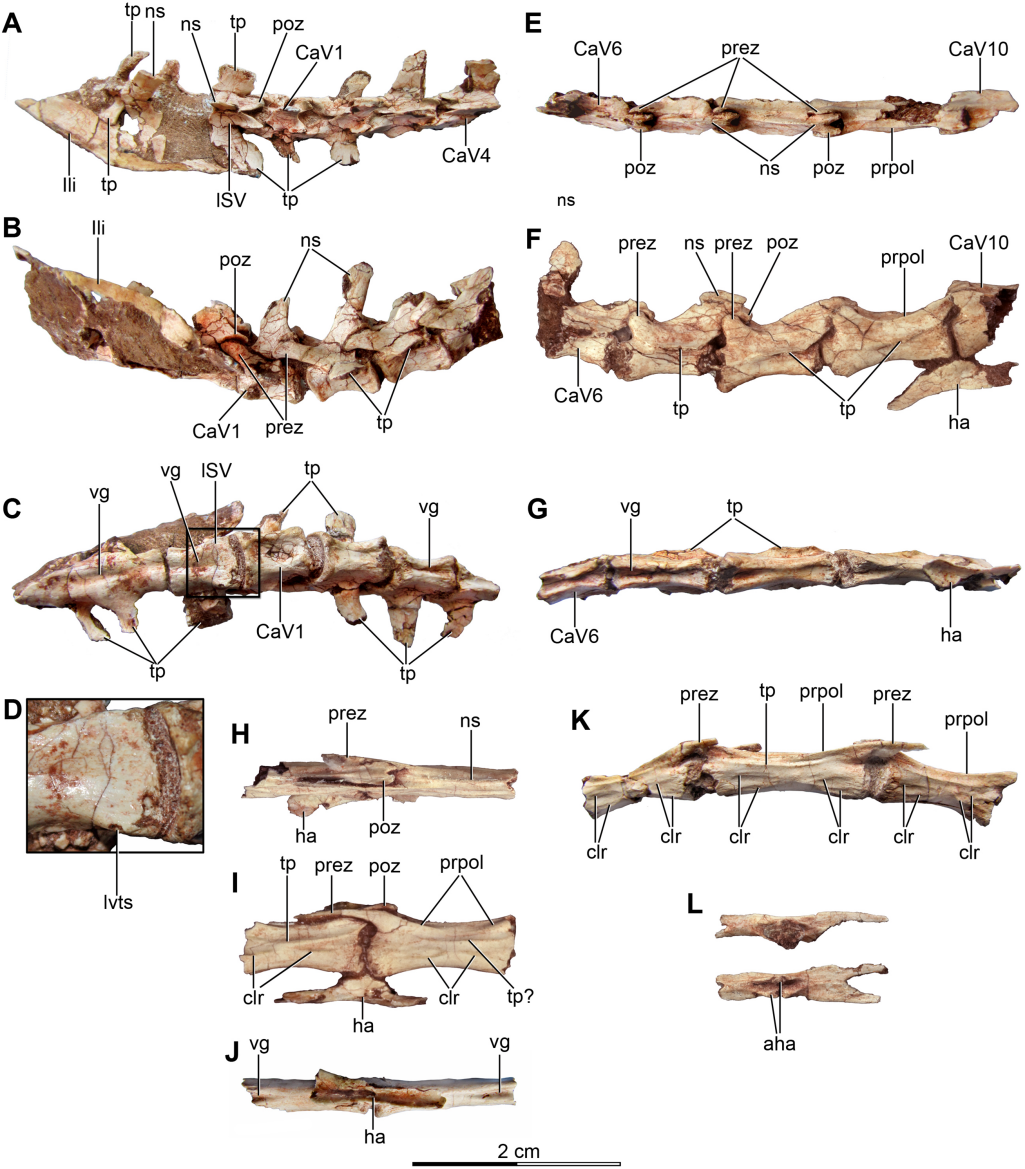
Sacral and caudal vertebrae and chevrons of the holotype of *Buitreraptor gonzalezorum* (MPCA 245). (A–C) Sacrum and anterior caudal vertebrae, in (A) dorsal, (B) left lateral (C) and ventral view. (D) Detail of the box inset in C, showing the lateroventral tubercles of the last sacral vertebra. (E–G) 6th–10th caudal vertebrae, in (E) dorsal, (F) left lateral and (G) ventral view. (H–J) 10th and 11th caudal vertebrae, in (H) dorsal, (I) left lateral and (J) ventral view. (K) Distal caudal vertebrae, in left lateral view. (L) Distal chevrons, in dorsal view. Scale: 2 cm for all elements, except for D. aha, articular surfaces of the haemal arch; CaV, caudal vertebra; clr, convergent lateral ridges; ha, haemal arch; Ili, ilium; lSV, last sacral vertebra; lvts, lateroventral tubercle of the last sacral vertebra; ns, neural spine; poz, postzygapophysis; prez, prezygapophysis; prpol, “prezygopostzygapophyseal” lamina; tp, transverse process; vg, ventral groove.

**Figure 5:**
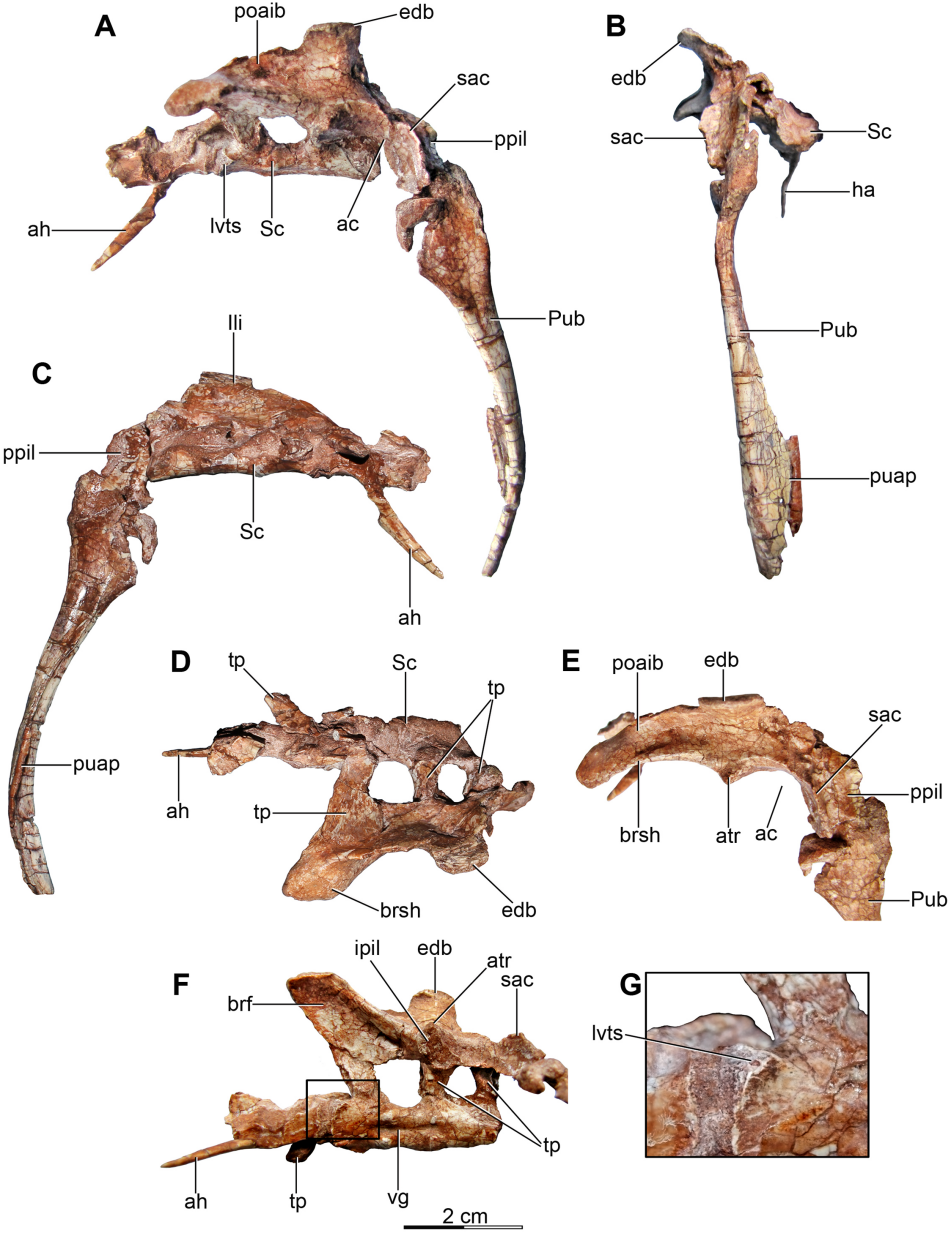
Sacrum and pelvic girdle of the referred specimen of *Buitreraptor gonzalezorum* (MPCA 238). (A–C) Sacrum and pelvic girdle, in (A) right lateral, (B) anterior and (C) left lateral view. (D–F) Detail of the sacrum and ilium, in (D) dorsal, (E) right lateral and (F) ventral view. (G) Detail of the box inset in F, showing the lateroventral tubercle of the last sacral vertebra. Scale: 2 cm for all elements, except for G. ac, acetabulum; ah, haemal arch; atr, antitrochanter; brf, brevis fossa; brsh, brevis shelf; edb, everted dorsal border of the ilium; Ili, ilium; ipil, ischiadic peduncle of the ilium; lvts, lateroventral tubercle of the last sacral vertebra; poaib, postacetabular iliac blade; ppil, pubic peduncle of the ilium; puap, pubic apron; Pub, pubis; sac, supracetabular crest; Sc, sacrum; tp, transverse process, vg, ventral groove.

The sacrum is partially preserved both in the holotype and the referred specimen MPCA 238 (Figs. 4A–4D and 5). MPCN-PV-598 also has preserves the sacrum, but it is not described here. The sacral vertebral centra are incompletely fused, and their exact number is unclear mainly due to the poor preservation of the anterior ones. The last sacral is recognizable because it is articulated with the first caudal vertebra and in turn partially fused to three preceding sacral vertebrae in the holotype. Moreover, the last sacral has two small tubercles that project from the posterolateral borders of the ventral surface, which also are observed in the last sacral vertebra of MPCA 238 (Figs. 4C, 4D and 5A, 5F, 5G). Similar tubercles are observed in sacrals of many other paravians such as *Sinovenator* and *Microraptor*. The most anterior of the three preceding vertebrae is broken, but fits exactly with the partial centrum of the preserved sacral at the end of the articulated posterior dorsal series (Fig. 3). Thus, it appears five sacral vertebrae are present in *Buitreraptor*, a count also recorded in *Velociraptor*, *Sinornithosaurus* and *Microraptor* (Norell & Makovicky, 1997; Hwang et al., 2002; Xu, 2002), troodontids such as *Mei* and *Sinovenator* (Xu et al., 2002; Xu & Wang, 2004a), and in *Archaeopteryx* (Ostrom, 1975; Wellnhofer, 1974, 1992). Six sacral vertebrae are reported in the specimen MPCN-PV-598, which is larger than either the holotype or MPCA 238 specimens (Novas et al., 2018). Ontogenetic variation in the degree of sacral fusion has been observed in several theropod taxa including *Velociraptor*, coelophysoids, and oviraptorosaurs (Norell & Makovicky, 1999; Griffin & Nesbitt, 2016; Osmólska, Currie & Barsbold, 2004), so we interpret this difference as reflecting size- or growth-related variation. Six sacral vertebrae are present in other paravians such as *Mahakala*, *Rahonavis*, *Saurornithoides* and *Troodon* (Makovicky, 1995; Forster et al., 1998; Rauhut, 2003; Makovicky & Norell, 2004; Norell & Makovicky, 2004; Turner et al., 2007; Norell et al., 2009).

In the holotype the four posterior sacrals are articulated with the left ilium, whereas in MPCA 238 the last three sacrals are articulated with the right ilium. The posterior portion of the ventral surface of the sacrum is marked by a shallow, longitudinal sulcus (Figs. 4C and 5F), as is common in many coelurosaurs including *Ornitholestes*, ornithomimosaurs, therizinosaurs, oviraptorosaurs, dromaeosaurids, troodontids and avialans (Makovicky, Kobayashi & Currie, 2004; Zanno, 2010; Xu et al., 2002; Rauhut, 2003). This ventral sulcus is also observed in the specimen MPCN-PV-598, and this trait is also present in *Rahonavis*, whereas in *Unenlagia* the ventral surface of the sacrum is flat (Federico A. Gianechini, 2010, personal observation). The last sacral vertebral centrum of the holotype is not fully fused to the remaining sacral centra and it is slightly expanded posteroventrally. Unlike the holotype, the last sacral of MPCA 238 is completely fused to the rest of the sacrum (Fig. 5F). The lateral surfaces of the centra are eroded, but seem to be smooth and devoid of pleurocoels, at least in the posterior sacrals, a feature observed also in MPCN-PV-598. The lack of pleurocoels in the sacral vertebrae can also be observed in some other dromaeosaurids, such as *Sinornithosaurus* (Xu, 2002), but pleurocoels are observed in the anterior sacrals of some larger species including *Velociraptor* and *Saurornitholestes* (Norell & Makovicky, 1997). MPCN-PV-598 reveals a progressive decrease in height of the sacrals towards the caudal end (Novas et al., 2018), although this trait cannot be confirmed in either the holotype or MPCA 238, due to the poor preservation of the neural arches and dorsal portions of the centra.

The transverse processes of the posterior sacrals increase in length towards the caudal end, indicating that the ilia diverge from each other posteriorly, as is observed in many maniraptorans (Makovicky & Norell, 2004; Norell & Makovicky, 2004). In MPCA 238 these processes are constricted close to their bases, but are anteroposteriorly and dorsoventrally expanded distally. The transverse processes of the last sacral show the largest degree of distal expansion in the anteroposterior plane but, in contrast to the other sacral transverse processes, are dorsoventrally flattened (Figs. 5D and 5F) as in *Microraptor* (Hwang et al., 2002). In dorsal view, they exhibit a spatulate outline, with a thin and laminar distal end that contacts the medial surface of the postacetabular iliac blade at the level of the brevis fossa. The widened distal portion of this process has a concave ventral surface which is continuous with the surface of the brevis fossa, a condition not observed in *U. comahuensis* or *Rahonavis* (Novas & Puerta, 1997; Forster et al., 1998).

Most sacral zygapophyses are not preserved; only the postzygapophyses of the last sacral in the holotype can be described (Figs. 4A and 4B). These structures are laterally curved and separated from each other, leaving a triangular space between them. The neural spines are preserved only on the last two sacrals of the holotype, and exhibit a rectangular shape (Fig. 4A). They are not fused, unlike the sacral neural spines of other dromaeosaurids such as *Velociraptor* and *Microraptor* (Norell & Makovicky, 1997; Hwang et al., 2002) which are fused to form a continuous lamina. The sacral neural spines also appear to be unfused in *Rahonavis* (FMNH PR 2830 [cast of UA2]).

#### Caudal vertebrae

Approximately 15 caudal vertebrae are preserved in the holotype, from the proximal, middle and distal parts of the tail, and remain articulated (Figs. 4A–4C and 4E–4K). MPCA 238 preserves the first two caudal vertebrae, the first of which is articulated with the sacrum (Figs. 5A, 5C, 5D and 5F). The total number of vertebrae is difficult to assess although the tail likely would have had more than 20 vertebrae, as occurs in other paravians such as *Velociraptor*, *Bambiraptor*, *Deinonychus*, *Microraptor*, *Tianyuraptor*, *Gobivenator* and *Jeholornis* (Ostrom, 1969; Norell & Makovicky, 1999; Burnham et al., 2000; Xu, Zhou & Wang, 2000; Hwang et al., 2002; Xu, 2002; Zhou & Zhang, 2003a; Burnham, 2004; Zheng et al., 2010; Tsuihiji et al., 2014). The length of the vertebrae progressively increases along the caudal series (Table S1). The posterior caudals also become lower and more transversely compressed. The caudal series of the holotype specimen is complete from the first through the fourth vertebrae. The fifth one appears to be fragmentary and only preserves the neural spine. This is joined to a group of five articulated vertebrae, from the sixth until the 10th. The 10th caudal is broken near the anterior end and the other part is articulated to the 11th caudal (Figs. 4H–4J). The transition from the anterior caudals (i.e., short, boxy centra with well-developed transverse processes and neural spines) to the posterior caudals (i.e., more elongated centra with poorly developed or vestigial transverse processes and neural spines), is observed between the eighth and the 10th vertebrae (Fig. 4F). A similar position for the transition point in the caudal series is observed in *Rahonavis* (Forster et al., 1998), other dromaeosaurids such as *Deinonychus* (Ostrom, 1969) and troodontids such as *Sinornithoides*, *Mei* and *Daliansaurus* (Currie & Dong, 2001; Gao et al., 2012; Shen et al., 2017a); whereas in microraptorines, such as *Microraptor* and *Zhongjianosaurus* (Hwang et al., 2002; Senter et al., 2012; Pei et al., 2014; Xu & Qin, 2017) this transition point occurs more anteriorly (between caudal 5 and 7) as mentioned by Motta, Brissón Egli & Novas (2018). In contrast, in *Mahakala* the transition point has been identified as occurring between caudals 11 and 12 (Turner, Pol & Norell, 2011). In the recent description of the tail of *Buitreraptor*, Motta, Brissón Egli & Novas (2018) misinterpreted the last sacral vertebra as the first caudal in the holotype, so the vertebra that they describe as the second caudal is actually the first. This error leads them to interpret the transition point in the caudal series as occurring between caudals 9 and 11.

The ventral surface of the anterior centra are marked by longitudinal sulci, which are more defined in the anterior vertebrae but turn shallower distally and almost disappear in the most posterior preserved vertebrae (Figs. 4C, 4G and 4J). Ventral sulcus are present on the caudal vertebrae in many theropods (Rauhut, 2003) and other archosaurs, and can be observed in alvarezsauroids (Alifanov & Barsbold, 2009), ornithomimosaurs (Osmólska, Roniewicz & Barsbold, 1972; Kobayashi & Barsbold, 2005), oviraptorosaurs (Barsbold et al., 2000; Xu et al., 2007), *Allosaurus* (Madsen, 1976), and non-tetanuran theropods as *Ceratosaurus* (Madsen & Welles, 2000).

The anterior caudals, especially the first and second, have a centrum with a quadrangular transverse section, as also is observed in *Mahakala*, *Rahonavis*, *Archaeopteryx*, and troodontids such as *Saurornithoides* (Gauthier, 1986; Forster et al., 1998; Rauhut, 2003; Makovicky & Norell, 2004; Norell & Makovicky, 2004; Norell et al., 2009; Turner, Pol & Norell, 2011). The first caudal vertebra is similar to the sacrals, both in length and transverse compression, and differs from the remaining caudals because the ventral surface is widely concave and without a sharply defined sulcus (Fig. 4C), resembling the first caudal of *Rahonavis*. The posteroventral border of the centrum has two points of articulation for the haemal arch as well-developed protuberances. In the succeeding caudals, the ventral sulcus is strongly marked and deep and the ventral surface is constricted at midlength with more pronounced lateroventral borders. In the eighth caudal the sulcus becomes shallower and less defined at midlength (Fig. 4G), whereas in more posterior caudals it is even more reduced to anterior and posterior depressions of the ventral surface, separated by a non-depressed central zone (Fig. 4J). The posterior caudals are strongly compressed transversely, in contrast to those of *Velociraptor*, *Deinonychus* and *Rahonavis* (Ostrom, 1969; Forster et al., 1998; Norell & Makovicky, 1999). All the caudal vertebrae are devoid of pleurocoels, as in *Rahonavis* and other dromaeosaurids like *Deinonychus*, *Velociraptor* and *Bambiraptor* (Ostrom, 1969; Forster et al., 1998; Norell & Makovicky, 1999), and *Allosaurus* (Madsen, 1976), but in contrast to oviraptorosaurs (Osmólska, Currie & Barsbold, 2004), neovenatorids (Novas, Ezcurra & Lecuona, 2008; Sereno et al., 2008), and carcharodontosaurids (Stromer, 1931).

The transverse processes are elongate in the anterior caudals and approximately rectangular in shape, as in *Rahonavis*. In the first four caudal vertebrae, the processes are located at the midlength of the neural arch and are horizontally and slightly posteriorly projected (Figs. 4A–4C). The posterior inclination is similar to that of the transverse processes of *Microraptor* (Hwang et al., 2002), but it is not as marked as in *Velociraptor* and *Deinonychus* (Ostrom, 1969; Norell & Makovicky, 1997). From the fourth caudal moving posteriorly, the processes are more posteriorly located and by the fifth caudal they also acquire a more ventral position, until finally they are located on the sides of the centra (Fig. 4F). This shift in the position of the transverse processes is also observed in the caudals of *Rahonavis*, *Deinonychus* and *Saurornithoides* (Norell et al., 2009). From the eighth caudal moving posteriorly, the processes decrease in size and are shorter and anteroposteriorly extended. Anterior and posterior ridges extending horizontally along the lateral surface of the neural arch connect the transverse processes to the bases of the prezygapophyses and postzygapophyses, respectively (Figs. 4F and 4I). In the posterior caudals the transverse processes become almost completely absent (Fig. 4K) and are represented by shallow ridges extended between the bases of the prezygapophyses and postzygapophyses, as also occurs in the specimen MPCN-PV-598 and in *Rahonavis*.

The most posterior caudals are extremely elongate and transversely compressed (Fig. 4K). The lateral surfaces bear the reduced transverse process, as was discussed above. Ventral to the ridges representing the vestige of the transverse process, the lateral surface of the centrum is traversed by two low but conspicuous ridges that extend from the anteroventral and posteroventral parts of the lateral surface of the centrum, respectively. These ridges are dorsally inflected so they converge on each other at midlength but do not contact (Fig. 4I). These ridges have also been described in caudal vertebrae of MPCN-PV-598 (Motta et al., 2016; Motta, Brissón Egli & Novas, 2018) and in caudals of *Rahonavis* (Gianechini, 2014). More posterior caudal vertebrae of the holotype bear an additional pair of ridges, dorsal to those already described. They are posteroventrally and anteroventrally inclined, respectively, and also converge at midlength of the lateral surface of the centrum, without contacting each other. A triangular space with a slightly concave surface is delimited between the dorsal and ventral ridges, at the anterior and posterior ends of the lateral surface of the centrum (Fig. 4K). These additional ridges are also observed in posterior caudals of MPCN-PV-598 (Motta et al., 2016; Motta, Brissón Egli & Novas, 2018), but not in caudals of *Rahonavis* (Gianechini, 2014).

The prezygapophyses of the anterior caudals are elongated and project anteriorly, especially on the second caudal, and exceed the anterior border of the centrum (Figs. 4A and 4B). Posterior to the second caudal vertebra, the prezygapophyses decrease in length and are very short in the eighth to 10th caudal, and do not reach beyond the anterior border of the centra (Figs. 4E and 4F), a condition also observed in the specimen MPCN-PV-598 (Motta, Brissón Egli & Novas, 2018; Novas et al., 2018). They are also more dorsally directed and have a spatulate form. From the first to the 10th vertebra the prezygapophyses are located close to the midline and vertically directed so that the articular surfaces are mainly medially directed. Distal to the 10th vertebra, the caudals have more elongate prezygapophyses shaped like bony rods that extend anteriorly almost to the middle of the preceding vertebra (Figs. 4H, 4I and 4K). The articular surface of each is located near the base of the prezygapophysis and is medially directed as in *Deinonychus* (Ostrom, 1969). Even though *Buitreraptor* shows elongated prezygapophyses, these do not reach the extreme lengths observed in most dromaeosaurids, such as *Velociraptor*, *Deinonychus*, *Saurornitholestes*, *Utahraptor*, *Achillobator*, *Graciliraptor*, *Microraptor*, *Sinornithosaurus*, *Tianyuraptor* and *Changyuraptor* (Ostrom, 1969; Kirkland, Gaston & Burge, 1993; Currie, 1995; Norell & Makovicky, 1999; Perle, Norell & Clark, 1999; Xu, Wang & Wu, 1999; Xu, Zhou & Wang, 2000; Hwang et al., 2002; Xu, 2002; Xu & Wang, 2004b; Zheng et al., 2010; Han et al., 2014). In troodontids such as *Sinovenator*, and some basal avialans such as *Jeholornis* (Xu et al., 2002; Zhou & Zhang, 2002), the prezygapophyses of the posterior caudals exhibit a similar degree of elongation to those of *Buitreraptor*. In contrast, in *Rahonavis* and basal avialans such as *Archaeopteryx*, the prezygapophyses of posterior caudals are much shorter than those present in *Buitreraptor* (Wellnhofer, 1974; Forster et al., 1998). Strikingly, in the specimen MPCN-PV-598 the 10th and subsequent caudals do not have elongated prezygapophyses like those of the holotype (Motta, Brissón Egli & Novas, 2018; Novas et al., 2018). This is possibly due to lack of preservation or loss during preparation of the delicate rod-like extensions, or less likely, could represent an intraspecific variation.

The postzygapophyses are markedly shorter than the prezygapophyses. In the anterior caudals they not reach past the posterior border of the centrum and the articular surfaces are laterally and slightly ventrally directed (Figs. 4A and 4B). They are connected to the neural spine through the spinopostzygapophyseal laminae, and a small spinopostzygapophyseal fossa (spof, *sensu* Wilson et al., 2011) is observed between these laminae. The postzygapophyses approach each other gradually in the posterior vertebrae, so the spinopostzygapophyseal fossa decreases in size. They extend beyond the posterior border of the centrum and acquire a more dorsal position posterior to the fifth vertebra. In “middle” caudals (i.e., the eighth and more distal caudals), the postzygapophyses have forked posterior ends, which are dorsoventrally compressed and slightly laterally expanded (Fig. 4H). In the “middle” caudals, a “prezygopostzygapophyseal” ridge extends between the pre- and postzygapophyses, but it is not continuous and fades in the midsection of each vertebra (Figs. 4E, 4F, 4I and 4K). This ridge is observed in the specimen MPCN-PV-598 (Novas et al., 2018), and is also present in *Rahonavis* and in the posterior caudals of troodontids such as *Saurornithoides*, *Byronosaurus*, *Mei* and *Sinovenator*, although the ridge is continuous in these troodontid taxa (Xu, 2002; Makovicky et al., 2003; Xu & Norell, 2004; Norell et al., 2009).

The neural spines of the proximal caudal vertebrae are tall and prominent whereas in the distal vertebrae they are shallower and ultimately become vestigial. In the most proximal caudals the spines are rectangular in shape and posteriorly inclined so that they overhang the posterior border of the centrum (Figs. 4A and 4B). The spines gradually elongate and decrease in height posteriorly, and from the eighth vertebra to the last preserved ones they are reduced to a vestigial structure as a longitudinal and shallow ridge running along the dorsal surface of the vertebrae (Figs. 4E and 4H). In other dromaeosaurids, such as *Velociraptor*, *Mahakala* and *Rahonavis*, and some troodontids as *Sinornithoides*, the neural spines of the distal caudals also present a similar morphology to those of *Buitreraptor* (Russell & Dong, 1993; Norell & Makovicky, 1997; Turner et al., 2007). On the other hand, in other dromaeosaurids as *Deinonychus* and *Graciliraptor*, and in avialans as *Archaeopteryx*, the distal caudals have a smooth dorsal surface (Ostrom, 1969; Wellnhofer, 1992; Xu, 2002). In most troodontids the distal caudal vertebrae lose their neural spines, as in *Troodon*, *Sinovenator*, *Sinusonasus*, *Mei*, *Byronosaurus* and *Gobivenator*, and instead exhibit a longitudinal sulcus along the dorsal surface (Russell, 1969; Xu, 2002; Makovicky et al., 2003; Xu & Norell, 2004; Xu & Wang, 2004a; Tsuihiji et al., 2014).

#### Haemal arches

Six haemal arches are preserved in the holotype specimen, although only two are articulated with the vertebrae, specifically between the eighth and ninth caudals and between the ninth and 10th caudals (Figs. 4F–4J). The remaining haemal arches are isolated (Fig. 4L). Only a single chevron was preserved in MPCA 238, representing the first one and it is articulated with the tail vertebrae (Fig. 5). It is dorsoventrally elongated as a straight rod, slightly triangular in lateral view, and posteroventrally inclined so that it completely underlaps the more posterior vertebra that it articulates with (Fig. 5). However, this morphology is common in the first haemal arch in many coelurosaurs, as in dromaeosaurids (Ostrom, 1969; Norell & Makovicky, 1997, 1999; Forster et al., 1998; Xu, 2002), troodontids (Russell, 1969; Russell & Dong, 1993; Currie & Dong, 2001; Norell et al., 2009), ornithomimosaurs (Osmólska, Roniewicz & Barsbold, 1972), and tyrannosaurs (Brochu, 2003).

The chevrons of the “middle” caudals bear two dorsal processes, which articulate with the vertebrae and delimit the haemal canal. The bodies of these chevrons are anteroventrally and posteroventrally expanded in two dorsoventrally compressed projections that terminate in forked ends in some of the isolated chevrons. The ventral surface of each bears a longitudinal sulcus. The anterior and posterior ends of the articulated ninth chevron in the holotype specimen appear to underlap almost half the length of each of the centra it articulates with, and it is still missing the tips of the anterior and posterior extensions. It is therefore likely that the tips of consecutive chevrons contacted each other (Figs. 4F–4J and 4L). The forked ends of the anterior process extend as two short and pointed rods, as in other paravians (Russell, 1969; Wellnhofer, 1974, 1992; Russell & Dong, 1993; Forster et al., 1998; Currie & Dong, 2001; Zhou & Zhang, 2002; Makovicky & Norell, 2004; Makovicky, Apesteguía & Agnolín, 2005; Norell et al., 2009; Turner, Pol & Norell, 2011). However, they are not as hypertrophied as in other dromaeosaurids, such as *Velociraptor*, *Deinonychus*, *Microraptor* and *Sinornithosaurus*, in which they are extremely projected as ossified tendons and interconnected with those of the preceding chevrons (Ostrom, 1969; Norell & Makovicky, 1999; Perle, Norell & Clark, 1999; Xu, 2002).

#### Dorsal ribs

**Figure 6:**
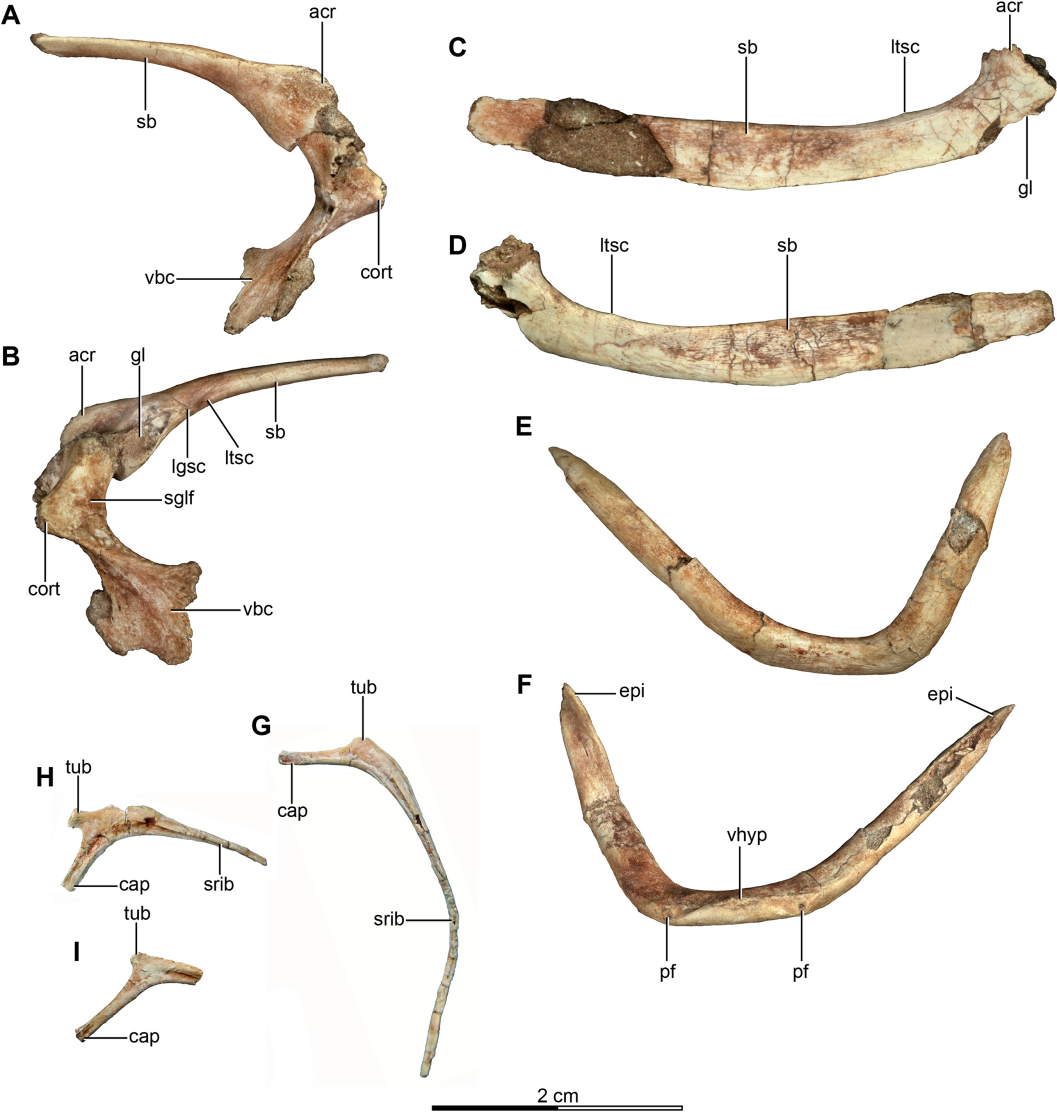
Pectoral girdle and dorsal ribs of the holotype of *Buitreraptor gonzalezorum* (MPCA 245). (A, B) Left scapula and coracoid, in (A) medial and (B) lateral view. (C, D) Right scapula, in (C) dorsal and (D) ventral view. (E, F) Furcula, in (E) anterior and (F) posterior view. (G–I) Dorsal ribs, in anterior view. acr, acromion; cap, capitulum; cort, coracoid tuber; epi, epicleidum; gl, glenoid cavity; lgsc, lateral groove of the scapula; ltsc, lateral tubercle of the scapula; pf, pneumatic foramen; sb, scapular blade; sglf, subglenoid fossa; srib, shaft of the rib; tub, tuberculum; vbc, ventral blade of the coracoid; vhyp, vestigial hypocleidum.

There are very few preserved dorsal ribs of *Buitreraptor*, represented by one almost complete rib and some fragmentary ones from the holotype specimen (Figs. 6G–6I). The more complete rib preserves the articular portion, including the tuberculum and the capitulum and most of the shaft (Fig. 6G). The tuberculum is very short with an elliptical and anteroposteriorly compressed articular surface. The capitulum is elongated with a slightly expanded end, and with a convex articular surface. The other fragmentary ribs generally preserve the proximal portion, and they differ mainly in the length of the capitulum and thus in its separation from the tuberculum (Figs. 6H and 6I). Based on the location of both articular processes, the distance between them, and the locations and development of the diapophyses and parapophyses on the dorsal vertebrae, it is possible to infer the position of the preserved ribs. Thus, those ribs with a larger distance between the tuberculum and the capitulum match the anterior dorsal vertebrae, because the latter have more elongated transverse processes and consequently the distance between their articular surfaces and the parapophyses is larger. The remaining ribs match better with middle or posterior dorsal vertebrae, also considering the comparative sizes of the ribs. The diapophyses and the parapophyses are located almost in a horizontal plane in the posterior dorsal vertebrae, with the parapophyses anteroventrally projected. Therfore, the tuberculum and the capitulum articulated with the vertebrae in an oblique, almost horizontal plane in the posterior section of the dorsal vertebral series. This differs from other dromaeosaurids such as *Deinonychus* (Ostrom, 1969), *Austroraptor* (Novas et al., 2009) and *U. comahuensis* (Novas & Puerta, 1997), and from troodontids (e.g., *Troodon*, Makovicky, 1995), and from avialans (e.g., *Archaeopteryx*, Elzanowski, 2002; *Patagopteryx*, Chiappe, 2002), where the parapohyses and diapophyses occupy a more vertical plane. On the other hand, in the dorsals of *Sinornithosaurus*, *Microraptor*, *Rahonavis* (Forster et al., 1998; Hwang et al., 2002; Xu, 2002) and the posterior dorsals of *Velociraptor* (Norell & Makovicky, 1999), the parapophyses are almost at the same level as the diapophyses, as in *Buitreraptor*. O’Connor & Claessens (2005) also noted that the costal articulations switched orientations from more vertical to more horizontal in the dorsal series of *Majungasaurus*, and interpreted this as an adaptation for increasing tidal volume in the abdominal air sacs.

The rib shaft of the most complete rib is bow-shaped, compressed in the proximal portion and decreasing in diameter distally and tapering to a point (Fig. 6G). An intercostal ridge is present on its anterior surface, and extends along the shaft, as is also observed on a dorsal rib of the specimen MPCN-PV-598. Fragments of three other rib shafts were preserved, joined together with matrix.

### Appendicular skeleton

#### Pectoral girdle

##### Scapula

Both scapulae are preserved in the holotype, although the left only preserves the proximal portion and proximal part of the scapular blade, whereas the right one preserves almost the entire scapular blade although the proximal portion is not well preserved (Figs. 6A–6D). The estimated total length of the scapula corresponds to approximately 70% of the humeral length (Table S1), a greater proportion than observed in some paravians such as *Sinornithosaurus* (64%), *Archaeopteryx* (60%; Ji et al., 1998), and *Anchiornis* (55%; Xu et al., 2008), but shorter than in *Deinonychus* (∼84%; Ostrom, 1969) and *Tianyuraptor* (88%; Zheng et al., 2010). The scapular blade is elongated and dorsoventrally compressed, with parallel dorsal and ventral edges, although it has a slightly transverse flare at mid-length, but then narrows slightly distally (Figs. 6C and 6D), a feature also observed in MPCN-PV-598 and which is likely autapomorphic for *Buitreraptor* (Novas et al., 2018). The blade is laterally arced so it has a bowed shape in dorsal and ventral view, similarly to the condition observed in *Rahonavis* (Forster et al., 1998) and *Velociraptor* (Norell & Makovicky, 1999). The blade is very thin distally, with sharp dorsal and ventral edges, and is very gently arced across its length in lateral view. However, the proximal part, which exhibits a ventromedial curvature, increases in thickness. The scapular blade of *U. comahuensis* also bows ventrally at its proximal end, but it is not medially curved as in *Buitreraptor* and it has a straight profile in dorsal view (Novas & Puerta, 1997). It is, however, more bowed in lateral view than the scapula of *Buitreraptor*. Near the ventral edge and close to the posterior rim of the glenoid fossa, there is short and shallow groove on the medial face of the scapula (Fig. 6B), which is also present in MPCN-PV-598 (Novas et al., 2018). Distal to this groove lies a small tubercle about 2 mm in length and triangular in dorsal and ventral view (Figs. 6B–6D), a structure also observed in MPCN-PV-598. Both the groove and the small prominence are interpreted as muscle insertion sites (Novas et al., 2018), possibly for the *M. subscapulare* (Jasinoski, Russell & Currie, 2006). Conversely, these features are not present on the scapulae of either *Unenlagia* (MCF PVPH 78) or *Rahonavis* (FMNH PR 2830).

The proximal portion of the scapula is mediolaterally thicker than the blade, especially at its contact with the coracoid. In lateral view, the ventral border expands as a protruding flange and constitutes the posterior and dorsal rims of the glenoid fossa (Fig. 6B). This fossa is mainly formed by the scapula with only a small section formed by the coracoid, and it faces mainly laterally though with a slight ventral component. The orientation of the fossa is similar to that observed in *Velociraptor*, *Tsaagan*, *Bambiraptor*, *Sinornithosaurus*, *Microraptor*, *Sinovenator*, *Gobivenator*, *Archaeopteryx*, *Confuciusornis* and *Jeholornis* (Ostrom, 1976a; Wellnhofer, 1974, 1992; Novas & Puerta, 1997; Chiappe et al., 1999; Norell & Makovicky, 1999; Paul, 2002; Xu, 2002; Zhou & Zhang, 2002, 2003a; Burnham, 2004; Norell et al., 2006; Novas, 2009; Tsuihiji et al., 2014). On the other hand, in other paravians such as *Deinonychus*, *Sinornithoides* and *Linhevenator* the fossa faces more posteroventrally (Ostrom, 1969; Currie & Dong, 2001; Xu et al., 2011). A deep and sharply defined pit interrupts the anterodorsal border of the glenoid fossa, and may mark the insertion of a glenohumeral ligament. Such a well-defined pit is absent in *Rahonavis*, although there is a small fossa extending from the rostral rim of the scapular glenoid that may be a homologous feature. The rim of the glenoid fossa is comparatively weakly raised when compared to that of other paravians, including *Rahonavis*, a condition that *Buitreraptor* shares with *Unenlagia* (see Novas et al., 2018).

The lateral surface of the scapula anterior to the glenoid fossa is concave ventral to the acromion process (Fig. 6B). This latter structure is transversely compressed and is triangular in lateral view. Its dorsal edge forms an almost continuous line with the dorsal edge of the scapula in lateral aspect, and is everted in dorsal view to overhang the scapulocoracoid suture as is typical for paravian taxa such as in *Unenlagia*, *Rahonavis*, *Sinornithosaurus*, *Microraptor* and *Archaeopteryx*, and also in troodontids as *Sinovenator* (Wellnhofer, 1992; Novas & Puerta, 1997; Xu, 2002; Novas, 2009). The tip of the acromion is incomplete, but judging from the taper of the preserved part, it probably had a pointed and anteroventrally directed apex as in other paravians. In particular, the general form and angle of the acromion process resemble the conditions observed in *Unenlagia*, but are different to those observed in *Rahonavis*, which has a thinner and much more anteriorly projected end that is lobate in dorsal view.

##### Coracoid

The coracoids of *Buitreraptor* are well-developed and have a “L”-shaped profile, with the proximal portion that articulates with the scapula set almost perpendicular to the ventral portion by a marked flexure that is level with the coracoid tuber (Figs. 6A and 6B). This morphology is similar to the coracoids of other paravians, such as *Velociraptor*, *Sinornithosaurus*, *Microraptor*, *Archaeopteryx* and *Confuciusornis* (Ostrom, 1975, 1976a; Wellnhofer, 1992; Chiappe et al., 1999; Norell & Makovicky, 1999; Elzanowski, 2002; Hwang et al., 2002; Xu, 2002), whereas in other coelurosaurs including alvarezsauroids (e.g., *Patagonykus*, Novas, 1997), ornithomimosaurs (Makovicky, Kobayashi & Currie, 2004), and some oviraptorosaurs (Balanoff & Norell, 2012; Makovicky & Sues, 1998) the coracoids are less inflected and form less of an angle or none at all. The proximal part of the coracoid dorsal to the flexure is missing its medial edge, but appears to have an anterior surface that is concave in rostral view. Laterally, this edge connects to the proximal terminus of the subglenoid fossa. The coracoid tuber is large and located anteroventral to the glenoid fossa (Fig. 6B). This tuber, which is homologous to the arcrocoracoid process of ornithurines, is triangular in lateral view, has a rounded tip and projects markedly anteriorly as in *Archaeopteryx*, *Sinornithosaurus* and *Microraptor* (Ostrom, 1975, 1976a; Xu, 2002). In other paravians this structure is lower and more anterolaterally projected, as in *Bambiraptor*, *Deinonychus*, *Velociraptor*, *Sinornithoides* and *Gobivenator* (Ostrom, 1969; Norell & Makovicky, 1999; Currie & Dong, 2001; Burnham, 2004; Tsuihiji et al., 2014). The subglenoid fossa has a smooth rim and is arcuate in lateral view. It descends vertically below the glenoid to the level of the coracoid tuber, and then curves posterolaterally from the apex of the coracoid tuber. It is widest anteroposteriorly where it forms the lateral surface of the coracoid tuber, but it tapers posteriorly toward the ventral edge as in *Sinornithosaurus* (Xu, 2002). In eudromaeosaurids such as *Velociraptor*, *Bambiraptor* and *Deinonychus* the subglenoid fossa is wider anteroposteriorly, more concave and more posterolaterally faced (Ostrom, 1969; Norell & Makovicky, 1999; Burnham, 2004).

The coracoid forms the anteroventral rim of the glenoid fossa, which is laterally projected as a marked flange, as in *Sinornithosaurus* (Xu, 2002). The coracoid foramen is not preserved in the holotype of *Buitreraptor*, and is likley lost to breakage, as the anteromedial part of both coracoids is broken. A similar break through the coracoid foramen in observed in a number of dromaeosaurids including the holotype of *Bambiraptor* (Burnham, 2004) and at least one specimen of *Velociraptor* (Norell & Makovicky, 1999). This foramen is clearly observed in some paravians (e.g., *Deinonychus*, *Sinornithosaurus*, *Microraptor*, *Sinornithoides*, *Jeholornis*), and another coelurosaurs as oviraptorosaurs (Makovicky & Sues, 1998), alvarezsauroids (Perle et al., 1994; Novas, 1997), and ornithomimosaurs (Osmólska, Roniewicz & Barsbold, 1972; Makovicky et al., 2010).

The ventral expanse of the coracoid below the flexure forms a large, thin sheet of bone that is posteroventrally directed. This sheet has an approximately quadrangular form in ventral view, although the ventral edge is broken. Both the internal and external surfaces are smooth and flat. The lateral border of the ventral section of the coracoid is concave in anterior view and bears a posterolateral process as in *Sinornithosaurus* (Xu, 2002). The anterior surface of the lamina is almost perpendicular to the surface of the subglenoid fossa. The medial border is broken in both coracoids of the holotype but apparently was concave in anterior view and probably would have been continuous with the medial border of the proximal portion of the bone dorsal to the flexure. The angle formed between the proximal portion of the coracoid and the ventral lamina is very prominent, with the axis connecting the articular border for the scapula and the coracoid tubercle set at approximately 90° with respect to the dorsoventral axis that extends through the ventral lamina. This morphology is similar to the coracoids of *Sinornithosaurus*, *Microraptor*, *Anchiornis*, *Archaeopteryx* and *Sapeornis* (Wellnhofer, 1992; Xu, 2002; Zhou & Zhang, 2003b; Mayr et al., 2007; Hu et al., 2009), whereas in other paravians such as *Velociraptor*, *Bambiraptor*, *Deinonychus*, *Sinornithoides* and *Gobivenator* the angle between the proximal and distal portions of the coracoid is greater than 90° (Ostrom, 1969; Norell & Makovicky, 1999; Currie & Dong, 2001; Burnham, 2004; Tsuihiji et al., 2014). The angle formed between the ventral lamina of the coracoid and the scapular blade in lateral view is less than 90° in *Buitreraptor* (Figs. 6A and 6B), similar to the angle observed in *Sinornithosaurus*, but this angle is greater in *Microraptor* and *Confuciusornis* (105° and 95° respectively; Hwang et al., 2002; Xu, 2002).

##### Furcula

The furcula of *Buitreraptor* is large and approximately “U”-shaped (Figs. 6E and 6F), as in *Sinornithosaurus*, *Archaeopteryx* and *Confuciusornis* (Ostrom, 1976a; Chiappe et al., 1999; Xu, 2002), rather than “V”-shaped as in *Velociraptor* (Norell & Makovicky, 1999). The bone is formed by two robust, laterodorsally directed and posteriorly curved rami, joined together at a gently curved angle. The rami are set at an angle close to 90° to each other, as measured between chords taken from the midline and through each omal tip. This angle is more acute than that observed in *Velociraptor* (Norell & Makovicky, 1999), but is very similar to those observed in *Microraptor*, *Sinornithosaurus*, and in basal avialans such as *Confuciusornis* (Chiappe et al., 1999; Hwang et al., 2002; Xu, 2002). However, the angle may be slightly affected by taphonomic deformation as revealed by the different inclinations of the rami. In fact, in MPCN-PV-598 the furcula is undeformed and shows a more acute angle between its rami. Each ramus is elliptical in cross section at its base, but gradually tapers distally as it curves posteriorly. Each omal tip is twisted so that the medial border is inclined lateroventrally (Fig. 6F). The right ramus has its posterior surface broken revealing the internal anatomy, including trabeculae fused to the thin wall of the bone and spanning the hollow interior, supporting the inference that the furcula was internally pneumatic.

The midline of the furcula has a low and short ridge on the posteroventral surface, which likely represents a vestigial hypocleidium (Fig. 6F). The absence of a prominent hypocleidium also characterizes other basal paravians such as *Sinornithosaurus*, *Microraptor*, and *Confuciusornis* (Chiappe et al., 1999; Hwang et al., 2002; Xu, 2002). On the posterior surface, a small circular foramen flanks each end of the midline ridge. This pair of foramina likely represents pneumatic connections into the interior (Fig. 6F) communicating with the internal trabecular structure of the bone. In *Bambiraptor*, a small foramen is also observed on each ramus of the furcula (Burnham et al., 2000), a condition similar to that of *Buitreraptor*, although in other dromaeosaurids, such as *Velociraptor*, there is no evidence for pneumatic openings in the furcula (Norell & Makovicky, 1999).

#### Forelimb

##### Humerus

**Figure 7:**
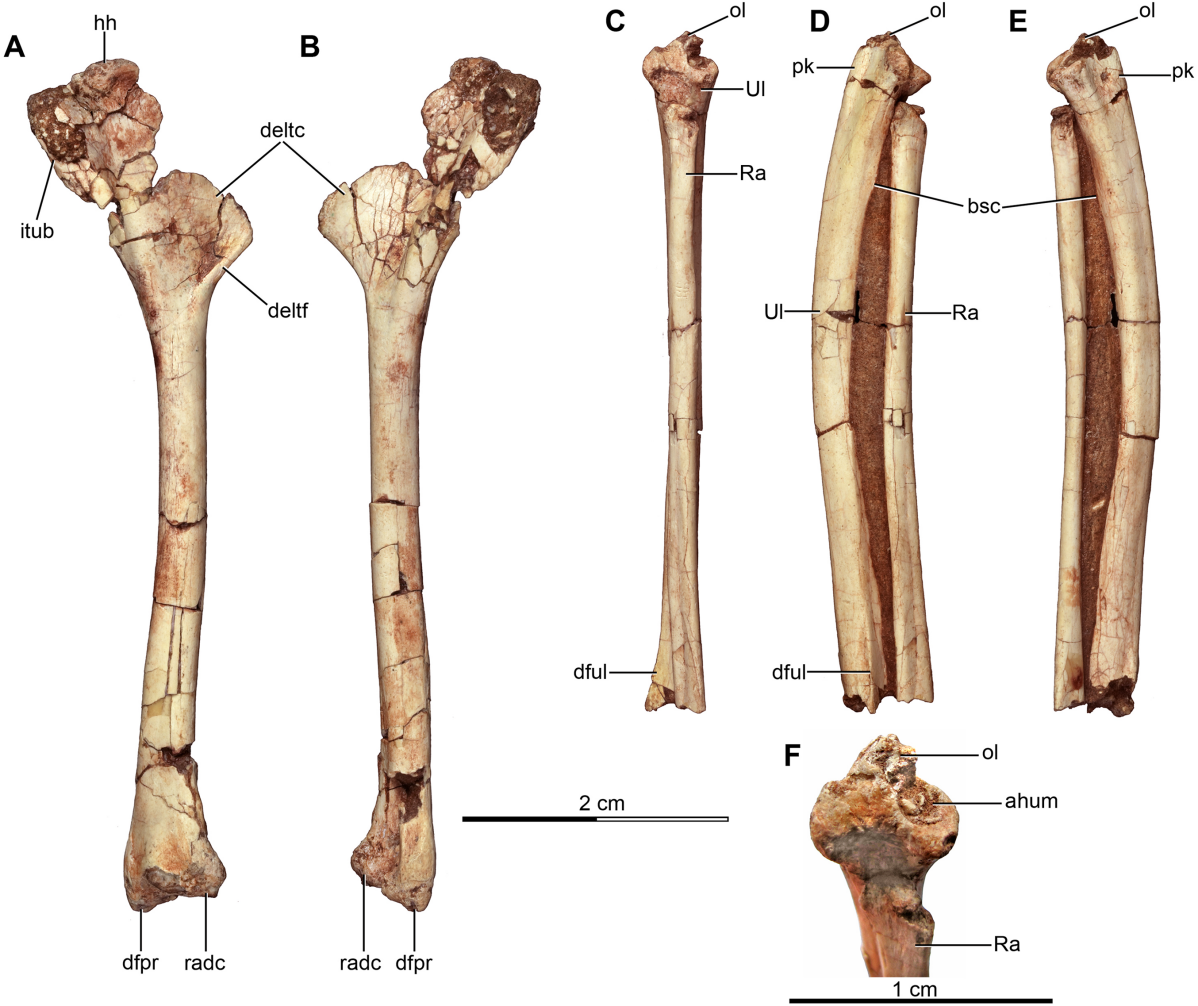
Forelimb of the holotype of *Buitreraptor gonzalezorum* (MPCA 245). (A, B) Right humerus, in (A) lateral and (B) medial view. (C–E) Right ulna and radius, in (C) anterior, (D) lateral (E) and medial view. (F) Detail of the proximal articular surface of the right ulna, in proximal view. Scales: 2 cm for A–E, 1 cm for F. ahum, articular surface for the humerus; bsc, bicipital scar; deltc, deltopectoral crest; deltf, flange on the lateral surface of the deltopectoral crest; dfpr, distal flexor process; dful, distal flange of the ulna; hh, humeral head; itub, internal tuberosity; ol, olecranon; pk, posterior keel; Ra, radius; radc, radial condyle; Ul, ulna.

The humerus is elongated and slender (Figs. 7A and 7B), with a length that represents about 93% of the femoral length (Table S1). This proportion is similar to that observed in some dromaeosaurids such as *Microraptor*, *Sinornithosaurus* and *Bambiraptor* (Hwang et al., 2002; Xu, 2002; Burnham, 2004), and is similar (or exceeded) in basal avialans (Makovicky, Apesteguía & Agnolín, 2005). On the other hand, the humerus of *Austroraptor* is considerably shorter, representing only 47% of the femoral length (Novas et al., 2009), and thus differs significantly from other dromaeosaurids and other paravians. However, the shortening of the humerus is also observed in some other dromaeosaurids like *Mahakala*, *Tianyuraptor* and *Zhenyuanlong* (Zheng et al., 2010; Turner, Pol & Norell, 2011; Lü & Brusatte, 2015), and in troodontids such as *Linhevenator* (Xu et al., 2011).

The proximal part of the humerus has a well-developed and triangular deltopectoral crest, projecting anterolaterally as in *Unenlagia*, but differing from the anteriorly projected crest of *Austroraptor* and other dromaeosaurids such as *Bambiraptor* (Burnham, 2004; Novas et al., 2009). In other dromaeosaurids including *Sinornithosaurus* and *Microraptor* the crest has a more trapezoidal shape, without a pointed apex (Xu, 2002). The ventral border merges with the shaft at an angle of approximately 140°, similar to the angle observed in *U. comahuensis* and *Austroraptor* (Novas & Puerta, 1997; Novas et al., 2009). The medial surface of the crest is smooth and the contact zone between this and the anterior surface of the shaft is markedly concave (Fig. 7B). The lateral surface of the crest is smooth along most of its surface but is traversed by a shallow, well-defined scar paralleling the ventral border, and extending from a point near the the diaphysis to the apex of the deltopectoral crest (Fig. 7A), a feature also present in MPCN-PV-598. The lateral surface of the deltopectoral crest has been interpreted as the insertion of several shoulder muscles including a large insertion for the *M. deltoideus scapularis* and a smaller one for the *M. latissimus dorsi* (Jasinoski, Russell & Currie, 2006) and it is likely the scar corresponds to one of these insertions. A similar scar is present in *Deinonychus*, *U. comahuensis* and troodontids like *Linhevenator* (Ostrom, 1969; Novas & Puerta, 1997; Xu et al., 2011) and also some other pennaraptorans like *Citipati* (IGM 100), but conversely this trait is not observed in *Austroraptor* and *U. paynemili* (Calvo, Porfiri & Kellner, 2004; Novas et al., 2009), although the poor preservation of the surface of the crest in *U. paynemili* renders this observation uncertain.

A well-developed internal tuberosity that is longitudinally expanded and rectangular projects posteriorly from the proximal end of the humerus. The depth of this tuberosity is approximately half the length of the deltopectoral crest. In size and shape, it resembles those of *U. comahuensis*, *U. paynemili* and *Austroraptor* (Novas & Puerta, 1997; Calvo, Porfiri & Kellner, 2004; Novas et al., 2009), and also is similar to the deltopectoral crest of *Archaeopteryx* (Rauhut, 2003). Conversely in other coelurosaurs like oviraptorosaurs (Osmólska, Currie & Barsbold, 2004) and ornithomimosaurs (Osmólska, Roniewicz & Barsbold, 1972; Makovicky et al., 2010), the internal tuberosity is shorter and more rounded in lateral aspect. The proximal articular head is roughly elliptical in end view, with its main axis directed anteroposteriorly and is slightly projected laterally from the shaft.

The humerus is gently sigmoid in lateral view with the proximal end curving posterolaterally, while the distal end is slightly curved anterolaterally. This shape is similar to the humerus of *U. comahuensis* and *Deinonychus* (Ostrom, 1969; Novas & Puerta, 1997), whereas in *U. paynemili* the proximal portion is posterolaterally curved but the rest of the diaphysis remains straight (Calvo, Porfiri & Kellner, 2004). The humerus of the holotype of *Austroraptor* is roughly straight for much of its length, although the distal portion is anteriorly curved (Novas et al., 2009). In contrast, the humerus of the referred specimen MML 220 has a sigmoid curvature (Currie & Paulina Carabajal, 2012). In the humerus of *Buitreraptor*, the distal end of the shaft is expanded predominantly in the anteroposterior plane but is transversely compressed. The shaft is distally twisted such that the axis of the distal articulation is anterolaterally–posteromedially directed similar to the humeri of *Austroraptor*, *U. paynemili*, *Sinornithosaurus*, *Microraptor* and *Deinonychus* (Ostrom, 1969; Xu, 2002; Calvo, Porfiri & Kellner, 2004; Novas et al., 2009). The distal end of the humerus has a pronounced anterior curvature in *Austroraptor*, both in the holotype and in the specimen MML 220 (Novas et al., 2009; Currie & Paulina Carabajal, 2012), a character not present in either unenlagiines or other dromaeosaurids. Both the ulnar and the radial condyles are slightly anteromedially projected and are separated by a shallow groove on the humerus of *Buitreraptor*. The radial condyle is generally rounded and scarcely projected distally. Conversely, the ulnar condyle bears a conical flexor process that projects distally as a pointed tip (Figs. 7A and 7B). The distal condyles are less developed in *U. paynemili*, and in *Austroraptor* the ulnar condyle is smaller and does not bear a flexor process (Currie & Paulina Carabajal, 2012). Moreover, the radial condyle of *Austroraptor* is anteriorly projected, a condition not present in *Buitreraptor*. It is noteworthy, that isolated avialan humeri from the Maevarano Formation of Madagascar that have tentatively been referred to *Rahonavis* also exhibit a pointed and distally projected flexor process adjacent to the ulnar condyle (O’Connor & Forster, 2010), a feature also observed in some enantiornithine birds. In the Malagasy specimens, the olecranon fossa appears to separate the ulnar condyle from the flexor process in caudal views (O’Connor & Forster, 2010), whereas the flexor fossa is less distinct and open caudally in *Buitreraptor*. Another difference between these taxa, and indeed between *Buitreraptor* and other basal paravians is the presence of a low tubercle or mound on the posterior surface of the distal portion of the humerus, which thus has a subtriangular profile in distal view. This posterior tubercle is not observed in the humeri referred to *Rahonavis* (O’Connor & Forster, 2010), nor other basal paravians such as *Deinonychus* (MCZ 4371).

##### Ulna

Only the right ulna of the holotype was preserved, and it is almost complete missing only the distal articulation (Figs. 7C–7F). The shaft is bowed and posteriorly convex, leaving a wide space between the ulna and the radius, a feature also observed in *Rahonavis* and diagnostic of Maniraptora (Gauthier, 1986), including *Microvenator*, *Sinornithoides*, *Troodon*, *Deinonychus*, *Sinornithosaurus*, *Microraptor*, *Mahakala* and *Archaeopteryx* (Ostrom, 1969; Wellnhofer, 1974; Makovicky & Sues, 1998; Currie & Dong, 2001; Hwang et al., 2002; Xu, 2002; Turner et al., 2007; Turner, Pol & Norell, 2011). The ulna of *Buitreraptor* has a subtriangular cross section, which is more conspicuous at the proximal and distal sections of the shaft, and the posterior surface bears a marked longitudinal ridge as in the ulna of *Mahakala*, *Velociraptor* and *Bambiraptor* (Norell & Makovicky, 1999; Burnham, 2004; Turner, Pol & Norell, 2011). In *Rahonavis* this posterior ridge is also present although less prominent.

The proximal articulation is divided in two by an anteriorly widening groove (Fig. 7F). The lateral articular surface is convex whereas the medial one is concave for articulation with the pointed ulnar condyle of the humerus. This morphology of two distinct proximal articular surfaces is also observed on the ulnae of other paravians, such as *Deinonychus*, *Velociraptor*, *Bambiraptor*, *Confuciusornis* and *Patagopteryx* (Ostrom, 1969; Chiappe et al., 1999; Norell & Makovicky, 1999; Chiappe, 2002; Burnham, 2004). The olecranon process is located posterior to the midline between the articular facets, and is triangular in shape in proximal view and modest in size, as is common in many coelurosaurs (Rauhut, 2003). The morphology of the articular surfaces and the olecranon confer a triangular outline to the proximal portion of the bone, similar to the outlines in *Rahonavis* and other dromaeosaurids including *Deinonychus*, *Velociraptor*, *Pyroraptor* and *Microraptor* (Ostrom, 1969; Norell & Makovicky, 1999; Allain & Taquet, 2000; Hwang et al., 2002). The olecranon is distally continuous with the ridge along the posterior border of the shaft, which is more pronounced along the proximal half of the shaft but grades into the shaft surface distally (Figs. 7D and 7E).

The shaft is slightly transversely compressed. A shallow bicipital scar is located on the anterior surface of the proximal part just below the ulnar articular facet, and formed as a short and shallow groove bordered caudally by a low ridge that can be observed in lateral and medial views (Figs. 7D and 7E). A similar shallow bicipital scar can be observed in *Rahonavis*, *Austroraptor* (MML 220) and other paravians such as *Deinonychus* (although in this taxon it is longer), *Velociraptor* and *Mahakala* (Ostrom, 1969; Norell & Makovicky, 1999; Turner, Pol & Norell, 2011), whereas in some avialans it is much more pronounced, as in *Confuciusornis*, *Yanornis*, *Yixianornis* and *Apsaravis* (Chiappe et al., 1999; Zhou & Zhang, 2001; Clarke & Norell, 2002). In *Rahonavis* (FMNH PR 2830), the scar faces more anteriorly than medially in contrast to *Buitreraptor*.

Distally the posterior border of the shaft turns more rounded, whereas the anterolateral border projects laterally forming a triangular ridge with a sharp edge bordering a flat anterior surface on the distal end of the ulna where it braces the distal end of the radius (Figs. 7C and 7D). *Rahonavis* bears a small, rounded tubercle in the homologous spot, but the anterior face of the ulna adjacent to it is gently rounded rather than flat (Forster et al., 1998). The posteromedial surface of the distal part of the ulna bears the base of a low, longitudinal tubercle or ridge, which is broken together with almost the entire distal articulation. A similar posteromedial protuberance is observed in *Rahonavis*, *Deinonychus* and other paravians (Forster et al., 1998; Ostrom, 1969; FMNH PA 344). Unlike *Rahonavis* (Forster et al., 1998) and at least one specimen of *Velociraptor* (IGM 100/981; Turner, Makovicky & Norell, 2007), the ulna of *Buitreraptor* does not exhibit feather quill knobs.

##### Radius

Only the right radius of the holotype is preserved in articulation with the ulna (Figs. 7C–7F). It has an almost straight shaft with just a slight posterior curvature. The radial shaft of the referred specimen MPCN-PV-598 is markedly anteriorly bowed, although this shape is probably a taphonomic artifact, taking into account the better preservation of the holotype. In the holotype, the radius is comparatively slimmer than the ulna, with the widest part of the shaft approximately half the width of the ulnar shaft, as in *Austroraptor* (specimen MML 220) and *Rahonavis* (Forster et al., 1998). A radial shaft that is markedly (<65%) slimmer than the ulnar shaft is also observed in other paravians including *Microraptor* and *Confuciusornis* (Hwang et al., 2002; Chiappe et al., 1999), but not in other taxa including *Archaeopteryx* (Mayr et al., 2007) and also not in *Anchiornis* (Pei et al., 2017). The proximal end expands slightly toward the elbow articulation. The proximal articulation is poorly preserved, but it shows a subtriangular shape in proximal view (Fig. 7F), as is also observed in the radii of *Neuquenraptor*, *Rahonavis*, *Saurornitholestes*, *Deinonychus* and *Bambiraptor* (Ostrom, 1969; Forster et al., 1998; Burnham, 2004; Novas & Pol, 2005). However, the proximal surface of the radius of MPCN-PV-598 has an elliptical form. A small tuber is observed on the lateral surface of the shaft just below the proximal articulation, and continue for a short distance along the shaft as a shallow ridge. This tuber may represent a point of attachment for either the *M. brachialis* or the *M. biceps* (Burch, 2014) and is also observed in the radius of *Rahonavis* (FMNH PR 2830). The shaft is slightly flattened distally along its anterolateral surface, but widens slightly along the posterolateral–anteromedial axis. The anteromedial border of the shaft is narrow and sharp near the distal end, which unfortunately is not preserved.

##### Manual bones

**Figure 8:**
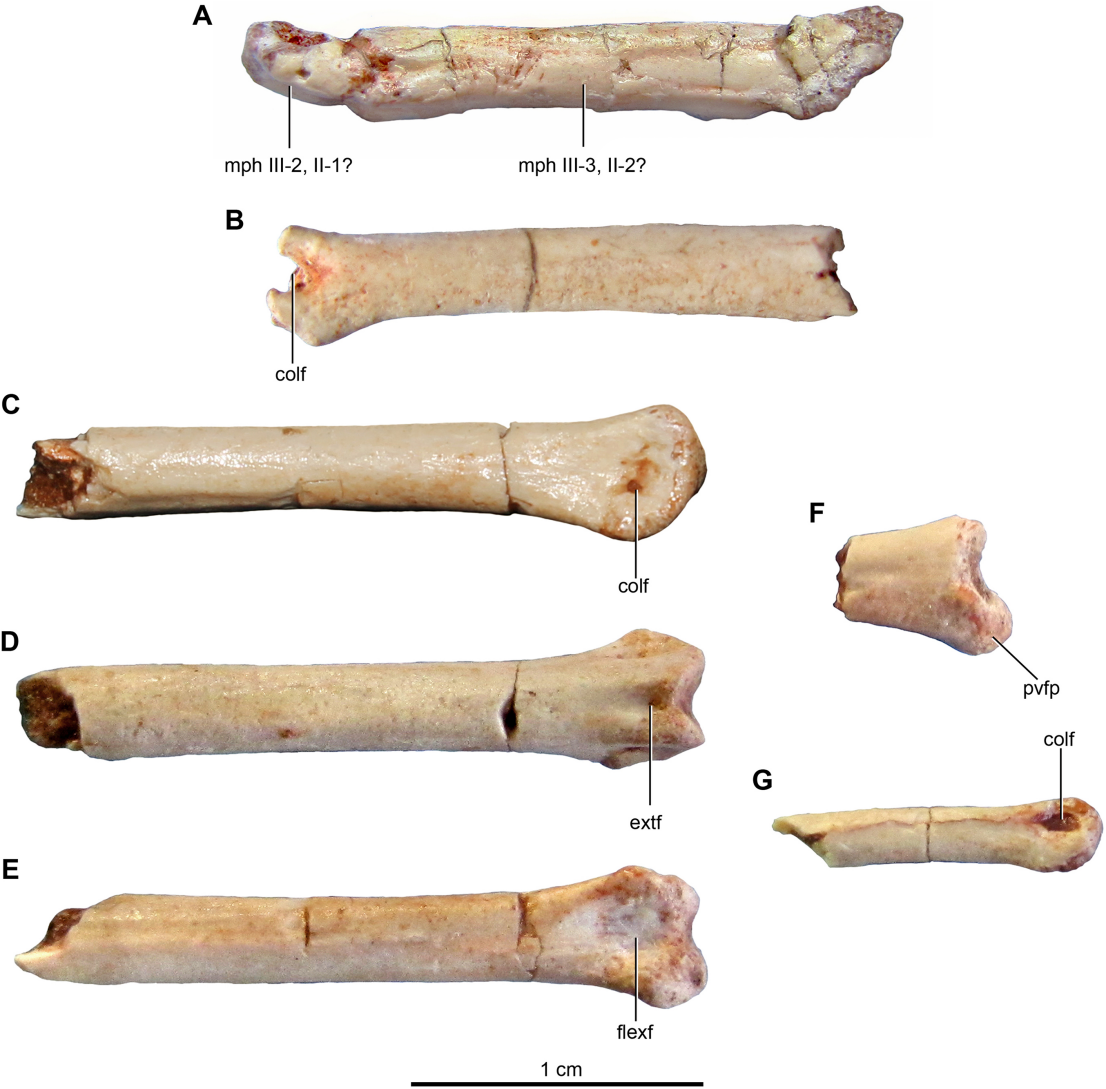
Incomplete manual bones of the holotype (MPCA 245) and referred specimen (MPCA 471-C) of *Buitreraptor gonzalezorum*. (A) Possible phalanges III-2 and III-3 or II-1 and II-2 of MPCA 245. (B) Possible metacarpal III or phalanx II-1 of MPCA 245, in lateral or medial view (indeterminate). (C–E) Possible metacarpal III or phalanx II-1 of MPCA 471-C, in (C) side, (D) dorsal and (E) ventral view. (F) Possible proximal end of phalanx III-2 or III-3 of MPCA 471-C, in side view. (G) Incomplete pre-ungual phalanx of MPCA 471-C, in side view. colf, fossa for the collateral ligament; extf, extensor fossa; flexf, flexor fossa; mph, manual phalanx; pvfp, proximoventral flexor process.

The holotype preserves two manual elements, which are incomplete and therefore difficult to identify. Information provided by the specimen MPCN-PV-598, which has a nearly complete and articulated hand (Agnolín & Novas, 2013; Novas et al., 2018) allows for comparisons to identify these elements. One of the manual elements of the holotype is articulated with the fragment of another bone (Fig. 8A). It has a straight shaft that is subtriangular in cross section and is slightly expanded at either end, although the distal end is incomplete so the total length is unknown. It differs from metacarpals I and II of MPCN-PV-598, in which metacarpal I is markedly short and transversely expanded and metacarpal II is relatively robust and transversely expanded (Novas et al., 2018). It also differs from phalanges III-1 and III-2, which are very short as in other dromaeosaurids (Gauthier, 1986; Norell & Makovicky, 2004). This fragment may correspond to the proximal portion of phalanx II-2 or III-3 as the shape of articulation between the bones resembles that between phalanges III-2 and III-3, or between phalanges II-2 and II-1 of the hand of MPCN-PV-598. However, the proximoventral flexor tubercle observed on phalanx III-3 of MPCN-PV-598 is not observed on this fragment of the holotype, although that could be due to poor preservation.

The other manual bone preserved in the holotype has an elongated and cylindrical shaft and a well-developed distal ginglymoid articulation (Fig. 8B). The proximal portion was not preserved. The shaft is straight and has an elliptical cross section. A flexor fossa is observed on the distal portion, which also bears two well-developed collateral ligament pits. Its morphology does not resemble the short and robust metacarpal I, the transversely expanded metacarpal II, or the markedly short phalanges III-1 and III-2 of MPCN-PV-598. Neither does it resemble any of the penultimate phalanges, which have dorsally displaced collateral ligament pits. Thus, this bone possibly corresponds to metacarpal III or to phalanx II-1.

The specimens MPCA 471-A, MPCA 471-B and MPCA 471-C include several fragmentary manual bones, but most of them are incomplete and their indentity is therefore indeterminate (Figs. 8C–8G). One of these elements found with MPCA 471-C preserves part of the shaft and the distal articulation (Figs. 8C–8E). The shaft is mainly straight and with a roughly elliptical cross section. The distal condyles are well-developed and separated by a conspicuous groove. A flexor fossa is observed ventrally and an extensor fossa is present dorsally and delimited by the raised dorsal parts of the condyles. Shallow and round collateral ligament pits are located on the lateral and medial sides. This element also differs from metacarpals I and II, and also from phalanges III-1 and III-2 of MPCN-PV-598, so it likely corresponds to metacarpal III or possibly phalanx II-1.

The referred specimen MPCA 471-C also includes several fragments that are likely parts of manual phalanges. One of them bears a proximal expansion that resembles the proximoventral flexor processes observed either phalanges III-2 or III-3 of MPCN-PV-598 (Fig. 8F). Another fragmentary bone corresponds to the distal half of a penultimate phalanx as it has collateral ligament pits that are dorsally displaced relative to the center of the distal condyles when viewed laterally or medially (Fig. 8G), as can be observed in the hand of MPCN-PV-598. Another fragment from MPCA 471-C also has dorsally located collateral ligament pits, so it probably corresponds to another penultimate phalanx, though it is a bit larger than the previously described element.

#### Pelvic girdle

##### Ilium

**Figure 9:**
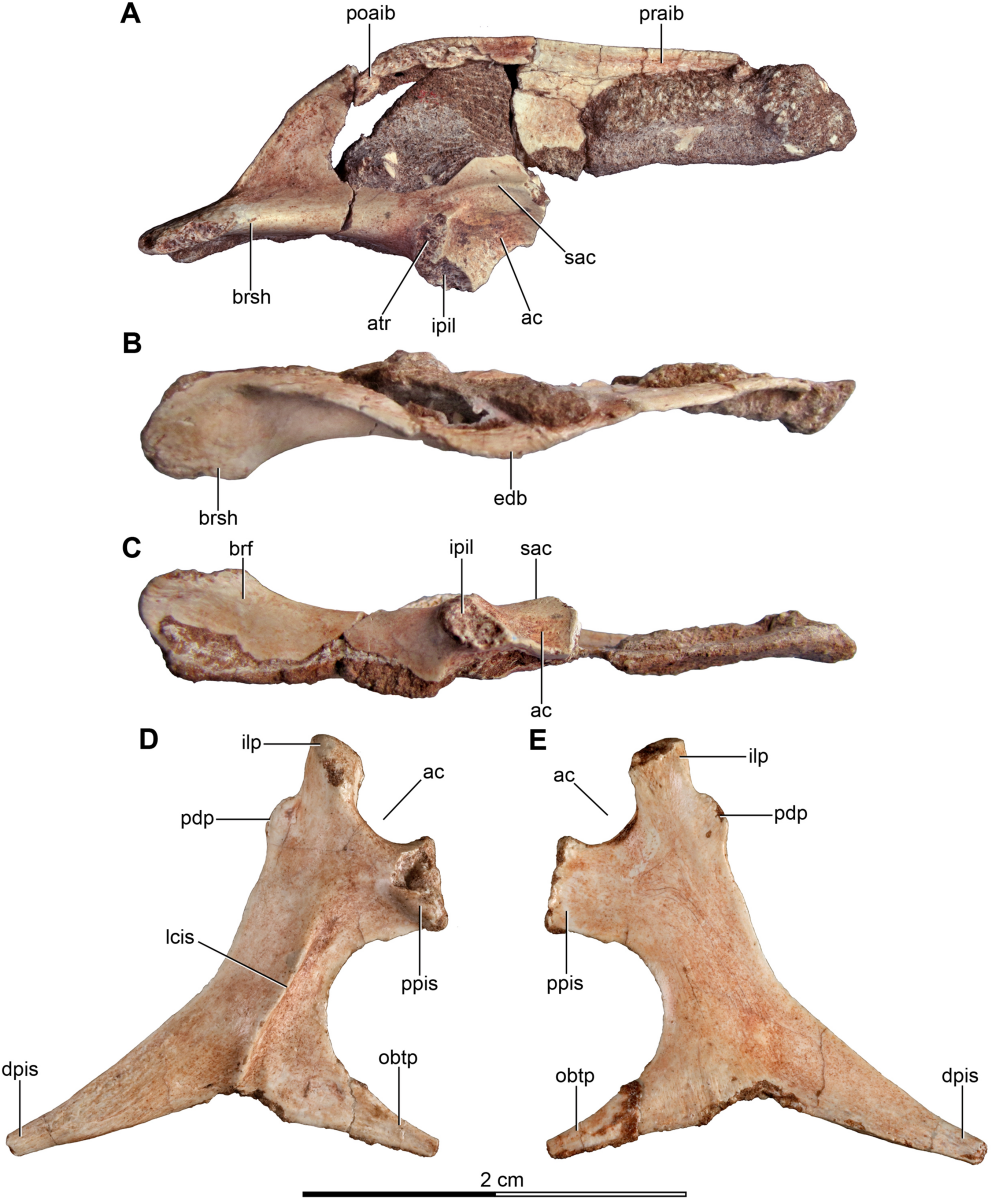
Pelvic bones of the holotype of *Buitreraptor gonzalezorum* (MPCA 245). (A–C) Right ilium, in (A) lateral, (B) dorsal and (C) ventral views. (D, E) Right ischium, in (D) lateral and (E) medial view. ac, acetabulum; atr, antitrochanter; brf, brevis fossa; brsh, brevis shelf; dpis, distal process of the ischium; edb, everted dorsal border of the ilium; ilp, iliac peduncle of the ischium; ipil, ischiadic peduncle of the ilium; lcis, lateral crest of the ischium; obtp, obturator process; pdp, posterodorsal process; poaib, postacetabular iliac blade; ppis, pubic peduncle of the ischium; praib, preacetabular iliac blade; sac, supracetabular crest.

Part of both the right and left ilia are preserved in the holotype. The right is missing most of the preacetabular region (Fig. 9), whereas the left only preserves a heavily eroded postacetabular blade. The referred specimen MPCA 238 has a better preserved right ilium articulated with the sacral vertebrae, which includes the postacetabular portion and the pubic peduncle articulated with the pubis (Fig. 5).

Although the ilium is incomplete in all specimens, we estimate its total length to be approximately 50% of the femoral length (Table S1) (57% in MPCN-PV-598, Novas et al., 2018). This proportion is similar to *Unenlagia* (53%) and *Microraptor* (Hwang et al., 2002), but shorter than in *Sinornithosaurus* (Xu, 2002) or *Velociraptor* (Norell & Makovicky, 1999). An ilium with a length that represents less than 60% of the femoral length is a condition observed not only in basal dromaeosaurids, but also in other paravians such as *Sinovenator* and *Archaeopteryx* (Xu, 2002; Xu et al., 2002). Conversely, in most non-avian theropods the ilium exceeds 70% of the length of the femur (Xu, Zhou & Wang, 2000; Xu, 2002). An exception among non-avialan paravians is the ilium of *Rahonavis*, which exceeds 80% of the femoral length. The right ilium of the holotype of *Buitreraptor* reveals that the preacetabular blade was longer than the postacetabular blade, although the full length is unknown (Fig. 9A). This trait can be corroborated in the more complete ilia of MPCN-PV-598, and is also observed in *U. comahuensis*, *Rahonavis*, *Tianyuraptor*, *Bambiraptor* and *Saurornitholestes* (Novas & Puerta, 1997; Forster et al., 1998; Burnham et al., 2000; Burnham, 2004; Zheng et al., 2010; Turner, Makovicky & Norell, 2012), as well as in basal avialans such as *Archaeopteryx*, *Jeholornis* and *Confuciusornis* (Wellnhofer, 1974; Ostrom, 1976a; Chiappe et al., 1999; Zhou & Zhang, 2002). In other dromaeosaurids including *Mahakala*, *Microraptor*, *Velociraptor*, *Adasaurus* and *Achillobator*, the preacetabular blade is comparable in length to the postacetabular blade (Norell & Makovicky, 1997, 2004; Perle, Norell & Clark, 1999; Hwang et al., 2002; Xu, 2002; Turner et al., 2007; Turner, Pol & Norell, 2011; Turner, Makovicky & Norell, 2012). Unfortunately, the edges of the preacetabular blade are not preserved in any of the specimens, so the exact shape of the anterior border remains unknown and we cannot confirm the presence of an anteroventral process similar to that observed in *U. comahuensis* (Novas & Puerta, 1997).

A remarkable feature of the ilium of *Buitreraptor* is the strong lateral eversion of the dorsal border, which is most pronounced at the level of the ischiadic peduncle and the acetabulum. This trait is observed both in the holotype and in MPCA 238 (Figs. 5 and 9B), but is not present in either *U. comahuensis* or *U. paynemili*, and the ilium of *Rahonavis* exhibits a much weaker degree of eversion of the dorsal rim (FMNH PR 2830). With the pubic and ischiadic peduncles oriented vertically and the acetabulum facing laterally, the dorsal border of the ilium is almost horizontally oriented at the level of the ischidiac peduncle as is observed in MPCA 238. Eversion of the dorsal iliac border is observed in *Mahakala* (Turner, Pol & Norell, 2011), whereas in other paravians, such as *Sinovenator* and *Mei*, the postacetabular blade is only gently laterally curved (Xu, 2002; Gao et al., 2012). However, in *Buitreraptor* the degree of eversion is more pronounced along the dorsal border and likely autapomorphic. MPCA 238 also demonstrates that the pelvis widens posteriorly, with the dorsal borders of both ilia increasingly farther apart caudally (Fig. 5D). Posteriorly diverging iliac blades are also observed in several other paravians including *Velociraptor*, *Sinovenator* and *Jeholornis* (Norell & Makovicky, 1997; Xu et al., 2002; Zhou & Zhang, 2003a).

The lateral surface of the ilium of MPCA 238 bears a low flexure extending from the dorsal rim of the acetabulum toward the posterodorsal border. The medial surface of this ilium is concave opposite the flexure zone, demonstrating that this convexity is not formed by a ridge of bone, but rather is due to the curvature of the iliac blade, and thus differs from the lateral crest observed in tyrannosauroids (e.g., *Stokesosaurus*, Madsen, 1974; *Tyrannosaurus rex*, Brochu, 2003). A similar flexure is also present in the ilia of *U. comahuensis* and *U. paynemili*. In *Rahonavis*, a subtle convexity is present spanning the height of the iliac blade from the acetabular rim to the supratrochanteric process, but it is not matched by a concavity on the medial surface of the ilium. Carrano & Hutchinson (2002) suggested this convexity seperates the *M. iliofemoralis* group from the *M. iliofibularis* muscles group in birds and possibly non-avian theropods. If this is correct, then the iliofemoralis group muscles would have originated from a larger area on the ilium than the iliofibularis muscles in unenlagines, an asymmetrical condition also observed in extant birds (Carrano & Hutchinson, 2002). A discrete supratrochanteric process similar to that observed in *U. comahuensis* is not present in *Buitreraptor*, although this region does correspond to the maximally everted section of the dorsal rim of the ilium.

The acetabulum is large, and the right ilium of the holotype demonstrates that the medial rim is ventrally expanded, so that the acetabular opening is partially closed medially (Fig. 9A), as also occurs in the specimen MPCN-PV-598. This trait is also observed in *U. comahuensis*, *Archaeopteryx* and other Cretaceous avialans such as *Patagopteryx* and the hesperornithiforms (Martin & Tate, 1976; Martin, 1983; Bonaparte, 1991; Alvarenga & Bonaparte, 1992; Chiappe, 2002; Novas, 2004), and has been considered a shared derived trait of avialans (Novas, 2004). In MPCA 238, the supracetabular crest is low but it extends far anteroventrally forming the posterior rim of the pubic peduncle and it reaches the articulation for the pubis (Figs. 5A and 4E), a feature also observed in MPCN-PV-598 and in *U. comahuensis* (Novas & Puerta, 1997). In other dromaeosaurids the crest is much more reduced or absent, as in *Mahakala* (Turner, Pol & Norell, 2011), in microraptorines (Hwang et al., 2002), and more derived taxa. Posteriorly, the crest is diminished and it terminates anterior to the ischiadic peduncle.

The pubic peduncle was preserved in MPCA 238, and is anteroposteriorly wide as in *U. comahuensis*, although it is not complete (Figs. 5A, 5C and 5E). In MPCN-PV-598, this peduncle is almost complete, and it is as anteroposteriorly wide as the acetabulum (Novas et al., 2018). In both MPCA 238 and MPCN-PV-598, the pubic peduncle projects farther ventrally than the ischiadic peduncle, a common trait among deinonychosaurs (Gauthier, 1986), as observed in *Deinonychus* and *Rahonavis*, and the basal avialans *Archaeopteryx* and *Jeholornis* (Ostrom, 1969; Wellnhofer, 1974; Forster et al., 1998; Zhou & Zhang, 2002, 2003a). The ischiadic peduncle is both shallower and narrower in lateral view than the pubic peduncle. The antitrochanter, which is small and forms a roughly conical projection, is located on the posterolateral rim of the ischiadic peduncle (Figs. 5E and 9A).

The brevis shelf is lobate and projects both posteriorly and laterally beyond the vertical part of the iliac blade (Makovicky, Apesteguía & Agnolín, 2005) (Figs. 5E and 9A). This projection of the brevis shelf imbues the dorsal border of the postacetabular blade with a dorsally concave curvature in lateral view, a trait also observed also in *U. comahuensis*, *U. paynemili* and *Rahonavis* (Novas & Puerta, 1997; Forster et al., 1998; Calvo, Porfiri & Kellner, 2004; Novas, 2004). Novas (2004) noted that variation in this trait is observed across the known specimens of *Archaeopteryx*. Among dromaeosaurids some taxa present a convex border, as *Velociraptor*, *Bambiraptor*, *Deinonychus*, *Tianyuraptor* and *Mahakala* (Ostrom, 1969; Norell & Makovicky, 1997, 1999; Burnham et al., 2000; Burnham, 2004; Turner et al., 2007; Turner, Pol & Norell, 2011; Zheng et al., 2010), whereas others have a tapering border, as in *Microraptor* and *Hesperonychus* (Hwang et al., 2002; Xu, 2002; Longrich & Currie, 2009). In troodontids such as *Sinovenator* and *Anchiornis* (Xu, 2002; Hu et al., 2009), and in basal avialans such as *Jeholornis* and *Confuciusornis* (Chiappe et al., 1999; Zhou & Zhang, 2002), the dorsal border of the postcetabular blade is convex.

The brevis shelf is wide and projects farther laterally than the lateral extent of the supracetabular crest and it also reaches as far as the farthest lateral point on the dorsal border of the iliac blade (Figs. 5D, 5F, 9B and 9C). Consequently, the marked width of the brevis fossa stands in sharp contrast to the narrow condition observed in other unenlagiines such as *U. comahuensis*, *U. paynemili* and *Rahonavis* (Novas & Puerta, 1997; Forster et al., 1998; Calvo, Porfiri & Kellner, 2004). These unenlagiines have a distinctively reduced brevis fossa, in contrast to *Buitreraptor* and other dromaeosaurids, a character also observed in basal avialans and considered as a derived feature (Novas, 2004), as for example in *Confuciusornis* which lacks a fossa altogether (Chiappe et al., 1999). The brevis fossa is concave with a lateral border projected farther ventrally than the medial one so that the fossa faces mostly ventrally, but also slightly medially and is therefore not visible in lateral view (Figs. 5E and 9A). On the other hand, in *U. comahuensis*, *U. paynemili* and *Rahonavis* the fossa faces mostly medially. The medial border is less prominent than the lateral one, but is proportionately more robust than in either *U. comahuensis*, *U. paynemili* or *Rahonavis* in which the medial border is very subtly developed. A spatulate shape of the brevis fossa in ventral view caused by the transverse expansion of the distal end is a derived trait shared by *Buitreraptor*, *U. comahuensis* and *U. paynemili* (Figs. 5F and 9C). In *Rahonavis*, by contrast, the very narrow brevis fossa has an elliptical outline with a pointed end, a similar condition to that of eudromaeosaurids such as *Deinonychus* (Ostrom, 1969).

##### Pubis

Only the right pubis of MPCA 238 is preserved (Figs. 5A–5C) in articulation with the pubic penduncle, although the articular region is poorly preserved and the pubis seems to be displaced from its natural position. The distal portion of the bone is missing with the break situated proximal to the boot, so the morphology of the distal articulation with the opposite pubis is unknown. Because the pubis is incomplete, its total length and the ratio between its length and that of the femur are unknown. However, the preserved portion of the pubis exceeds half the length of the femur of the holotype, taking into account that MPCA 238 and the holotype represent individuals of very similar sizes and proportions. Also this length compares favorably with the more complete elements known for MPCN-PV-598 (Novas et al., 2018).

Overall the pubis has an approximately sigmoid shape in lateral view, with a slightly concave anterior edge proximally where it borders the iliac peduncle, and an anteriorly convex curvature distally (Figs. 5A and 5C). A similar sigmoid curvature with an anteriorly concave profile in the peduncular region and a longer gently backwardly curving shaft and boot is also observed in *Rahonavis* (Forster et al., 1998). The distal portion of the pubic shaft is also posteriorly curved, as in *U. paynemili* (Calvo, Porfiri & Kellner, 2004), but the posterior curvature reported in the pubis of *U. comahuensis* is a taphonomic artifact, caused by the fracture of the shaft (Federico A. Gianechini, 2010, personal observation). In these unenlagiines, the pubic shaft curvature is gradual and unlike the kinked pubes observed in microraptorines (e.g., *Sinornithosaurus*, Xu, 2002; *Microraptor*, Xu et al., 2003; *Hesperonychus*, Longrich & Currie, 2009; *Changyuraptor*, Han et al., 2014). The peduncular portion of the pubis in *Buitreraptor* is anteroposteriorly expanded and is transversely compressed, but due to breakage neither peduncular articular surface is well preserved. The pubic shaft narrows distally in lateral view so that the distal section is less than half of the anteroposterior width of the proximal shaft section.

Independent of its curvature, the pubis of *Buitreraptor* is vertically oriented below the pubic peduncle of the ilium, a condition also observed in the pubes of *U. comahuensis*, *Rahonavis* and some troodontids such as *Gobivenator* (Tsuihiji et al., 2014). A vertical pubic orientation is also observed in the pubis of MPCN-PV-598 (Novas et al., 2018). Pubic orientation in the unenlagiines differs from the true opisthopubic condition of most dromaeosaurids, such as *Hesperonychus*, *Sinornithosaurus*, *Deinonychus*, *Velociraptor* and *Adasaurus* (Ostrom, 1976b; Barsbold, 1983; Norell & Makovicky, 1997, 1999; Xu, 2002; Longrich & Currie, 2009; Turner, Makovicky & Norell, 2012), and avialans. In anterior view, the lateral border of the pubis is straight and ventromedially inclined (Fig. 5B), and lacks the sigmoid lateral contour observed in *U. comahuensis* and *U. paynemili* (Novas & Puerta, 1997; Calvo, Porfiri & Kellner, 2004).

The medial surface of the pubis projects medially as a ridge that would have contacted a similar ridge of the opposite pubis, forming a pubic apron, as corroborated in MPCN-PV-598. The proximal end of this ridge is located distal to the base of the ischiadic peduncle and close to the posterior border of the shaft in MPCA 238 (Figs. 5B and 5C). Conversely, in *U. comahuensis*, *U. paynemili* and *Rahonavis* the proximal end of the pubic apron is located farther distally, closer to the middle section of the shaft (MCF PVPH 78; FMNH PR 2830). The proximal section of the apron has the form of a shallow transversely oriented flange, but distally it is significantly medially expanded and is posteriorly concave. Also, it is gradually displaced anteriorly along the pubic shaft so that the distal part of this flange arises from the anterior surface of the shaft.

The shaft has well-defined anterior and posterior borders in its proximal portion, although it becomes transversely expanded and anteroposteriorly compressed distally, so that the anterior and posterior borders are less defined. In fact, the distal part of the shaft has a convex anterior surface and a concave posterior one. The shaft lacks a lateral tubercle near its midsection, such as is seen in microraptorines including *Hesperonychus*, *Sinornithosaurus* and *Microraptor* (Hwang et al., 2002; Xu, 2002; Longrich & Currie, 2009).

##### Ischium

The right ischium of the holotype is the only ischial element preserved among known specimens of *Buitreraptor* (Figs. 9D and 9E). It is significantly shorter than the pubis of MPCA 238, but the latter is incomplete so we estimate that the ischium has a length less than 50% of the length of the pubis. A similar ratio is observed in the ischium of *U. comahuensis*, *Rahonavis* and other paravians such as *Sinornithosaurus*, *Sinovenator*, *Archaeopteryx* and *Confuciusornis* (Wellnhofer, 1974; Ostrom, 1976a; Chiappe et al., 1999; Xu, 2002; Xu et al., 2002). The ischium is transversely compressed and laminar, as in *U. comahuensis*, *Rahonavis* and other dromaeosaurids as *Velociraptor*, *Sinornithosaurus* and *Microraptor* (Norell & Makovicky, 1997; Hwang et al., 2002; Xu, 2002), troodontids as *Sinornithoides* and *Sinovenator* (Russell & Dong, 1993; Xu, 2002), and basal avialans including *Archaeopteryx*, *Confuciusornis* and *Sapeornis* (Wellnhofer, 1974; Chiappe et al., 1999; Zhou & Zhang, 2003b).

The iliac peduncle is proximally located and has a rectangular shape in lateral view, and a triangular outline in proximal view. Anterodistal to it, the ischiadic portion of the acetabulum forms a large concave border. The pubic pedunce is anteriorly located and separated from the iliac peduncle by the concavity of the acetabulum. The pubic peduncle is also rectangular although dorsoventrally expanded and wider than the iliac one. Conversely, in *U. comahuensis* the iliac peduncle is more anteroposteriorly expanded and it is continuous with the pubic process, without a concave border between them. Moreover, in *U. comahuensis* the pubic peduncle is more dorsally located, is not anteriorly projected, and its articular surface is anterodorsally oriented (Novas & Puerta, 1997). The iliac peduncle of *Rahonavis* is small and vertical, whereas the pubic peduncle was not preserved (Federico A. Gianechini, 2012, personal observation).

A small, tuber-like posterodorsal process is present along the posterior border. This process is prominent in both *U. comahuensis* and *Rahonavis*, although in these taxa it is hook-shaped and posterodorsally projected, and separated from the ischial shaft by a notch (Novas & Puerta, 1997; Forster et al., 1998). This process is present only in basal dromaeosaurids, such as *Microraptor* and *Sinornithosaurus* (Hwang et al., 2002; Xu, 2002), whereas in more derived dromaeosaurids the ischium lacks a posterior processes. This structure is also present in some troodontids as *Sinovenator* and *Mei* (Xu, 2002; Xu & Norell, 2004), in which it has a similar development than in *Buitreraptor*, and is also present in basal avialans as *Archaeopteryx*, *Jeholornis* and *Confuciusornis* (Wellnhofer, 1974; Chiappe et al., 1999; Zhou & Zhang, 2002).

A longitudinal, thin ridge extends along the lateral surface of the shaft (Fig. 9D), which is similar but sharper than those observed in *U. comahuensis*, *Rahonavis*, *Velociraptor, Sinornithosaurus*, and *Deinonychus* (Novas & Puerta, 1997; Norell & Makovicky, 1997; Ostrom, 1969, Xu, 2002), in which the lateral ridge is rounded. A well-developed obturator process is present. It is triangular in lateral view, projects anteriorly and terminates in a pointed end, similar to that of *U. comahuensis*, *Rahonavis*, *Anchiornis*, *Sinornithosaurus* and *Microraptor* (Novas & Puerta, 1997; Forster et al., 1998; Xu, 2002; Hu et al., 2009; Hwang et al., 2002). The obturator process projects from about the midsection of the shaft of the ischium, as is also the case in *U. comahuensis*, *Sinornithosaurus*, *Deinonychus*, *Velociraptor* and *Tianyuraptor* (Ostrom, 1969; Norell & Makovicky, 1997; Novas & Puerta, 1997; Xu, 2002; Zheng et al., 2010), and troodontids such as *Saurornithoides* and *Sinornithoides* (Osborn, 1924; Russell & Dong, 1993; Norell et al., 2009). By contrast, in *Rahonavis*, *Microraptor* and basal avialans such as *Archaeopteryx*, the obturator process is distally located (Wellnhofer, 1974; Forster et al., 1998; Hwang et al., 2002) although body size may influence this trait. The ischium is posterodistally expanded into a triangular process with a pointed end, similar to that observed in *U. comahuensis*, *Sinornithosaurus* and *Tianyuraptor* (Novas & Puerta, 1997; Xu, 2002; Zheng et al., 2010). The anterior border of the shaft is concave in lateral view and is dorsally continuous with the distal edge of the pubic peduncle and distally with the dorsal edge of the obturator process, similarly to the ischium of *U. comahuensis* and *Sinornithosaurus* (Novas & Puerta, 1997; Xu, 2002). The ventral border of the ischium forms a broad, shallow notch between the distal tip and the obturator process. The medial surface of the bone is flat and smooth (Fig. 9E).

#### Hindlimb

##### Femur

**Figure 10:**
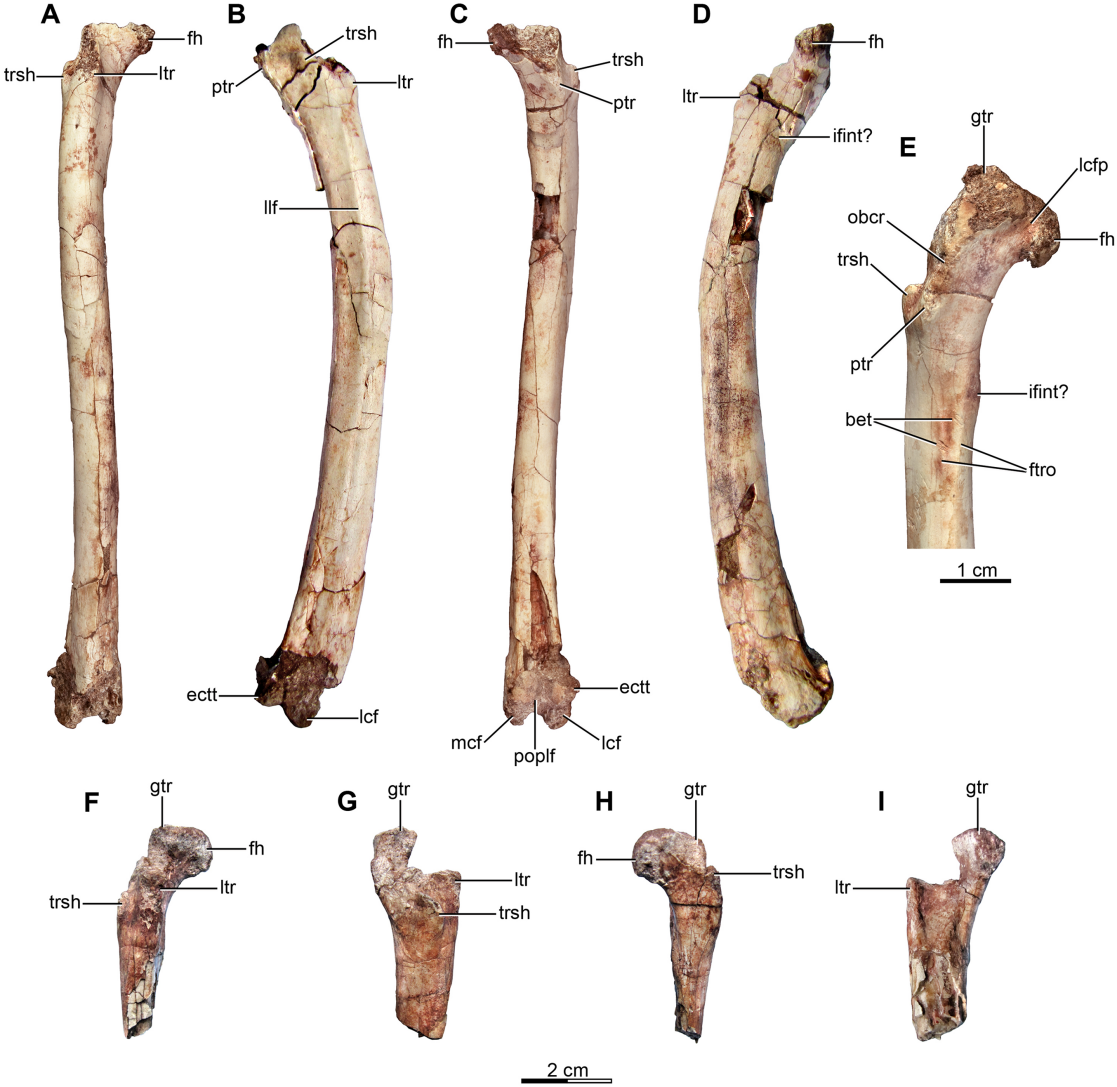
Femur of the holotype (MPCA 245) and referred specimen (MPCA 238) of *Buitreraptor gonzalezorum*. (A–D) Right femur of MPCA 245, in (A) anterior, (B) lateral, (C) posterior (D) and medial view. (E) Detail of the proximal portion of the left femur of MPCA 245, in posterior view. (F–I) proximal portion of the right femur of MPCA 238, in (F) anterior, (G) lateral, (H) posterior and (I) medial view. Scales: 2 cm for A–D and F–I, 1 cm for E. bet, bioerosion trace fossils; ectt, ectocondylar tuberosity; fh, femur head; ftro, fourth trochanter; gtr, greater trochanter; ifint?, possible insertion point of the *M. iliofemoralis internus*; lcf, lateral condyle of the femur; lcfp, passage for the *ligamentum capitis femoris*; llf, lateral line of the femur; ltr, lesser trochanter; mcf, medial condyle of the femur; obcr, obturator crest; poplf, popliteal fossa; ptr, posterior trochanter; trsh, trochanteric shelf.

The holotype preserves both femora which are nearly complete (Figs. 10A–10E), although the distal portion of the left one is missing. Additionally, MPCA 238 preserves the proximal portion of the right femur (Figs. 10F–10I). In general, the femoral shaft is bowed and anteriorly convex (Figs. 10B and 10D), whereas in anterior view it is slightly sigmoid due the shaft being laterally convex in the proximal part and medially convex distally (Fig. 10A). A bowed femur is common among Maniraptora, and is present in oviraptorosaurs such as *Nomingia* (Barsbold et al., 2000), alvarezsaurids such as *Mononykus* (Perle et al., 1994), and in paravians including *Rahonavis*, *Mahakala*, *Sinovenator*, *Sinornithoides*, *Saurornithoides*, *Gobivenator*, *Velociraptor*, *Sinornithosaurus*, *Microraptor*, *Bambiraptor* and *Archaeopteryx* (Wellnhofer, 1974; Ostrom, 1975, 1976a; Currie & Peng, 1993; Russell & Dong, 1993; Forster et al., 1998; Norell & Makovicky, 1999; Currie & Dong, 2001; Elzanowski, 2002; Hwang et al., 2002; Xu, 2002; Burnham, 2004; Turner, Pol & Norell, 2011; Tsuihiji et al., 2014). The curvature observed in *Buitreraptor* is much more marked than in the femur of *U. comahuensis* and *Neuquenraptor*, but is similar to that observed in *Rahonavis* (Novas & Puerta, 1997; Forster et al., 1998; Novas & Pol, 2005).

The femoral head is medially and slightly anteriorly projected, and is set perpendicular to the anteroposterior axis of the bone. A distinct neck between the head and shaft is not present, similarly to the condition of *Rahonavis*, *Sinornithosaurus* and *Archaeopteryx* (Wellnhofer, 1974; Ostrom, 1976a; Forster et al., 1998; Xu, 2002). The head is better preserved on the left femur of the holotype and in MPCA 238 (Figs. 10E, 10F and 10H), where it is convex and rounded and exhibits a posteroventrally projected lip. This lip delimits a groove posteriorly that corresponds to the passage of the *ligamentum capitis femoris* (*sensu* Baumel & Witmer, 1993), which is also present in *U. comahuensis* (MCF PVPH 78) and many other theropods.

The greater trochanter is located lateral to the head and forms the proximalmost point on the bone, although it is poorly preserved in both specimens. The anterior surface of the proximal portion is broken in both femora of the holotype and also in the femur of MPCA 238, but they all preserve the ventral base of the lesser trochanter as an anteriorly projected protuberance (Figs. 10A, 10B, 10D, 10F, 10G and 10I). Because of breakage we cannot confirm if it was fully fused with the greater trochanter to form a trochanteric crest, as in *Rahonavis*, some troodontids such as *Sinornithoides* (Currie & Dong, 2001), basal avialans as *Confuciusornis* (Chiappe et al., 1999), and more derived Avialae. On the other hand, in *U. comahuensis* the greater and the lesser trochanters are separated by a small groove or slit, as in *Deinonychus* (MCZ 4371) and more basal coelurosaurs. Another notable feature of the lesser trochanter of *Buitreraptor* is its relatively distal location with respect to the proximal end of the femur, as also is observed in the specimen MPCN-PV-598. This condition differs from *Unenlagia*, other dromaeosaurids such as *Velociraptor*, and other paravians in which the lesser trochanter is located relatively farther proximally (Novas et al., 2018).

The lateral surface of the proximal end of the shaft is marked by a prominent trochanteric shelf (following Hutchinson, 2001), with a well-defined proximal border and an anterodorsally projected proximal end (Figs. 10A–10C and 10F–10H). This morphology of the trochanteric shelf is very similar to that observed in *Mahakala*, *Rahonavis*, and troodontids such as *Gobivenator* (Forster et al., 1998; Turner et al., 2007; Turner, Pol & Norell, 2011; Tsuihiji et al., 2014). This feature also appears to be present in *U. comahuensis*, but it is much less developed in this taxon. The posterior trochanter is located immediately posterior to the trochanteric shelf on the posterolateral surface of the shaft and is shaped as a low, elongate tubercle (Figs. 10B, 10C and 10E). It is proximally continuous with a ridge extending toward the greater trochanter, a configuration very similar to that present in *Rahonavis* (Federico A. Gianechini, 2012, personal observation) and in *Velociraptor*, *Sinornithosaurus*, *Microraptor*, *Sinovenator* and *Talos* (Norell & Makovicky, 1999; Xu, 2002; Zanno et al., 2011). This ridge may be homologous to the obturator crest observed in avialans, including extant birds (Baumel & Witmer, 1993). In *U. comahuensis* the posterior trochanter is not connected to a ridge. The obturator crest described in *Austroraptor* is shallower and is located close to the posteromedial border of the femur (Novas et al., 2009), thus differing from the taxa cited above. A raised and rugose surface that is approximately circular in shape is observed on the medial surface close to the posterior border and distal to the level of the lesser trochanter (Figs. 10D and 10E). This surface could be homologous with the insertion point of the *M. iliofemoralis internus* present in birds (Baumel & Witmer, 1993; Hutchinson, 2001).

Two low, longitudinal and subparallel ridges, which delimit a poorly defined groove between them, are observed on the posterior surface of the shaft close to the insertion point of the *M. iliofemoralis internus* (Fig. 10E), and are also present in MPCN-PV-598 (Novas et al., 2018). We interpret these ridges as corresponding to a vestige of the fourth trochanter muscle insertion mainly due their location. *Unenlagia* and *Neuquenraptor* exhibit a similar morphology of the fourth trochanter region on the posterior face of the femoral shaft (see Novas et al., 2018). This trochanter is also vestigial in *Austroraptor* and *Rahonavis*, and also reduced or absent in other paravians as *Sinornithosaurus*, *Microraptor*, *Sinovenator*, *Sinornithoides*, *Archaeopteryx* and more derived avialans (Russell & Dong, 1993; Elzanowski, 2002; Xu, 2002), as well as in many oviraptorosaurs (Makovicky & Sues, 1998) and alvarezsauroids (Makovicky, Apesteguía & Gianechini, 2012). In *Mahakala* this trochanter forms a very low and poorly developed ridge-like structure (Turner, Pol & Norell, 2011). In more derived Laurasian dromaeosaurids the fourth trochanter is generally absent, as in *Deinonychus* (Ostrom, 1976b), although is well-developed in some larger specimens of *Velociraptor* (Norell & Makovicky, 1999).

The femoral shaft is slightly transversely compressed along its length, although close to the distal end it is slightly anteroposteriorly expanded. A low longitudinal crease is observed extending along the lateral surface from the base of the trochanteric shelf to the distal section and ending close to the lateral articular condyle. This corresponds to the lateral intermuscular line (following Hutchinson, 2001), an anatomical feature indicating the separation between two muscle bundles in contact with the femur (Fig. 10B). This line is also observed in the femur of *Neuquenraptor*, whereas in *Rahonavis* a lateral line or crease along the surface of the femoral shaft is present, but is posteriorly displaced with its proximal end far from the trochanteric crest (Federico A. Gianechini, 2012, personal observation). On the other hand, a lateral intermuscular line is not present in either *U. comahuensis* or *Austroraptor*. In other dromaeosaurids such as *Velociraptor* and *Mahakala* (Norell & Makovicky, 1999; Norell et al., 2009; Turner, Pol & Norell, 2011), and in troodontids such as *Sinornithoides*, *Saurornithoides*, *Linhevenator* and *Talos* (Currie & Dong, 2001; Norell et al., 2009; Xu et al., 2011; Zanno et al., 2011), the lateral intermuscular line is well-developed. The posterior surface of the femoral shaft bears a posterior intermuscular line (*linea intermuscularis caudalis* of Hutchinson, 2001), which is less conspicuous than the lateral intermuscular line and extends from the proximal shaft region to the proximal end of the lateral condyle and connects with the ectocondylar tuber. The distal segment of this line is very similar to the posterior crest that contacts the lateral condyle in *Mahakala* (Turner et al., 2007; Turner, Pol & Norell, 2011). A posterior intermuscular line is also very conspicuous in *Rahonavis*, in which it contacts the ectocondylar tuber distally, whereas in *Neuquenraptor* it is present but is absent in *U. comahuensis*. The anterior surface bears a faint anterior intermuscular line (*linea intermuscularis cranialis* of Hutchinson, 2001), which is much less distinct than the other two intermuscular lines. It extends from the base of the lesser trochanter to the anterior edge of the lateral distal condyle while bowing gently medially at midlength.

The distal region of the femur is characterized by a posterior, longitudinal and deep popliteal fossa (Fig. 10C). However, the depth of this fossa, as well as its proximal extent, are exaggerated by crushing and a collapse of the bone surface. The poplietal fossa is delimited laterally by the distal end of the posterior intermuscular line and medially by the adductor crest (*sensu* Hutchinson, 2001; *crista supracondylaris medialis* of the birds, *sensu* Baumel & Witmer, 1993). The articular condyles are only preserved on the right femur, although eroded (Figs. 10B and 10C). They are separated by an intercondylar groove, and have convex articular surfaces that project ventrally, but are also posteriorly expanded to a modest degree. They are transversely narrow, in contrast to the distal condyles of the femur of *U. comahuensis*. The lateral condyle is slightly larger than the medial one, and is surmounted posteriorly by the ectocondylar tuber, which is roughly spherical in shape and extends beyond the posterior border of the condyles (Figs. 10B and 10C). This structure is separated from the lateral condyle by a marked groove. The ectocondylar tuber is much more developed in *U. comahuensis* and is also vertically more expanded, as also occurs in *Rahonavis*.

##### Tibia and proximal tarsals

**Figure 11:**
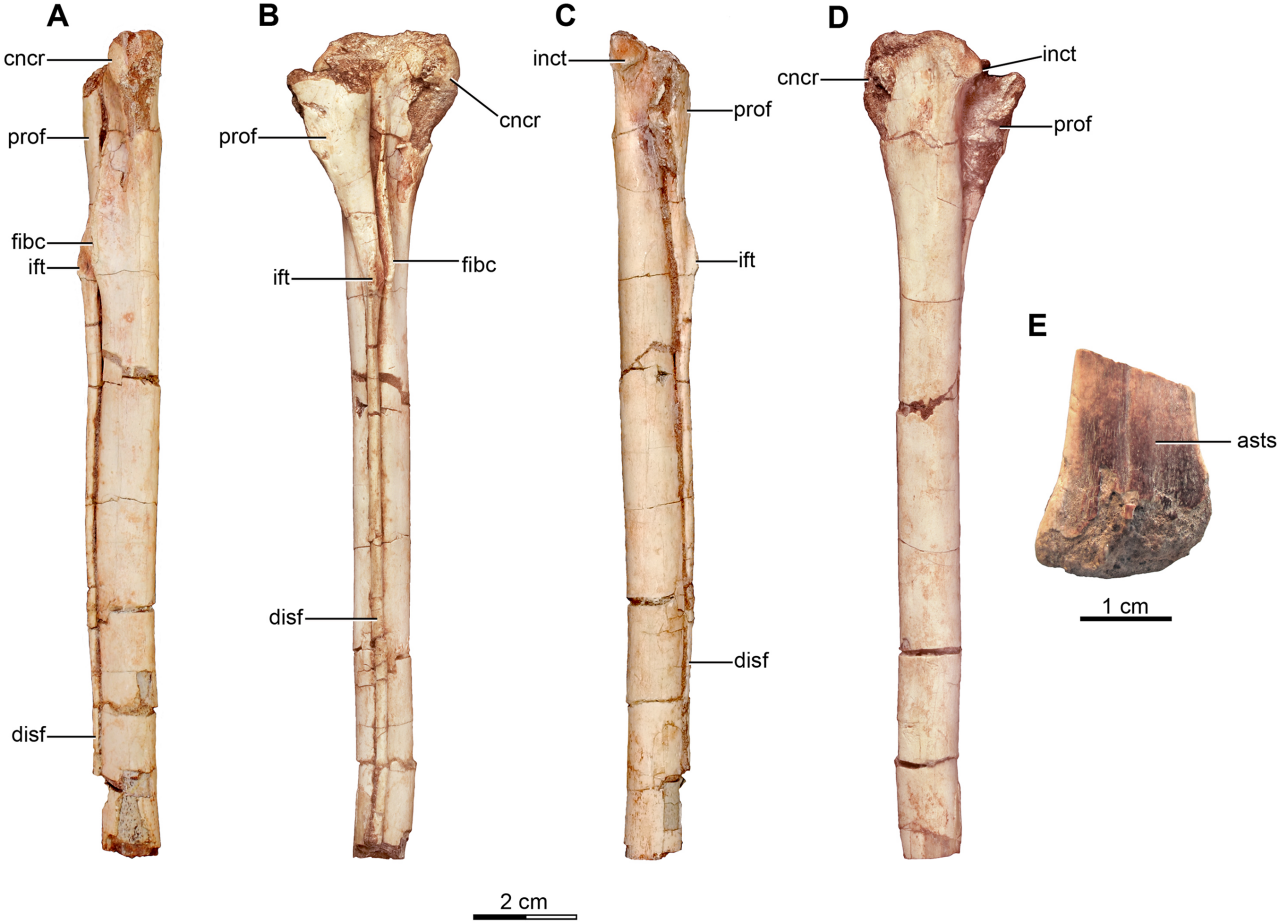
Right tibia and fibula of the holotype of *Buitreraptor gonzalezorum* (MPCA 245). (A) Anterior, (B) lateral, (C) posterior and (D) medial view. (E) Distal portion of the right tibia, in anterior view. Scale: 2 cm for A–D, 1 cm for E. asts, articular surface for the ascendant process of the astragalus; cncr, cnemial crest; disf, distal part of the fibular shaft; fibc, fibular crest; ift, *iliofibularis* tubercle; inct, internal condyle of the tibia; prof, proximal part of the fibular shaft.

**Figure 12:**
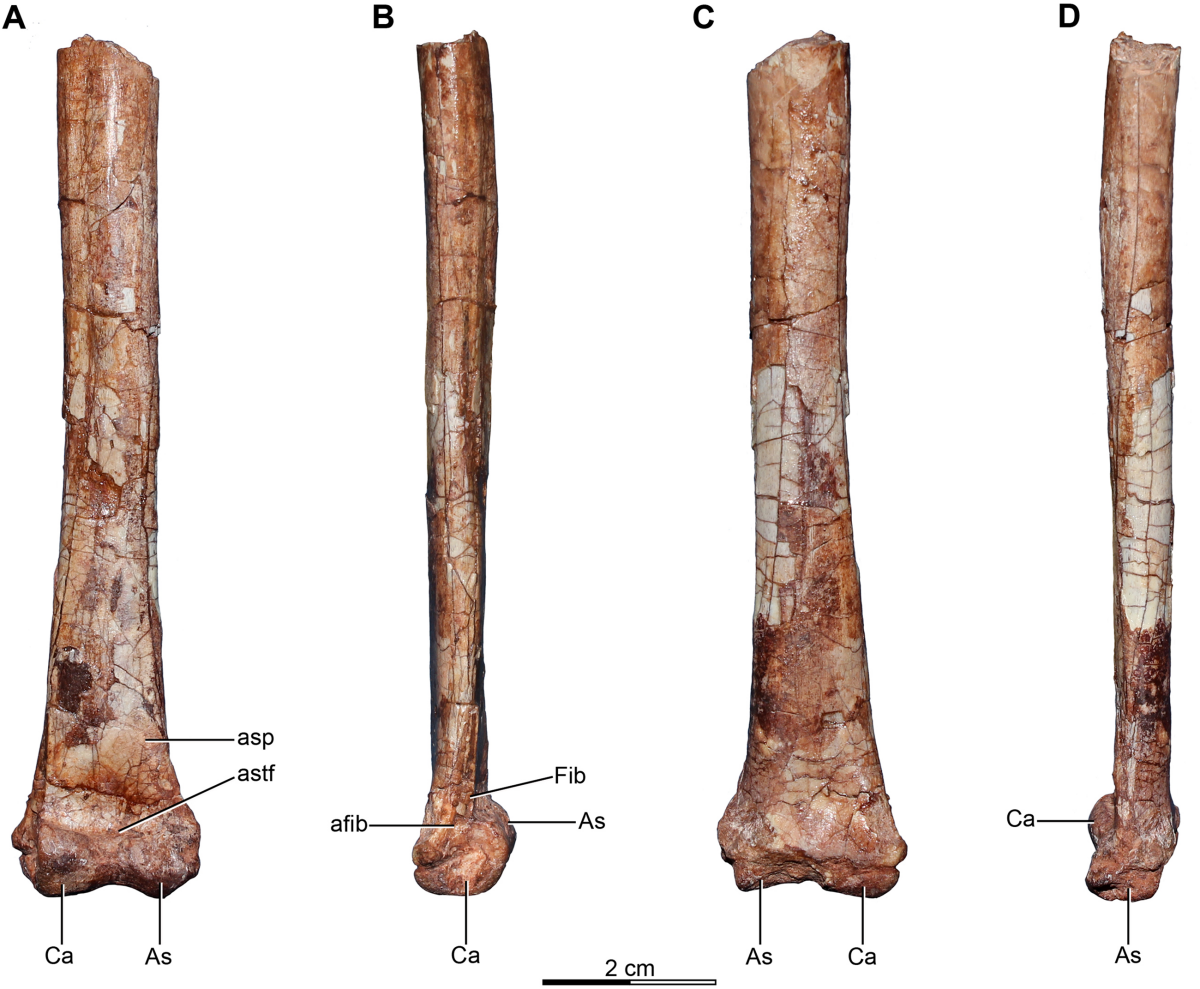
Incomplete right tibia articulated with the proximal tarsals of the referred specimen of *Buitreraptor gonzalezorum* (MPCA 238). (A) Anterior, (B) lateral, (C) posterior and (D) medial view. As, astragalus; afib, articular surface for the fibula; asp, ascendant process of the astragalus; astf, anterior fossa of the astragalus; Ca, calcaneum; Fib, fibula.

These elements are present both in the holotype and MPCA 238 (Figs. 11 and 12). The holotype includes the almost complete right tibia in articulation with the fibula, but with its distal portion preserved separately. The left tibia only preserves the proximal portion articulated with the proximal section of the fibula. MPCA 238 includes the distal half of the right tibia articulated with the astragalus and the calcaneum, and a portion of the shaft of the left tibia lacking both articular ends. An estimate of its complete length based on both specimens indicates that the tibia is longer than the femur, as also occurs in *U. comahuensis*, *Velociraptor*, *Linheraptor*, *Tianyuraptor*, *Microraptor*, *Rahonavis* and *Mahakala* (Forster et al., 1998; Norell & Makovicky, 1999; Hwang et al., 2002; Xu, 2002; Xu et al., 2010, 2015; Zheng et al., 2010; Turner, Pol & Norell, 2011), and troodontids as *Sinovenator* and *Anchiornis* (Xu, 2002; Xu et al., 2008; Hu et al., 2009). In fact, in MPCN-PV-598 the tibia is 125% of femur length (Novas et al., 2018).

The proximal articular surface is poorly preserved but exhibits a triangular shape in proximal view. The anterior edge is expanded to form the cnemial crest, which is triangular in lateral view (Fig. 11B). The anterior edge of the crest is slightly laterally curled so that the lateral surface of the crest is concave, as in *Rahonavis* and as is common in many coelurosaurs (Ostrom, 1969; Novas, 1997; Norell & Makovicky, 1999; Barsbold et al., 2000; Norell, Makovicky & Clark, 2000; Kobayashi & Lü, 2003; Hwang et al., 2004). The poor or non-preservation of the cnemial crest in *Austroraptor* and *U. comahuensis* precludes comparisons. The fibular crest rises on the lateral surface close to the proximal end, and is formed as a gentle arc that is slightly anteriorly deflected and bears a sharp edge (Figs. 11A and 11B). The internal condyle is located on the posterior border of the proximal portion of the tibia and is conical in shape with a pointed end (Figs. 11C and 11D), as in *Rahonavis* and other coelurosaurs (e.g., alvarezsaurids; Perle et al., 1994; Novas, 1997). It is separated from the fibular condyle by a sharp notch.

The tibial shaft is straight and has a mainly triangular cross section although it grades to a more circular shape distally. Both tibiae of MPCA 238 have a more anteroposteriorly compressed shaft, a feature evident in the distal section (Fig. 12) although this could in part be due to taphonomic distortion. The anterolateral surface of the distal end of the tibia of MPCA 238 shows a faint longitudinal groove for reception of the fibula, and the shaft is transversely expanded distally (Figs. 12A–12C). The distal articular surface is preserved on the right tibiae of both MPCA 238 and the holotype specimen (Figs. 11E and 12), and the lateral malleolus projects farther distally than the medial one as is typical for theropods. The distal fragment of the right tibia of the holotype is not articulated with the proximal tarsals, but its anterior surface reveals the articular surface for the ascending process of the astragalus (Fig. 11E). This surface is mainly smooth but is vertically crossed by a straight groove that is slightly laterally displaced and marks the edge of the articulation with the ascending process of the astragalus. The posterior distal surface is gently concave between low posterolateral and a posteromedial ridges. The posterolateral ridge is close to the lateral border of the shaft and it is more laterally projected than the posteromedial ridge. The lateral malleolus of the tibia projects slightly beyond the calcaneum. In caudal view, the astragalus abuts the end of the tibia, but does not appear to wrap onto its caudal surface so that the malleoli are visible.

The astragalus and the calcaneum are preserved in MPCA 238, and are fused to each other. Fusion between the proximal tarsals is also observed in *Neuquenraptor* and *Austroraptor* (Novas & Pol, 2005; Currie & Paulina Carabajal, 2012), and in many other dromaeosaurids such as *Mahakala*, *Sinornithosaurus*, *Microraptor*, *Graciliraptor*, *Velociraptor* and *Linheraptor* (Norell & Makovicky, 1999; Hwang et al., 2002; Xu, 2002; Xu et al., 2010, 2015; Turner, Pol & Norell, 2011), whereas in *Deinonychus* these bones are not fused (Ostrom, 1969). In *Rahonavis* the fusion between proximal tarsals is partial (Forster et al., 1998), and a similar condition presents in the basal avialans *Archaeopteryx* and *Jeholornis* (Wellnhofer, 1992; Zhou & Zhang, 2002). Both bones are firmly articulated with the tibia in MPCA 238, but do not appear to be fused to the tibia to form a proper tibiotarsus, as also is the case in *Neuquenraptor*, *Austroraptor* and *Rahonavis*. A tibiotarsus is not common among non-avialan coelurosaurs, although it is observed in some dromaeosaurids such as *Graciliraptor*, *Microraptor* and *Balaur* (Xu, Zhou & Wang, 2000; Hwang et al., 2002; Xu & Wang, 2004b; Csiki et al., 2010; Brusatte et al., 2013; Pei et al., 2014) and troodontids such as *Troodon* and *Mei* (Russell, 1969; Russell & Dong, 1993; Gao et al., 2012). The medial and lateral condyles are strongly anteriorly projected and separated by a wide sulcus (Fig. 12A). The astragalus bears a wide and long ascending process proximally, which is roughly triangular in shape, with an almost vertical rather than sloping lateral border. Despite a proximal break the ascending process is clearly taller than wide, and it exceeds twice the height of the body of the astragalus as in *Rahonavis* (Federico A. Gianechini, 2012, personal observation), possibly in *Austroraptor* (Currie & Paulina Carabajal, 2012), and in other coelurosaurs (Rauhut, 2003). Unfortunately this process is broken close to its base in *Neuquenraptor* and thus its height is unknown (Novas & Pol, 2005). The ascending process is separated from the body of the astragalus by a broad and shallow transverse groove (Fig. 12A). A small triangular slot is formed on the lateral surface of the crus between the calcaneum, the shaft of the tibia and the ascending process, into which the distal end of the fibula would fit (Fig. 12B), as is also observed in *Velociraptor* (Norell & Makovicky, 1999) and *Neuquenraptor* (Federico A. Gianechini, 2010, personal observation). *Rahonavis* lacks such a notch for the fibula, even on the calcaneum, suggesting that the fibula did not contact the tarsals (FMNH PR 2830). The calcaneum has a shallow fossa on its lateral face like in many other theropods.

##### Fibula

The right fibula is almost complete in the holotype (Figs. 11A–11D). It is a slender bone with an anteroposteriorly expanded and transversely compressed proximal portion. The proximal end of the bone is displaced to lie behind rather than lateral to the fibular crest so that the posterior edge of the proximal portion projects beyond the posterior border of the tibia (Figs. 11B and 11D). The lateral surface of the proximal end is slightly convex whereas the medial surface apparently lacks a medial fossa, although it is poorly preserved.

The anterior border of the proximal portion is vertical oriented whereas the posterior border is anteroventrally inclined, and the part of the fibula has a triangular outline in lateral view. The fibula narrows ventrally towards the iliofibularis tubercle, and below the tubercle most of the shaft of the fibula is abruptly reduced to a slender bony rod with a diameter less than one fifth of that of the tibia. A similar reduction of the shaft is also observed in *Neuquenraptor* and is common among Maniraptora (Ostrom, 1976a; Russell & Dong, 1993; Currie & Dong, 2001; Makovicky & Sues, 1998; Clark, Norell & Chiappe, 1999; Burnham et al., 2000; Burnham, 2004; Hwang et al., 2002; Xu, 2002). The iliofibularis tubercle is developed as a short, proximodistally elongated and anterolaterally projected ridge (Figs. 11A–11C). This feature is located at the same level as the widest part of the fibular crest and probably the muscular insertion is formed by both protuberances together.

The distal end of the fibula is not preserved in most of the specimens, except in MPCN-PV-598 in which the fibular contact with the proximal tarsals is observed. In MPCA 238 small osseous fragments close to the dorsal border of the calcaneum likely correspond to the distal end of the fibula (Fig. 12B). In many paravians, including troodontids such as *Sinornithoides* (Russell & Dong, 1993; Currie & Dong, 2001), dromaeosaurids such as *Microraptor* and *Velociraptor* (Norell & Makovicky, 1999; Hwang et al., 2002; Xu, 2002), and basal avialans such as *Archaeopteryx* (Ostrom, 1976a), the fibula reaches the proximal tarsals, as in many other Maniraptora. Conversely, in *Rahonavis*, *Mahakala* and most avialans such as *Confuciusornis*, *Yanornis* and *Zhongjianornis* (Chiappe et al., 1999; Zhou & Zhang, 2001; Zhou, Zhang & Li, 2010; Turner, Pol & Norell, 2011), the fibula terminates proximal to the ankle, though the size of the gap varies considerably.

##### Metatarsals

**Figure 13:**
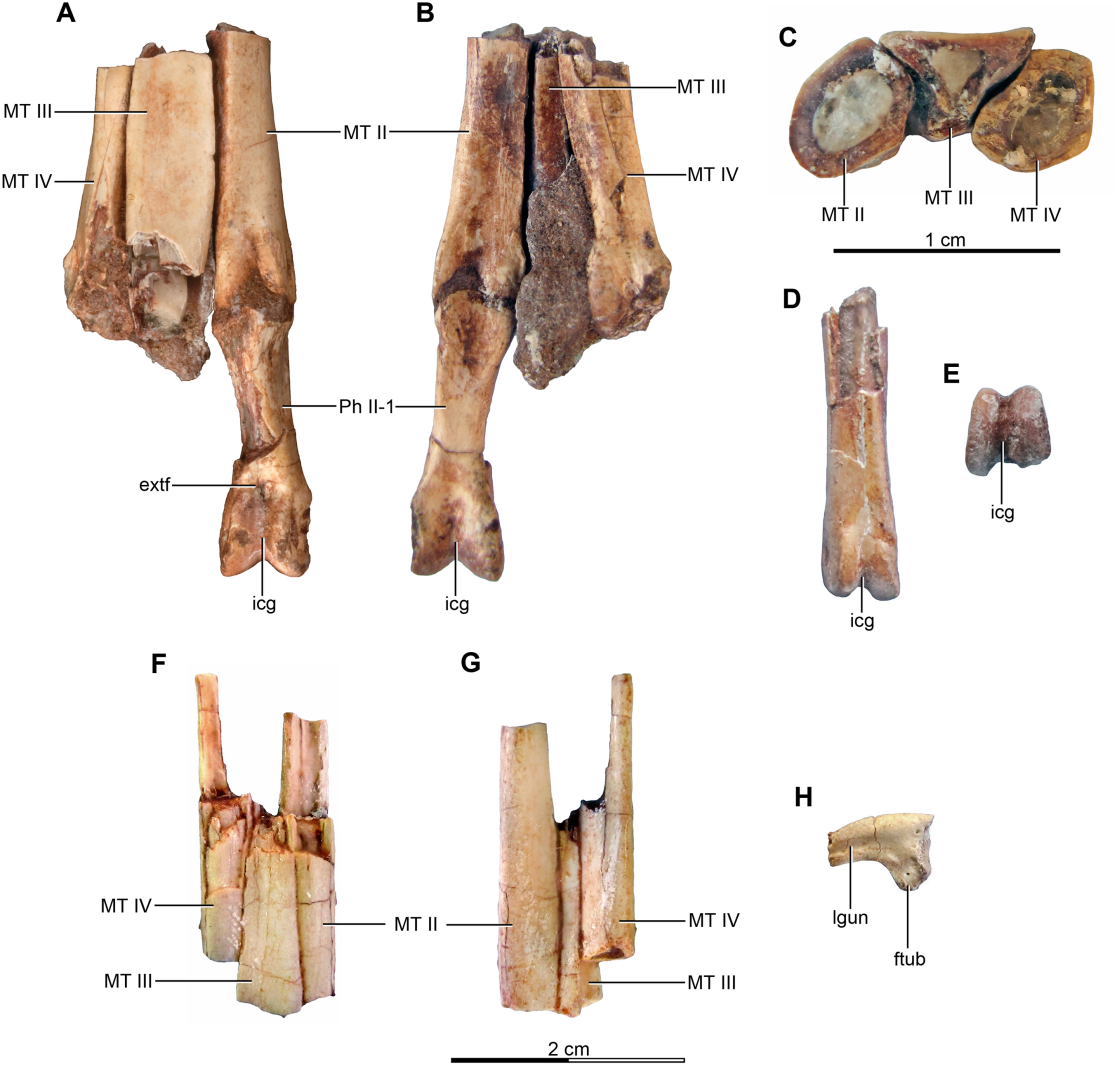
Metatarsus and pedal phalanges of the holotype (MPCA 245) and referred specimen (MPCA 471-D) of *Buitreraptor gonzalezorum*. (A, B) Articulated distal portions of the right metatarsals II, III and IV, and pedal phalanx II-1 of MPCA 245, in (A) anterior and (B) posterior view. (C) Transverse section of right metatarsal II, III and IV of MPCA 245, in proximal view. (D, E) Distal portion of the left metatarsal II of MPCA 245, in (D) anterior and (E) distal view. (F, G) Fragmentary articulated right metatarsals II, III and IV of MPCA 471-D, in (F) anterior and (G) posterior view. (H) Indeterminate pedal ungual phalanx of MPCA 471-D, in side view. Scales: 2 cm for A, B and D–H, 1 cm for C. extf, extensor fossa; ftub, flexor tubercle; icg, intercondylar groove; lgun, lateral groove of the ungual; MT, metatarsal; Ph, phalanx.

**Figure 14:**
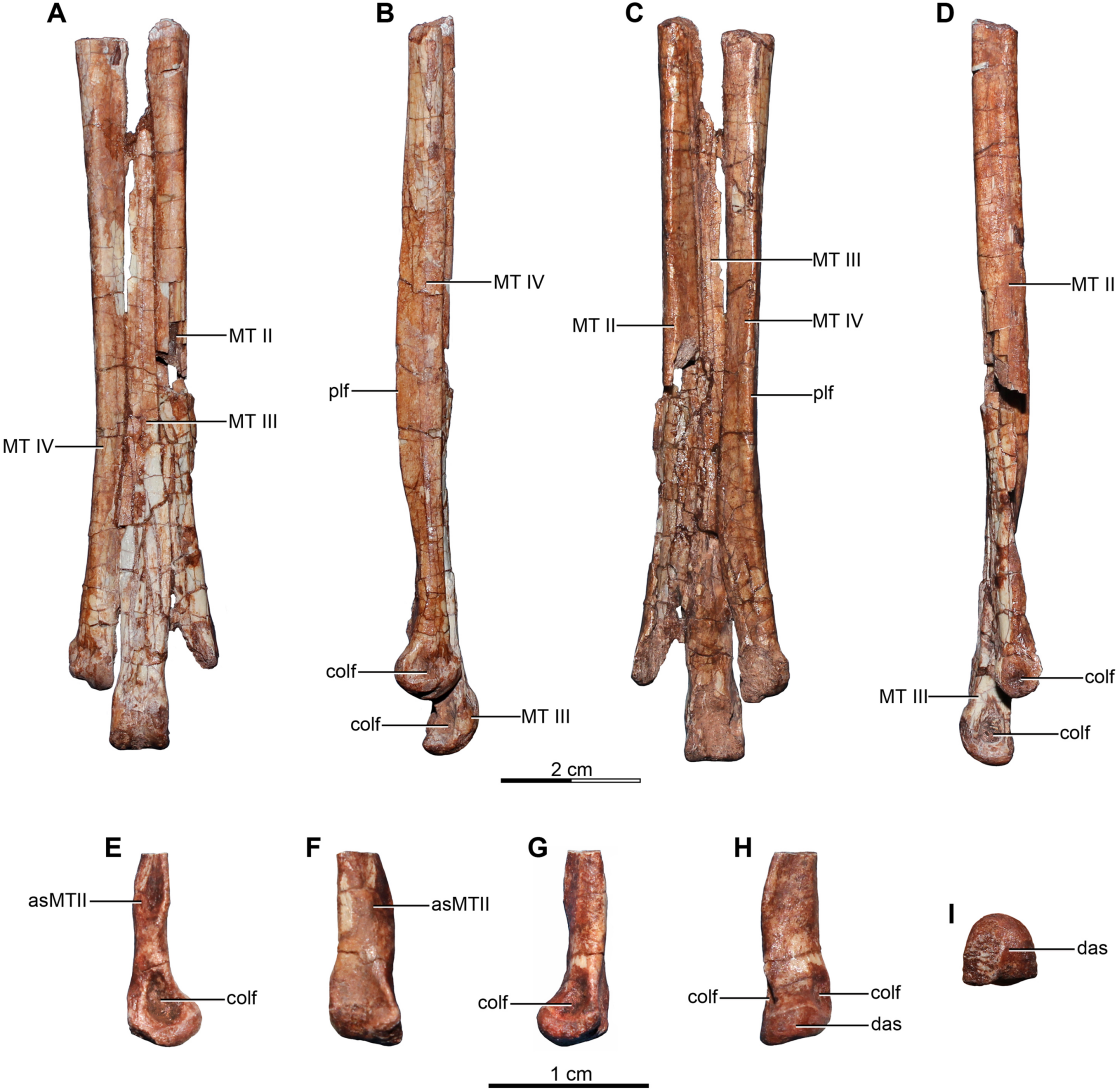
Metatarsus of the referred specimen of *Buitreraptor gonzalezorum* (MPCA 238). (A–D) Articulated right metatarsals II, III and IV, in (A) anterior, (B) lateral, (C) posterior and (D) medial view. (E–I) Right metatarsal I, in (E) lateral, (F) posterior, (G) medial, (H) anterior and (I) distal view. Scales: 2 cm for A–D, 1 cm for E–I. asMT, articular surface for the metatarsal; colf, fossa for the collateral ligament; das, distal articular surface; MT, metatarsal; plf, posterolateral flange.

**Figure 15:**
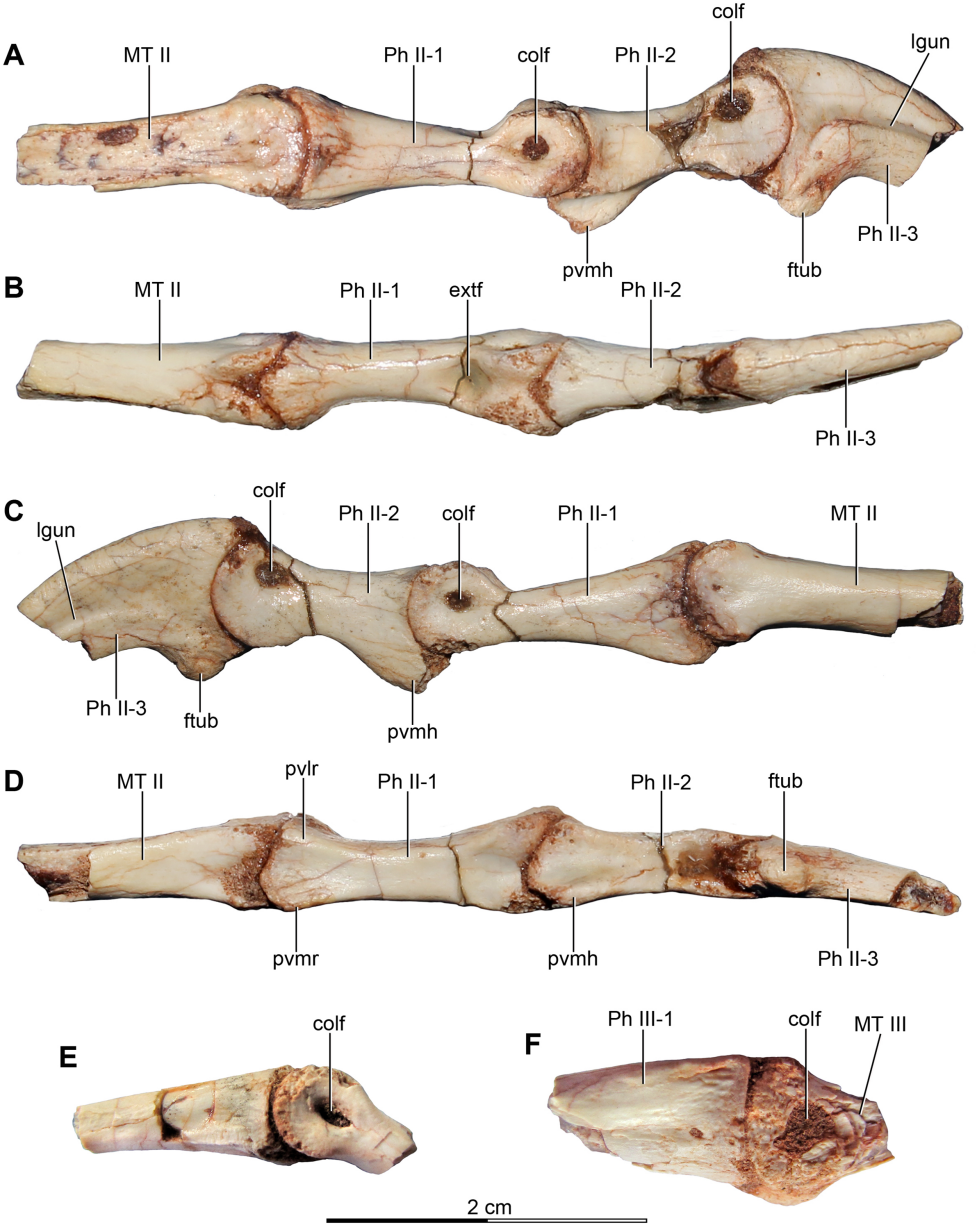
Metatarsal II and pedal phalanges of the referred specimen of *Buitreraptor gonzalezorum* (MPCA 478). (A–D) Articulated distal portion of the right metatarsal II, and pedal phalanges II-1, II-2 and II-3, in (A) lateral, (B) dorsal, (C) medial and (D) ventral view. (E) Fragmentary articulated pedal phalanges, in side view. (F) Articulated distal portion of metatarsal III and proximal portion of pedal phalanx III-1, in side view. colf, fossa for the collateral ligament; extf, extensor fossa; ftub, flexor tubercle; lgun, lateral groove of the ungual; MT, metatarsal; Ph, phalanx; pvlr, posteroventral lateral ridge; pvmh, posteroventral medial heel; pvmr, posteroventral medial ridge.

The metatarsus is preserved in several specimens, including the holotype, MPCA 238, MPCA 471-D and MPCA 478. The holotype preserves the distal parts of right metatarsals II–IV and part of left metatarsal II (Figs. 13A–13E). MPCA 471-D includes fragmentary but articulated metatarsals II-IV (Figs. 13F–13H). MPCA 238 includes the almost complete right metatarsals II-IV in articulation as well as a disarticulated right metatarsal I (Figs. 14A–14I), and MPCA 478 preserves the distal portion of metatarsal III and the articular ginglymus of metatarsal II (Figs. 15A–15D and 15F).

In MPCA 238 the right metatarsals II–IV are almost complete, missing only their proximal articulations (Figs. 14A–14D). Both MTs II and IV have similar proportions, whereas MT III shows more variation in diameter along its shaft. MT III is proximally pinched and compressed between the two flanking metatarsals, and its diameter along the proximal section is half of the diameter of the distal portion, although it remains visible in both anterior and posterior views (Figs. 14A and 14C), as is also observed in *Neuquenraptor* and *Pamparaptor* (Novas & Pol, 2005; Porfiri, Calvo & Dos Santos, 2011). In *Austroraptor* (specimen MML 220), the middle section of MT III is also constricted and the anterior surface of the distal portion is transversely expanded as in the other unenlagiines, so a similar anatomy of the metatarsus is inferred for *Austroraptor*. This morphology corresponds to a subarctometatarsal condition, and is also observed in microraptorine dromaeosaurids such as *Sinornithosaurus*, *Microraptor* and *Tianyuraptor* (Hwang et al., 2002; Xu, 2002; Zheng et al., 2010), and early diverging troodontids such as *Sinovenator*, *Mei* and *Sinornithoides* (Currie & Dong, 2001; Xu, 2002; Xu & Norell, 2004). This condition differs from the fully arctometatarsal pes of other theropods, such as tyrannosaurids (e.g., *Gorgosaurus*, Lambe, 1917; *Tyrannosaurus*, Brochu, 2003), ornithomimids (e.g., *Struthiomimus*, Osborn, 1917; *Gallimimus*, Osmólska, Roniewicz & Barsbold, 1972), alvarezsaurids (e.g., *Ceratonykus*, Alifanov & Barsbold, 2009; *Kol*, Turner, Nesbitt & Norell, 2009) and derived troodontids (*Troodon*, Russell, 1969; *Talos*, Zanno et al., 2011). Conversely, MT III of *Rahonavis* is not proximally pinched and has a comparable diameter to MT II and IV along its total length, a trait also present in *Mahakala* and more derived dromaeosaurids such as *Deinonychus* and *Velociraptor* (Ostrom, 1969; Norell & Makovicky, 1997; Turner, Pol & Norell, 2011). However, *Rahonavis* does resemble other unenlagiines in having sharp anterolateral and anteromedial edges along the distal section of MT III, which slightly overlap the shafts of the neighboring metatarsals.

The metatarsus is very elongate, especially in comparison to the femur and tibia. In MPCA 238, the length of MT III represents approximately 70% and 67% of the femoral and tibial lengths respectively (Table S1), proportions similar to those observed in MPCN-PV-598 (Novas et al., 2018). Generally, in unenlagiines the length of the metatarsus exceeds half of the length of the femur, with the metatarsus of *Buitreraptor* being proportionately the longest. These proportions are similar to those observed in *Sinornithosaurus*, *Microraptor*, and *Bambiraptor* (Xu, Wang & Wu, 1999; Burnham et al., 2000; Xu, Zhou & Wang, 2000; Hwang et al., 2002; Xu, 2002; Burnham, 2004), and in troodontids such as *Sinovenator*, *Sinornithoides* and *Saurornithoides* (Xu, 2002; Xu et al., 2002), and also in *Archaeopteryx* (Wellnhofer, 1974, 1992; Xu, 2002), in which the length of the metatarsus in comparison with that of the femur ranges between 70% and 80%. Conversely, *Rahonavis* has a comparatively short metatarsus, which represents only 40% and 55% of the tibial and femoral length respectively (Forster et al., 1998), and thus resembles the proportions observed in derived dromaeosaurids such as *Deinonychus* and *Velociraptor*. MT II is slightly shorter than the MT IV in *Buitreraptor* (Figs. 14A and 14C), as in *Neuquenraptor* and *Rahonavis*. In *Pamparaptor* MT III and IV are sub-equal in length whereas MT II is significantly shorter (Porfiri, Calvo & Dos Santos, 2011), characters that resemble those observed in some troodontids including *Troodon* (Russell, 1969). Metatarsals II and IV have comparable diameters in both *Buitreraptor* and *Neuquenraptor* (Figs. 14A and 14C), whereas in *Rahonavis* the shaft of the MT II is slightly stouter than MT IV, and in *Pamparaptor* MT II is stouter than either MT III and IV. These relative proportions differ from those of derived troodontids, in which MT IV is markedly more robust, as in *Troodon*, *Linhevenator*, *Talos* and *Gobivenator* (Russell, 1969; Xu et al., 2011; Zanno et al., 2011; Tsuihiji et al., 2014).

MT I is roughly tear-drop shaped with a tapering proximal end and a round distal articulation. The short, spike-like shaft is anteroposteriorly compressed proximally, but it expands distally (Figs. 14E–14I). The posterolateral surface of the shaft is flat, and represents the articular surface with the MT II (Figs. 14E and 14F). The distal articular end is shaped as a single, rounded condyle with an anteriorly expanded smooth surface, as is also observed in MPCN-PV-598. In this regard, the articular surface is similar to that observed in *Sinornithosaurus* (Xu, 2002) and troodontids as *Troodon* and *Talos* (Fowler et al., 2011; Zanno et al., 2011), although it differs from that of *Neuquenraptor*, *Mahakala* and derived dromaeosaurids, such as *Deinonychus*, *Velociraptor*, *Bambiraptor* and *Adasaurus*, in which MT I bears a ginglymous distal articulation with two well-defined condyles separated by a conspicuous groove (Ostrom, 1969; Norell & Makovicky, 1997; Burnham, 2004; Fowler et al., 2011; Turner, Pol & Norell, 2011). The lateral part of the articulation is more distally expanded than the medial side so that the first digit would be set at angle to the metatarsus. A shallow and short anteroposterior groove marks the posterodistal surface. The posterior surface of the distal portion is slightly concave, and the lateral and medial sides of the articulation bear collateral ligament pits with the lateral pit being the larger and deeper of the two.

MT II has a subtriangular cross section along its proximal half in MPCA 238. The anterior and medial surfaces of the shaft are slightly convex, whereas the lateral surface is flat and posteromedially inclined. Thus, the shaft has a defined posteromedial border that is slightly posteriorly projected as a plantar ridge, a trait also present in MPCN-PV-598. The distal portion of MT II is poorly preserved in MPCA 238, but it is well preserved in the right and left MT II of the holotype (Figs. 13A, 13B, 13D and 13E). This part has a more rounded cross section than the proximal part and the posterolateral border partially covers the posterior surface of the MT III. The distal articulation is ginglymoid with two well-developed condyles separated by a well-defined intercondylar groove (Figs. 13D and 13E) as in *Neuquenraptor*. The medial condyle is slightly larger and more distally extended than the lateral one. Deep collateral ligament pits are present on the lateral and medial surfaces.

MT III in MPCA 238 has an eroded proximal half and only the anterior surface is preserved. This surface is flat for most of its length, except distally where it is slightly transversely concave between the anterolateral and anteromedial edges that overlap the neighboring metatarsal shafts. MT III has a trapezoidal cross section in the distal half of the shaft, with a wide and flat anterior surface and a narrow posterior surface that is constricted and inset between the shafts of MTs II and IV. This morphology is best discerned in the holotype and in MPCA 471-D, where MT III is fractured close to the distal end revealing a transverse section (Fig. 13C). The anterior surface of the distal portion is expanded and partially covers MT II and IV, whereas the posterior surfaces of the distal portion of MT II and IV partially cover the posterior surface of MT III, as also occurs in *Neuquenraptor*, *Austroraptor* and *Pamparaptor*. MT III of *Rahonavis* slightly overlaps MT II and IV on the anterior face of the foot, whereas MT II and IV are not expanded over the posterior surface of MT III (FMNH PR 2830), as is observed in *Mahakala* (Turner, Pol & Norell, 2011). In *Buitreraptor* the shaft of MT III is also compressed dorsoventrally and defines the bottom of a deep trough between the shafts of MT II and MT IV along the plantar surface of the proximal two thirds of the metatarsus, a similar morphology to that of *Neuquenraptor* (Brissón Egli et al., 2017). The extensor sulcus on the anterior surface present in *Neuquenraptor* is not observed on MT III of *Buitreraptor*. The distal articulation of MT III of MPCA 478 is ginglymoid, as can be corroborated in the MT III of MPCN-PV-598, with both condyles similar in size and bearing well-developed collateral ligament pits. The distal articulation of MT III of MPCA 238 is poorly preserved, but two symmetrically developed condyles with a wide and shallow intercondylar groove between them are discernible (Figs. 14A and 14C). MT III of *Neuquenraptor*, *Rahonavis* and *Austroraptor* also bear a ginglymoid distal articulation, but like *Buitreraptor*, it is not as defined as in derived dromaeosaurids such as *Deinonychus* and *Velociraptor*, in which the intercondylar groove is deeply incised. In the case of *Deinonychus*, the articulation is proximally delimited by a transverse ridge (Ostrom, 1969; Norell & Makovicky, 1997, 1999). However, the ginglymoid MT III distal articulation of unenlagiines is not as weakly developed as in *Sinornithosaurus*, *Microraptor* and *Jeholornis* (Hwang et al., 2002; Xu, 2002; Zhou & Zhang, 2002). An exception is *Pamparaptor*, in which the distal articulations of both MT II and III are smooth and almost rounded rather than ginglymoid (Federico A. Gianechini, 2012, personal observation).

MT IV has a roughly triangular, anteroposteriorly compressed cross section along its proximal portion in MPCA 238. The posterior border rises to form a posterolateral crest or flange, which is best developed along the midsection of the shaft (Figs. 14B and 14C). This flange grades into the rounded shaft surface distally. The same feature is present in MPCN-PV-598. A similar posterolateral flange is also present in *Neuquenraptor*, *Pamparaptor*, *Microraptor*, *Sinornithosaurus* and the troodontids *Sinovenator*, *Sinornithoides* and *Mei* (Xu, Wang & Wu, 1999; Xu & Wang, 2000; Xu, Zhou & Wang, 2000; Hwang et al., 2002; Xu, 2002; Porfiri, Calvo & Dos Santos, 2011; Gao et al., 2012). In *Neuquenraptor* and *Pamparaptor* this crest is better developed than in *Buitreraptor* and is projected farther posteriorly in the midsection. In *Rahonavis* MT IV bears a posterolateral flange that is less defined and lower, and is laterally deflected along its distal part.

In *Buitreraptor* the posteromedial border of MT IV is medially curved and expands distally to partially cover the posterolateral surface of the shaft of MT III (Figs. 13B, 14C and 14C). The distal articulation is well-preserved in MPCA 238; it is non-ginglymoid and asymmetrical. In distal view, the medial hemicondyle is more developed and is slightly more anteriorly displaced, whereas the lateral condyle is very posteriorly displaced and its articular surface is smaller (Figs. 14A and 14C), as is also observed in MPCN-PV-598.

##### Pedal phalanges

**Figure 16:**
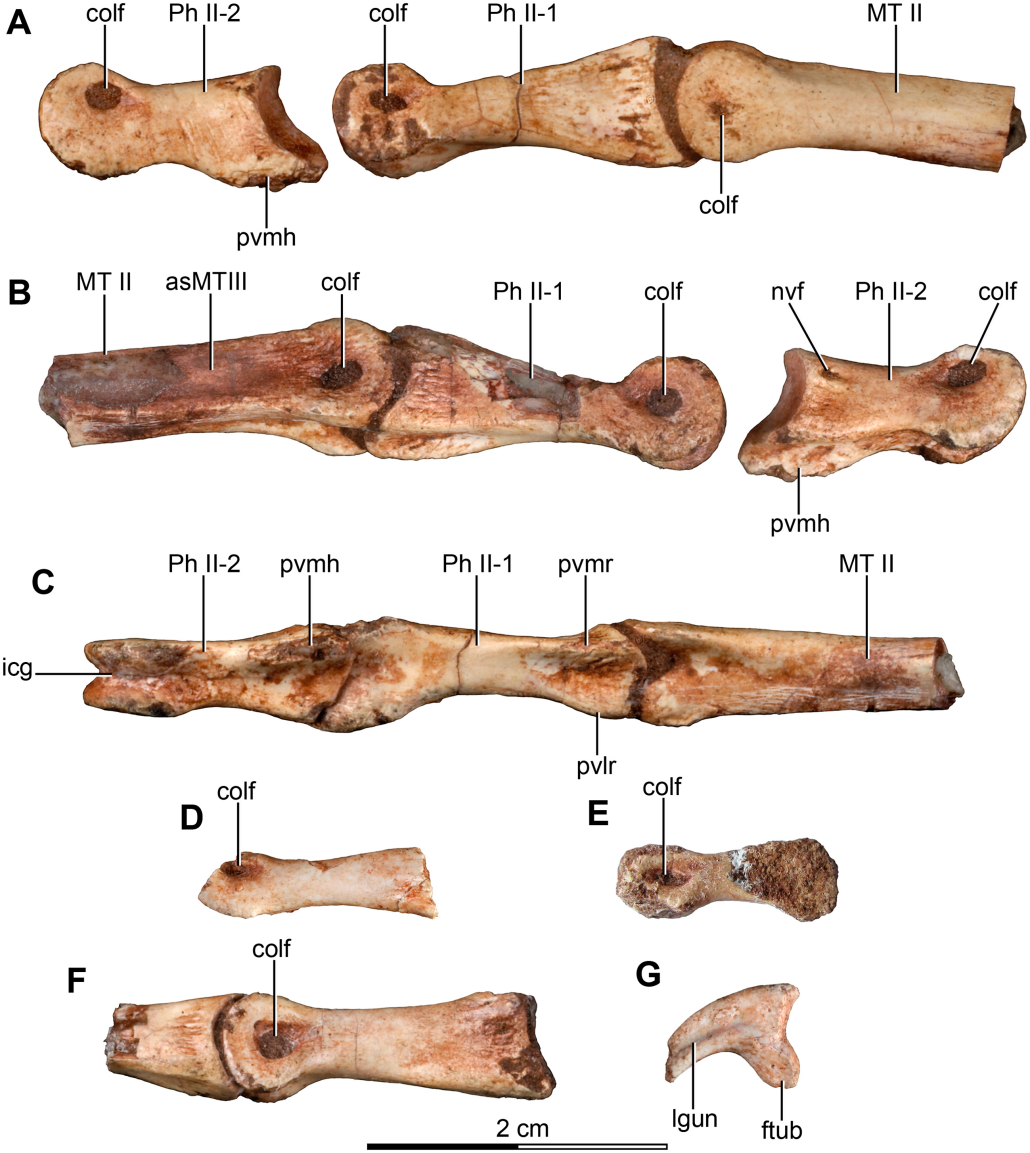
Metatarsal II and pedal phalanges of the holotype of *Buitreraptor gonzalezorum* (MPCA 245). (A–C) Right metatarsal II and pedal phalanges II-1 and II-2, in (A) medial, (B) lateral and (C) ventral view (in C the phalanx II-2 is articulated with phalanx II-1). (D) Possible pedal pre-ungual phalanx of digit IV, in side view. (E) Indeterminate pedal phalanx, in side view. (F) Possible pedal phalanx IV-1 articulated with the proximal portion of the phalanx IV-2, in side view. (G) Ungual phalanx from pedal digit I, in side view. asMT, articular surface for the metatarsal; colf, fossa for the collateral ligament; ftub, flexor tubercle; icg, intercondylar groove; lgun, lateral groove of the ungual; MT, metatarsal; nvf, neurovascular foramen; Ph, phalanx; pvlr, posteroventral lateral ridge; pvmh, posteroventral medial heel; pvmr, posteroventral medial ridge.

**Figure 17:**
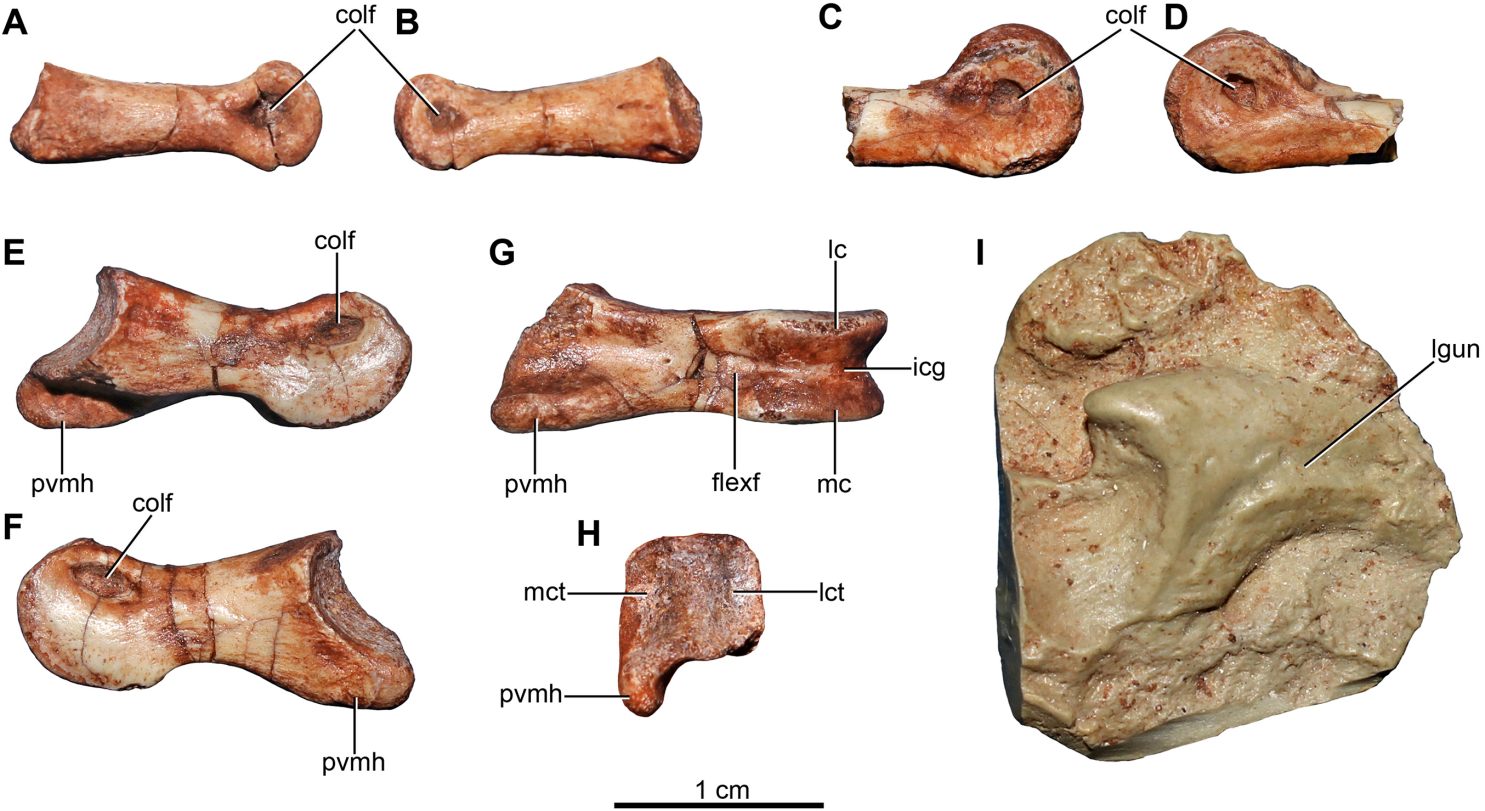
Pedal phalanges of the referred specimen of *Buitreraptor gonzalezorum* (MPCA 238). (A, B) Right pedal phalanx I-1, in (A) lateral and (B) medial view. (C, D) Distal portion of right pedal phalanx II-1, in (C) lateral and (D) medial view. (E–H) Right pedal phalanx II-2, in (E) lateral, (F) medial, (G) ventral and (H) proximal view. (I) Cast of the pedal ungual phalanx of digit II. colf, fossa for the collateral ligament; flexf, flexor fossa; icg, intercondylar groove; lc, lateral condyle; lct, lateral cotyle; lgun, lateral groove of the ungual; mc, medial condyle; mct, medial cotyle; pvmh, posteroventral medial heel.

The holotype and the specimens MPCA 238, MPCA 471-D and MPCA 478 include well-preserved pedal phalanges. MPCA 471-D includes an ungual phalanx that could correspond to that of digit III or IV (Fig. 13H). MPCA 478 comprises the right phalanges II-1, II-2 and II-3 in articulation and what is probably the proximal portion of phalanx III-1 and two other fragments (Fig. 15). The holotype includes the right phalanges II-1 and II-2 and four other phalanges corresponding to digit III or IV (Fig. 16). It also includes two disarticulated unguals, one of which is probably from the first digit whereas the other differs from the unguals of digits I and II and thus corresponds to either digit III or IV. MPCA 238 preserves phalanx I-1, the distal portion of the phalanx II-1, a complete II-2 and a cast of the ungual phalanx of the second digit obtained from a natural mold preserved in the rock (Fig. 17).

Phalanx I-1 is small when compared to the other pedal phalanges (Figs. 17A and 17B). The proximal articular surface is concave and bowl-shaped, corresponding to the ball-shaped distal articular surface of MT I. From the proximal articulation the shaft decreases in diameter reaching its minimal dorsoventral depth just proximal to the distal articulation. The distal articulation is ginglymoid and bears two well-developed condyles, the medial one of which is smaller. Two round collateral ligament pits are present. The general morphology of this phalanx is very similar to that observed in *Austroraptor* and *Neuquenraptor*.

Phalanx II-1 is articulated with MT II in the holotype (Figs. 13A, 13B and 16A–16C) and it is articulated with MT II and phalanx II-1 in MPCA 478 (Figs. 15A–15D). The proximal part is transversely compressed and is taller than the distal portion. A deep extensor fossa is observed on the dorsal surface adjacent to the distal condyles, which rise up sharply from the dorsal surface of the shaft in lateral view. The dorsal surface narrows to a low, longitudinal ridge extending from the proximal border to the medial distal condyle. The ventral surface is marked by lateral and medial ridges flanking a midline sulcus, with the medial ridge waning before reaching the distal articulation (Figs. 15D and 16C). These ventral ridges are also present in MPCN-PV-598 and on phalanx II-1 of other unenlagiines such as *Neuquenraptor*, *Austroraptor*, *U. paynemili* and *Rahonavis* (Federico A. Gianechini, 2012, personal observation), and other dromaeosaurids including *Velociraptor* (Norell & Makovicky, 1997). The poor preservation of this phalanx in *Pamparaptor* precludes confirmation of either the presence or absence of these structures. The proximolateral and distolateral portions of the phalanx are slightly expanded, such that the phalanx has a markedly concave lateral outline in ventral view. The medial surface is straighter, however. The distal end of the phalanx is slightly laterally deflected and the distal articulation is ginglymoid with the lateral ginglymus slightly wider than the medial one. The articular surface of the condyles is strongly proximodorsally expanded, with the ginglymous articulation extending far onto the dorsal aspect of the phalanx. The range of motion of phalanx II-2 would have extended far dorsally and proximally allowing hyper-extension at this joint as in other dromaeosaurids. The maximum width across the distal articulation is slightly greater than the maximum width of the proximal articulation, a character also observed in *Neuquenraptor*, although not present in Laurasian dromaeosaurids such as *Velociraptor*, *Deinonychus* and *Dromaeosaurus* (Colbert & Russell, 1969; Ostrom, 1969; Norell & Makovicky, 1997; Fowler et al., 2011). There is a collateral ligament pit associated with each hemicondyle, and these are slightly dorsally displaced rather than being centered on the lateral faces of the distal ginglymus.

Phalanx II-2 is around 80% of the length of phalanx II-1 (MPCA 245: 81%; MPCA 238: 77%; MPCA 478: 76%; Table S1), a similar proportion to that observed in *U. paynemili* (75%) and *Austroraptor* (MML 220: 80%). On the other hand, in *Neuquenraptor* and *Pamparaptor* both phalanges are sub-equal in length (95% in both taxa), whereas *Rahonavis* is similar to dromaeosaurids such as *Velociraptor* and *Deinonychus* (Ostrom, 1969; Norell & Makovicky, 1997, 1999) in having a ratio of 90%. This ratio is significantly smaller in some troodontids, such as *Linhevenator* and *Gobivenator* (Xu et al., 2011; Tsuihiji et al., 2014). Phalanx II-2 of *Buitreraptor* has a general morphology similar to that of dromaeosaurids such as *Microraptor*, *Bambiraptor*, *Hesperonychus*, *Dromaeosaurus*, *Velociraptor* and *Deinonychus* (Matthew & Brown, 1922; Colbert & Russell, 1969; Ostrom, 1969; Norell & Makovicky, 1997, 1999; Hwang et al., 2002; Xu, 2002; Burnham, 2004; Longrich & Currie, 2009), i.e. with a proximoventrally projected “heel,” a short shaft that is constricted at midlength, and a narrow distal articulation with two well-developed and ventrally extended distal hemicondyles (Figs. 15A–15D, 16A–16C and 17E–17H). However, the proximoventral “heel” is relatively shorter and asymmetrical (i.e., it is medially offset), as in other unenlagiines, whereas in dromaeosaurids such as *Dromaeosaurus*, *Velociraptor*, *Deinonychus* and *Saurornitholestes* (Matthew & Brown, 1922; Colbert & Russell, 1969; Ostrom, 1969; Currie, 1995; Norell & Makovicky, 1997; Longrich & Currie, 2009), it is longer and more symmetrical and the intercondylar ridge of the proximal articular facet extends onto it. Particularly in *Buitreraptor*, the proximoventral heel is markedly medially offset, and is ventrally projected and transversely compressed, so that it acquires a ridge-like shape (Figs. 15A, 15C, 15D, 16A–16C and 17E–17H), whereas the remainder of the proximoventral surface of the phalanx is comparatively flat in proximal view. *Rahonavis* shows a very similar morphology of the proximal heel, although it is less medially displaced than in *Buitreraptor* and, moreover, the proximoventral end of the phalanx and the heel are scarcely posteriorly projected when viewed laterally with a notch separating the posterior apex of the heel from the posterior apex of the phalanx (Federico A. Gianechini, 2012, personal observation). In contrast, *Neuquenraptor* and *U. paynemili* have a morphology closer to that of Laurasian microraptorine dromaeosaurids such as *Hesperonychus* (Longrich & Currie, 2009), which has a medially displaced heel although not as transversely compressed and triangular in proximal view. The dorsoventral constriction of the phalangeal shaft at midlength in *Buitreraptor* is slightly more marked than in *Microraptor*, *Sinornithosaurus* and *Graciliraptor* (Xu, Wang & Wu, 1999; Xu, Zhou & Wang, 2000; Hwang et al., 2002; Xu, 2002), but not as marked as in more derived dromaeosaurids (Matthew & Brown, 1922; Colbert & Russell, 1969; Ostrom, 1969; Currie, 1995; Norell & Makovicky, 1997; Longrich & Currie, 2009). Two, small and elliptical collateral ligament pits are present and both are strongly dorsally displaced. The dorsoventral height of the distal articular surface is similar to that of the proximal articular surface (Figs. 15A, 15C, 16A, 16B and 17E, 17F), as also seen in *U. paynemili*, *Neuquenraptor*, *Austroraptor*, *Rahonavis* (Forster et al., 1998; Calvo, Porfiri & Kellner, 2004; Novas & Pol, 2005; Novas et al., 2009; Currie & Paulina Carabajal, 2012), and other dromaeosaurids like *Microraptor*, *Hesperonychus*, *Deinonychus* and *Velociraptor* (Ostrom, 1969; Norell & Makovicky, 1997, 1999; Hwang et al., 2002; Longrich & Currie, 2009). This differs from the condition observed in troodontids where the proximal surface is markedly more expanded dorsoventrally than the distal surface, as in, for example, *Talos* (Zanno et al., 2011).

The ungual phalanx of digit II is comparatively large with respect to the remaining pedal unguals and in general, it exhibits a similar morphology to that of other dromaeosaurids and troodontids (e.g., *Velociraptor*, *Deinonychus*, *Saurornitholestes*, *Sinornithosaurus*, *Microraptor*, *Troodon*, *Saurornithoides* and *Rahonavis*). It is strongly recurved and transversely compressed (Figs. 15A–15D and 17I), the lateral surface is slightly convex and the medial one is mainly flat so that the transverse cross-section is D-shaped and the trenchant ventral edge is medially displaced, a trait also present in *Mahakala* and *Velociraptor* (Norell & Makovicky, 1997; Turner, Pol & Norell, 2011). This same morphology of the ungual is observed in MPCN-PV-598. A large, convex flexor tubercle is located proximoventrally adjacent to the ventral end of the proximal articulation. The lateral and medial surfaces each bear a longitudinal groove with the lateral one being deeper and more marked. The medial groove is ventrally displaced relative to the lateral one (Figs. 15A and 15C), a condition also present in MPCN-PV-598. This asymmetry in the location of the lateral and medial grooves is shared with *Neuquenraptor*, *Mahakala* and *Hesperonychus* (Longrich & Currie, 2009; Turner, Pol & Norell, 2011), but is not as prominent as in *Saurornitholestes*, *Velociraptor* and *Deinonychus* (Ostrom, 1969; Norell & Makovicky, 1997; Longrich & Currie, 2009).

MPCA 245 also includes two fragmentary articulated phalanges, which correspond to either digit III or IV, because they differ from the phalanges of either digit I or II (Fig. 16F). One of them is almost complete, and has a dorsoventrally deep poorly preserved proximal articulation, a shaft that is mostly straight with a slightly concave ventral surface, and a ginglymoid distal articulation. The morphology of this phalanx resembles that of the phalanx IV-1 of MPCN-PV-598. The proximal half of phalanx IV-2 is articulated with it but too poorly preserved to yield much anatomical information.

Two other fragmentary phalanges were preserved in the holotype, both with eroded proximal articulations and partially preserved ginglymoid distal articulations (Figs. 16D and 16E). One of these phalanges has slightly dorsally displaced collateral ligament pits, suggesting it is a pre-ungual phalanx (Fig. 16D). These probably correspond to phalanges IV-3 and IV-4, based on the morphology of digit IV phalanges in MPCN-PV-598, which are short and unlike the elongated phalanges of the third digit.

The ungual phalanx of the first digit of MPCA 245 is transversely compressed and slightly curved (Fig. 16G). The proximal articulation is eroded, but a well-developed flexor tubercle was preserved with a ventral extent that corresponds to approximately 50% of the height of the adjacent articular surface. Lateral and medial longitudinal grooves extend from close to the base of the flexor tubercle distally, and one of them is more marked. The remaining ungual phalanx of MPCA 245 likely belongs to either digit III or IV, since it differs from the unguals of digits I and II. It is gently curved and transversely compressed, and a pronounced flexor tubercle is present proximoventrally. Deep longitudinal claw sheath grooves are present on the lateral and medial surfaces, and they are asymmetrically arranged with one of them dorsally displaced above the other. MPCA 471-D includes an ungual phalanx very similar to this element, except that the claw sheath grooves are less prominent (Fig. 13H).

The remaining phalanges of MPCA 478 repesent the proximal portion of phalanx III-1 articulated with MT III and two fragmentary phalanges probably from digit III or IV. The proximal portion of phalanx III-1 is dorsoventrally expanded whereas the midsection decreases in its transverse diameter (Fig. 15F). The other two fragmentary phalanges are articulated with each other. The more proximal one only preserves the distal half exhibiting a dorsal extensor fossa, whereas the other phalanx preserves the proximal half, with a dorsoventrally expanded proximal portion (Fig. 15E).

## Bone Histology and Ontogenetic Stage

In order to assess the minimun ages and ontogenetic growth stages of the studied specimens, histological thin sections were made from the humerus and tibia of the holotype (MPCA 245), and from other bones of referred specimens, specifically the tibia of MPCA 238 and the metatarsals of MPCA 471-D. Samples for sectioning were obtained from the mid-shafts of these elements. Preparation of the histological sections was carried out at the Departamento de Geología de la Universidad Nacional de San Luis (Argentina). The slices were prepared using standard methods outlined by Chinsamy & Raath (1992) and studied using a petrographic polarizing microscope (Nikon E200 pol). Nomenclature and definitions of structures used in this study follow Francillon-Vieillot et al. (1990) and Chinsamy-Turan (2005).

**Figure 18:**
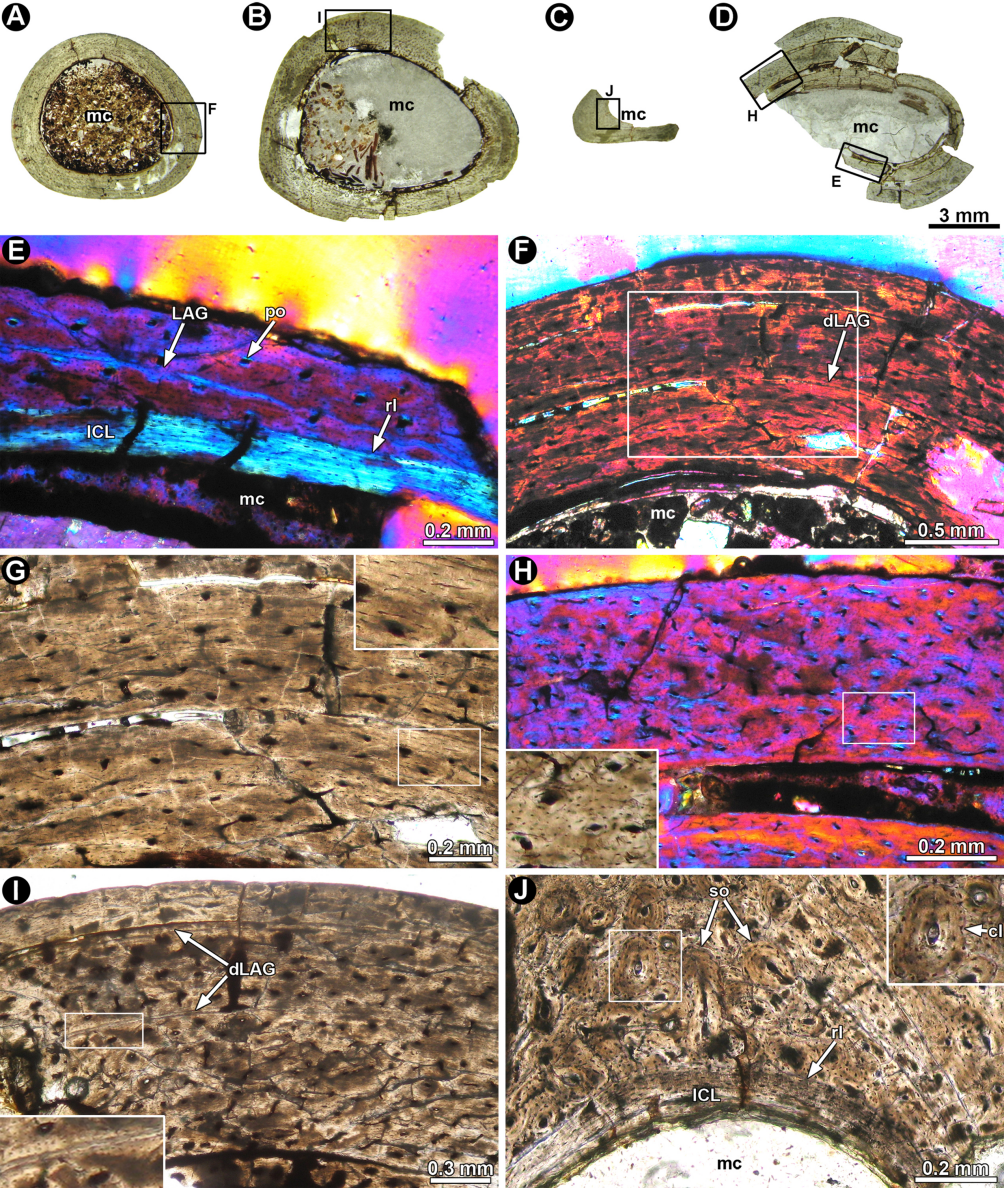
Long bone histology of *Buitreraptor gonzalezorum*. (A–D) Complete cross section of selected bones corresponding to specimens MPCA 245 (A, B), MPCA 471-D (C) and MPCA 238 (D, E), including humerus (A), tibiae (B, D) and metatarsal (C); all the elements to the same scale. (E) Detail of the inner circumferential layer around the medullary cavity (small box inset in D). (F) General view of the compact bone of the humerus (box inset in A). Note the slight degree of birefringence of the compacta. (G) Detailed view of the cortical bone (box inset in F) showing the predominance of longitudinally oriented vascular spaces; a detailed view of the bone cell lacunae is showed in the upper right corner. (H) Cortical bone composed of fibrolamellar tissue (large box inset in D); a detailed view of the bone cell lacunae is showed in the lower left corner. (I) Enlarged view of the cortical bone showing two double LAGs; a detailed view of one of the double LAGs is showed in the lower left corner. (J) Inner cortex of the metatarsal (box inset in C) showing an ICL and secondary osteons; a detailed view of one secondary osteon is showed in the upper right corner. cl, cementing line; dLAG, double line of arrested growth; ICL, inner circumferential layer; mc, medullary cavity; po, primary osteon; rl, resorption line; so, secondary osteons.

The cortical region of the shaft of all sampled elements is composed of compact bone and borders a large marrow cavity (Figs. 18A–18D). Except for the tibia of MPCA 245, the perimedullary region of the bones of the other specimens is lined by a layer of endosteally deposited, avascular lamellar bone (the inner circumferential layer, ICL) containing flattened osteocyte lacunae (Figs. 18E–18H and 18J). The cortical bone tissue consists almost entirely of well vascularized primary bone. Due to diagenetic alteration of the tissue, the organization of the intrinsic fibers is difficult to assess with certainty. Nevertheless, this parameter can be inferred on the basis of the shape and distribution of the osteocyte lacunae and the optical properties of the best-preserved areas of the bone. The highest degree of fibrilar organization is observed in the humerus of MPCA 245 (Fig. 18F), in which osteocyte lacunae are strongly flattened and a high degree of birefringence is observed in several areas, resembling a typical parallel fibred matrix. The tibia of the same individual shows a more disorganized pattern in general terms, although some degree of birefringence is still present (Fig. 18I). Osteocyte lacunae are rounded or oblate and they exhibit a more disorganized pattern of distribution and orientation in this element. The intrinsic fiber organization is strongly variable in the metatarsal of MPCA 471 D, ranging from parallel fibered to woven fibered bone (Fig. 18J). Finally, the intrinsic fibers of the tibia MPCA 238 appears to be faintly organized, with rounded to slightly elongated ostecyte lacunae. Only the metatarsal of MPCA 471-D exhibits Sharpey’s fibers in the compacta.

Lines of arrested growths (LAGs) and, in some instances, annuli, are recorded in all the samples. LAGs are simple or double. Whereas three LAGs are preserved in both the humerus and tibia of MPCA 245, four are recorded in the metatarsal of MPCA 471-D. Five LAGs are observed in the tibia of MPCA 238.

Vascular spaces are simple or lined by a thin layer of lamellar bone (i.e., primary osteons). Their arrangement is mostly longitudinal, but variation on this regard occurs. For example, in the humerus of MPCA 245, some radial, oblique and circumferential anastomoses are observed. The tibia of the same individual is dominated by longitudinal and oblique canals. The density of vascular spaces is also variable among different elements and within a single section. In this regard, whereas both the humerus of MPCA 245 and the tibia of MPCA 238 exhibit a roughly equivalent, moderate degree of vascularization, the density of vascular spaces is more pronounced in the tibia of MPCA 245. The density of vascular spaces decreases toward the outer cortex, particularly peripheral to the outermost LAG. Secondary osteons are only recorded in the metatarsal of MPCA 471 D, in which they are clustered in the perimedullary region of the cortex.

The absence of an Outer Circumferential Layer (i.e., peripheral band of lamellar or parallel fibered bone with closely packed growth lines) in the external cortex of the sampled bones of MPCA 245, MPCA 238 and MPCA 471 D, indicates that these specimens were subadults (i.e., not somatically mature) at time of death (Chinsamy-Turan, 2005). The wide spacing between growth marks and the absence of secondary remodeling (except for in a single bone) also suggest that the specimens were actively growing individuals. The change in the vascular density in the outer cortex of MPCA 245 is possibly related to a slight reduction in the growth rate of the elements. The reduction of the growth rate in the long bones of dinosaurs and other vertebrates has been previously interpreted as a signal of the attainment of sexual maturity in individuals (Chinsamy-Turan, 2005). Nevertheless, such reduction has been inferred from the change in the intrinsic fiber organization of primary bone (from woven to parallel fibered) and/or from a clear reduction in the space between successive growth marks (Sander, 2000; Cerda & Chinsamy, 2012). Such abrupt change between the arrangement of the intrinsic fiber arrangement and growth marks spacing is not evident in sampled bones of *Buitreraptor*, which suggest that the individuals were likely not sexually mature at the time of death.

Lines of arrested growth counts in the tibia, which is the only bone sampled for more than one specimen, indicates that MPCA 238 was older than MPCA 245 despite being smaller. LAG counts along with other histological traits show that MPCA 245 and MPCA 238 were at least in their fourth or fifth year of life at the time of death, respectively. The large size of the medullary cavity in the long bones of both individuals, which may have destroyed part of the growth record as it expanded, implies that the specimens may well have been older. Despite the subadult growth stage of these specimens as inferred from histology, they present anatomical characters often interpreted as osteological markers of somatic maturity, such as complete fusion of the neurocentral sutures in the holotype. With respect to the observed differences between the specimens we find the histological evidence to be consistent with osteological growth markers. For example, in the holotype the last sacral vertebra is not fully fused to the rest of the sacrum indicating a younger ontogenetic stage than MPCA 238, in which the last sacral vertebra is firmly fused to the remaining sacrals. This character may indicate a possible pattern of osteological fusion correlated with ontogenetic development in this taxon.

Comparison to the specimen MPCN-PV-598, for which bone histology has been recently studied by Novas et al. (2018), the higher number of LAGs (8) in that individual indicates an older age in comparison with MPCA 245 and MPCA 238. This is further evidence indicating that the fusion and number of sacral vertebrae increased with the age of the individuals, since six sacrals were reported in MPCN-PV-598. Based on the reduction in the spacing between the outermost LAGs and the increase in the degree of intrinsic fiber arrangement, Novas et al. (2018) proposed that MPCN-PV-598 had reached sexual maturity at the time of death. Although the absence of an OCL indicates a sub-adult condition in this especimen, neurocentral sutures are clearly fused as in MPCA 245 (Novas et al., 2018). In terms of the other histological features we examined (i.e., arrangement of intrinsic fibers, degree of vascularization), MPCN-PV-598 is very similar to the specimens sampled here.

## Phylogenetic Analysis

In the original paper describing *Buitreraptor*, Makovicky, Apesteguía & Agnolín (2005) analyzed the phylogenetic position of this taxon and recovered it as a member of a Gondwanan group of dromaeosaurids, which they dubbed Unenlagiinae. In their analysis, *Buitreraptor* was recovered as the earliest diverging member of this clade, which also included *Rahonavis* and *Unenlagia* (which they considered synonymous with *Neuquenraptor*). Subsequent analyses conducted by diverse authors (Norell et al., 2006; Turner et al., 2007; Hu et al., 2009; Novas et al., 2009; Senter et al., 2012; Brusatte et al., 2013) continued to recover *Buitreraptor* as an unenlagiine, though with different relationships within that clade. On the other hand, Agnolín & Novas (2011, 2013) proposed that the traits observed in the unenlagiines demonstrate affinities with Avialae. They performed a phylogenetic analysis where Unenlagiinae is recovered outside of Deinonychosauria and within Avialae, as a distinct family, Unenlagiidae, as originally proposed by Bonaparte (1999).

Recently, we performed a phylogenetic analysis to evaluate the distribution of cranial characters in this taxon (Gianechini, Makovicky & Apesteguía, 2017). In this study, we conduct a new analysis in order to evaluate the phylogenetic relationships of dromaeosaurids and the distribution of characters in paravian evolution, focusing on the results and phylogenetic implications for postcranial characters. The cladistic data set we use for our analysis is based on the most recent versions of the TWiG (Theropod Working Group) data set (Brusatte et al., 2014), which consists of 152 taxa and 853 phenotypic characters. We added new characters proposed by Gianechini, Makovicky & Apesteguía (2017), and other previous authors (Rauhut, 2003; Hu et al., 2009; Novas et al., 2009; Foth, Tischlinger & Rauhut, 2014; Grellet-Tinner & Makovicky, 2006) resulting in a complete character list including 884 characters (see Supplemental Information).

**Figure 19:**
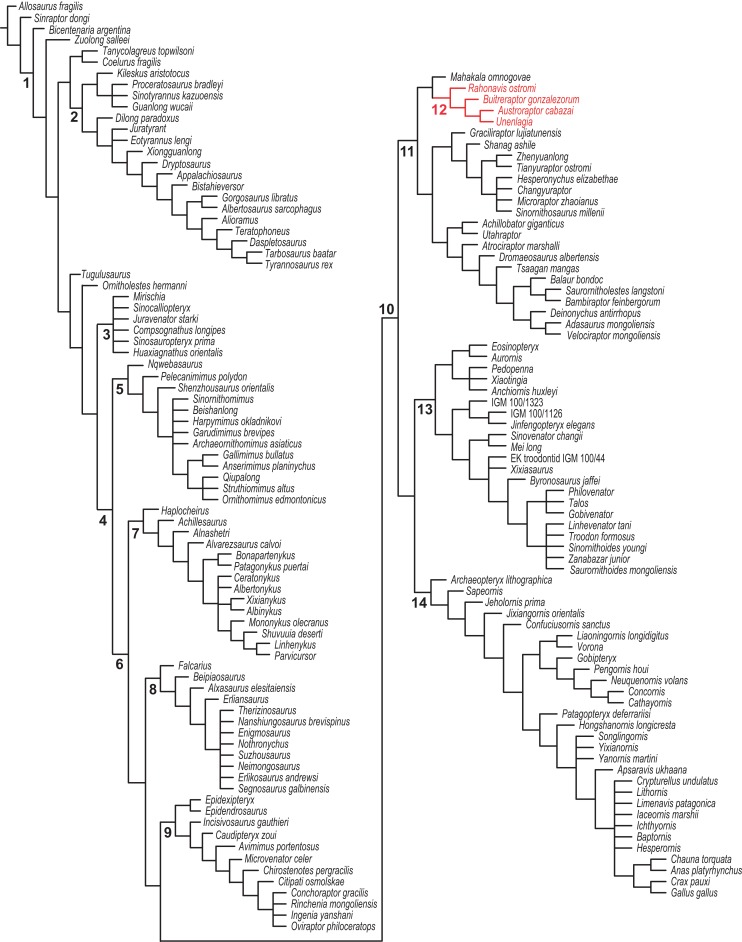
Reduced consensus tree of the MPTs obtained from the phylogenetic analysis, after the pruning of *Kinnareemimus*, *Pamparaptor* and *Pyroraptor*. Numbers correspond to the different clades recognized: 1, Coelurosauria; 2, Tyrannosauroidea; 3, Compsognathidae; 4, Maniraptoriformes; 5, Ornithomimosauria; 6, Maniraptora; 7, Alvarezsauroidea; 8, Therizinosauria; 9, Oviraptorosauria; 10, Paraves; 11, Dromaeosauridae; 12, Unenlagiinae; 13, Troodontidae; 14, Avialae.

A total of eight recently described taxa were also added, so that the complete data set now includes 160 taxa. We followed the precedent of previous authors (Makovicky, Apesteguía & Agnolín, 2005; Turner, Makovicky & Norell, 2012; Agnolín & Novas, 2013) in combining *N. argentinus*, *U. comahuensis* and *U. paynemili* as a single operational taxonomic unit (OTU) named “*Unenlagia*.” The phylogenetic analysis was carried out using TNT v. 1.5-beta (Goloboff, Farris & Nixon, 2008) with equally weighted characters. We initially used the same character ordering scheme proposed by Brusatte et al. (2014), but analyses of the ordered dataset yielded poorly resolved results and a consensus tree with numerous polytomies (see Supplemental Information). We therefore conducted a second analysis in which we treated all characters as non-additive. A heuristic search for the most parsimonious topologies was conducted by performing 1,000 replicates of Wagner trees (using random addition sequences, RAS) followed by TBR branch swapping (holding 10 trees per replicate). Zero length branches were collapsed during the analysis (rule 1 of Coddington & Scharff, 1994). The search resulted in 570 most parsimonious trees (MPTs) of 3,556 steps, found 58 times out of the 1,000 replications. A subsequent round of TBR branch swapping on these 570 trees finally found more than 999,999 MPTs. The strict consensus showed a massive polytomy including ornithomimosaurs, oviraptorosaurs, therizinosaurs and alvarezsaurs (Fig. S1). The clade Paraves was recovered, although the internal relationships present substantial differences with respect to previous studies (Turner, Makovicky & Norell, 2012; Han et al., 2014; Lü & Brusatte, 2015), because Avialae was recovered as the sister clade to Troodontidae, forming a clade that in turn is sister to Dromaeosauridae (Fig. S1). Thus, we did not recover a monophyletic Deinonychosauria. A polytomy is recovered near the base of Dromaeosauridae, which includes the Unenlagiinae and microraptorines, *Mahakala*, *Hesperonychus*, *Pyroraptor*, and *Shanag*. Three taxa were identified as “wildcards” and were pruned from the MPTs, specifically *Kinnareemimus*, *Pyroraptor* and *Pamparaptor*. After pruning these taxa, the ornithomimosaurs, oviraptorosaurs, therizinosaurs and alvarezsaurs were recovered as monophyletic clades and the basal polytomy in Dromaeosauridae is also resolved (Fig. 19). Within Dromaeosauridae most of the Laurasian taxa are grouped in the Microraptorinae and its sister clade of remaining Late Cretaceous Laurasian dromaeosaurids. The Gondwanan taxa together with *Mahakala* were recovered as a monophyletic clade, with *Mahakala* as the earliest diverging member. This clade corresponds to Unenlagiinae. The internal relationships of Unenlagiinae, excluding *Mahakala*, are the same as those recovered by some previous studies, i.e., with *Rahonavis* as the earliest diverging taxon and *Buitreraptor* as sister to the group comprising the larger *Austroraptor* and *Unenlagia*.

Two unambiguous synapomorphies support Unenlagiinae comprising *Mahakala* and the Gondwanan taxa (Supplementary Data), including the presence of six sacral vertebrae, and an astragalus and calcaneum fused to each other but not to the tibia. These traits are known to vary ontogenetically, however, as shown for *Buitreraptor* above, as well as for other paravians (Norell & Makovicky, 1999) and it is therefore likely their optimization is affected by the ontogenetic stages of the known specimens of taxa that surround this node. The core Unenlagiinae clade (i.e., excluding *Mahakala*) is supported by three synapomorphies, all of which refer to postcranial characters. Two of these were already recovered in previous analyses (Turner, Makovicky & Norell, 2012; Gianechini, Makovicky & Apesteguía, 2017) and are the presence of a reduced supracetabular crest on the ilium, and a concave dorsal edge of the postacetabular process. The remaining synapomorphy recovered here is a preacetabular process of the ilium markedly longer than the postacetabular process.

Our analysis also recovered two postcranial characters that diagnose *Buitreraptor* in addition to previously identified autapomorphies (see Supplementary Data): anterior cervical centra extending beyond the posterior end of the neural arch; and the dorsal rim of the ilium above the acetabulum strongly everted, so the lateral surface of the iliac blade can be observed in ventral view. Beyond these features there are other character combinations that diagnose *Buitreraptor*, some of which are unique to this taxon, such as the presence of ridges on the lateroventral surfaces of the centra of the eighth and ninth cervical vertebrae that end posteriorly as small tubercles; pneumatic foramina present only in the first and second dorsals; presence of lateral accessory ridges on the lateral surfaces of posterior caudal centra; a scapular blade that is dorsoventrally expanded at mid-length; and a pneumatic furcula with two pneumatic foramina on the ventral surface flanking the hypocleideum.

## Discussion

*Buitreraptor* has several postcranial characters that support its assignment to Dromaeosauridae, such as a second pedal digit with a penultimate phalanx modified for hyper-extension and a strongly curved ungual that is significantly larger than that of digit III; parapophyses of the dorsal vertebrae distinctly projected on pedicels; a bicipital scar on the ulna developed as a slightly raised ridge; an ischium with a longitudinal ridge subdividing the lateral surface into anterior and posterior parts; and a ginglymoid distal end on metatarsal II. The amount of information provided by the several known specimens of this taxon, and in particular the nearly complete holotype, allows a greater understanding of the anatomy of the South American dromaeosaurids and recognition of the characters that support their monophyly. On the other hand, *Buitreraptor* also exhibits characters that distinguish it from other dromaeosaurids and coelurosaurs. Some of these traits also distinguish it from other unenlagiines, although given the incompleteness of those taxa, it is possible that future discoveries will recover some of these features as shared derived traits among some or all unenlagiines.

Among the characters differentiating *Buitreraptor* from other unenlagiines is the lack of true pleurocoels in the dorsal vertebrae. As described above, *Unenlagia*, *Rahonavis* and *Austroraptor* have well-developed pleurocoels in the centra of the dorsal vertebrae. Moreover, this trait is present throughout the dorsal series, a condition observed in some other theropods such as basal tetanurans (e.g., *Torvosaurus*, Britt, 1991; *Neovenator*, Brusatte, Benson & Hutt, 2008), tyrannosaurids (e.g., *Tyrannosaurus*, Brochu, 2003), oviraptorosaurs more derived than *Caudipteryx*, and late diverging therizinosaurs (Zanno, 2010). On the other hand, the lack of pleurocoels on most or all dorsals is observed in basal tyrannosauroids (Xu et al., 2006), troodontids such as *Sinovenator* (Xu, 2002; Makovicky & Norell, 2004), all ornithomimosaurs (Makovicky, Kobayashi & Currie, 2004), alvarezsaurids including *Mononykus* and *Patagonykus* (Perle et al., 1994; Novas, 1997), and even *Archaeopteryx* (Britt et al., 1998), a distribution that has been termed the “common pattern” by O’Connor & Claessens (2005). Among dromaeosaurids, some taxa such as *Microraptor* and *Sinornithosaurus* (Hwang et al., 2002; Xu, 2002) lack pneumatic foramina on dorsal centra, whereas other taxa have large pleurocoels in all dorsal vertebrae, like in *Deinonychus* and *Saurornitholestes* (Ostrom, 1969; Rauhut, 2003). Thus, the condition observed in *Buitreraptor* is similar to that of some basal dromaeosaurids, such as microraptorines, while other unenlagiines resemble more derived dromaeosaurids with well-developed pleurocoels throughout the dorsal series.

The strong lateral curvature of the dorsal rim of the ilium is another trait unique to *Buitreraptor*. Although a curvature of the ilia blade is observed in some other taxa (e.g., *Mahakala*), this eversion of the ilium is significantly more developed in *Buitreraptor*. This feature is likely related to reorientation of the lines of action of the pelvic muscles. However, understanding the impacts of this trait on musculature and functional anatomy of the hindlimbs are beyond the scope of this work.

Another trait likely related to muscle insertion is the presence of tubercles on the last cervical vertebrae. These striking tubercles likely represent additional points of muscular insertion related to neck mobility. Although posterior neck vertebrae of *Austroraptor* also have sharp ridges along the ventrolateral corners of the centra (Peter J. Makovicky, 2008, personal observation), they do not terminate in discrete tubercles as in *Buitreraptor*.

A notable feature is the difference in sacral fusion patterns between the holotype and MPCA 238. As explained in the description, the last sacral vertebra is fused to the penultimate vertebra in MPCA 238, whereas they remain separate in the holotype. Based on our histological study of the bones of these specimens, we infer that this difference is related to their different ontogenetic stages at death. MPCA 238 shows a bone histology that indicates greater maturity than the holotype, which is consistent with the late fusion of the sacral vertebrae in the ontogeny. Furthermore, MPCN-PV-598, which represents an older ontogenetic stage (Novas et al., 2018), has six sacral vertebrae and thus one more than MPCA 245 and MPCA 238. This confirms that some osteological age markers arise late in ontogeny (Griffin & Nesbitt, 2016) in specimens that otherwise appear mature, stressing the need for caution in the interpretation of ontogenetically variable traits, especially in taxa represented by a single specimen, as is common for dinosaurs. As noted above, all unambiguous synapomorphies we recover in support of grouping *Mahakala* with Unenlagiinae belong in this category.

### Phylogenetic implications of the synapomorphies of Unenlagiinae and other significant characters for paravian evolution

The three synapomorphies that support core Unenlagiinae (i.e., excluding *Mahakala*) monophyly all pertain to the pelvic girdle, a portion of the skeleton widely preserved across unenlagiines, and which present a particular morphology distinguishing them from other groups of paravian theropods. Below, we present a discussion of the phylogenetic implications of these characters and other select postcranial traits that are important for understanding paravian evolution.

**Figure 20:**
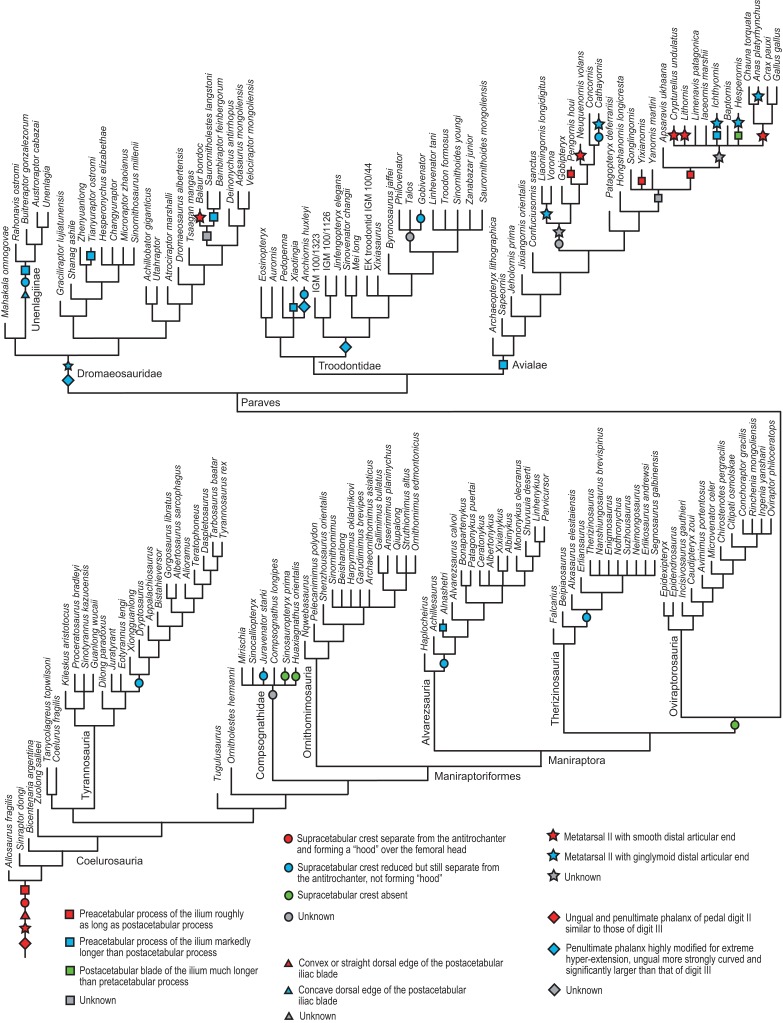
Optimization of the morphological characters discussed in the text.

**Supracetabular crest reduced:** A low but distinct supracetabular crest on the ilium is observed in *Buitreraptor*, *Rahonavis* and *U. comahuensis*. In other unenlagiines the ilium is not preserved. The presence of a well-developed supracetabular crest forming a “hood” over the femoral head is a feature recovered as plesiomorphic for Coelurosauria (Fig. 20). On the other hand, a reduced or absent supracetabular crest is convergently present in some tyrannosaurids, alvarezsaurids and some therizinosaurs and troodontids (Fig. 20). Absence of a supracetabular crest is the plesiomorphic condition for Pennaraptora, and the crest is absent in avialans, troodontids and in most dromaeosaurids, such in *Mahakala*, *Sinornithosaurus*, *Microraptor*, *Hesperonychus* (Xu, 2002; Hwang et al., 2002; Turner et al., 2007; Turner, Pol & Norell, 2011; Longrich & Currie, 2009) and the remaining Laurasian dromaeosaurids. The condition in unenlagiines thus appears to represent a reversal.

**Concave dorsal border of the postacetabular iliac blade in lateral view:** In *Rahonavis*, *Buitreraptor*, *U. comahuensis* and *U. paynemili*, the vertical iliac blade terminates short of the end of the brevis shelf, which imbues the posterodorsal edge of the ilium with a concave outline in lateral view. This character state is exclusively seen in unenlagiines (Fig. 20), whereas in most dromaeosaurids, including *Mahakala*, and the remaining coelurosaurs the dorsal border of the postacetabular blade is straight or convex. Recently, Agnolín & Novas (2013) considered this condition to be more widespread among parvians, interpreting it as present in *Tianyuraptor*, some specimens of *Archaeopteryx*, *Sapeornis* and *Confuciusornis* (Agnolín & Novas, 2013). However, direct observation of specimens of *Tianyuraptor*, *Sapeornis* and *Confuciusornis* (Peter J. Makovicky, 2013, personal observation) could not verify this claim, and neither did we find supporting evidence in the literature (Chiappe et al., 1999; Zhou & Zhang, 2003b; Zheng et al., 2010). Furthermore, this condition is not convincingly present in specimens of *Archaeopteryx* (Wellnhofer, 1974; Ostrom, 1976a; Peter J. Makovicky, 2012, personal observation on Berlin, Munich, and London specimens). However, we acknowledge that the morphology of the dorsal border of the postacetabular iliac blade is unknown in several microraptorines and other Laurasian dromaeosaurids, so future discoveries may affect the optimization of this trait.

**Preacetabular process of the ilium markedly longer than the postacetabular process:** This is a derived character state observed in *Buitreraptor*, *Rahonavis* and *Unenlagia*, the unenlagiine taxa with a preserved ilium, and is also shared with avialans (Fig. 20). The plesiomorphic condition for coelurosaurs is a preacetabular blade roughly as long as postacetabular process. Several dromaeosaurids present the plesiomorphic condition, such as *Mahakala*, *Microraptor*, *Adasaurus* and *Achillobator* (Perle, Norell & Clark, 1999; Hwang et al., 2002; Xu, 2002; Norell & Makovicky, 2004; Turner, Pol & Norell, 2011; Turner, Makovicky & Norell, 2012). However, the ilium is not sufficiently preserved in some basal microraptorines and *Shanag*, whereas *Tianyuraptor*, *Bambiraptor* and *Saurornitholestes* share an asymmetric illium with a longer preacetabular portion with unenlagiines (Burnham, 2004; Zheng et al., 2010; Turner, Makovicky & Norell, 2012). Moreover, *Xiaotingia* also has a long preacetabular blade, whereas in several troodontids this character cannot be scored based on the available information to date. Thus, the synapomorphic status of this character for Unenlagiinae may change with new discoveries and information.

**Presence of a ginglymoid distal articulation on metatarsal II:** In dromaeosaurids this metatarsal is characterized by a distal articular surface with two well-developed hemicondyles separated by a deep intercondylar groove, a morphology that restricts movement of the first phalanx of the digit II to a vertical plane. This feature is ubiquitous among dromaeosaurids and is recovered as a synapomorphy of the group (Fig. 20). However, some variation is observed within this clade, as in taxa like *Deinonychus* and *Velociraptor* (Ostrom, 1969; Norell & Makovicky, 1997, 1999), the ginglymoid articulation is more prominent, whereas in microraptorines the ginglymoid nature of the articulation is more modestly developed with smaller distal condyles and a shallower intercondylar groove (Hwang et al., 2002; Xu, 2002). Metatarsal II of *Buitreraptor*, *Rahonavis* and *Neuquenraptor* have distal hemicondyles separated by a distinct groove, but resemble the morphology observed in microraptorines in terms of how pronounced these features are. This difference in the development of the articulation led some authors to question the presence of a true ginglymus on the metatarsal II of unenlagiines (Agnolín & Novas, 2011). Nevertheless, the condition of unenlagiines clearly differs from that observed in troodontids, where metatarsal II lacks separation between hemicondyles as in *Sinovenator*, *Talos*, *Sinornithoides* and *Gobivenator* (Currie & Dong, 2001; Xu et al., 2002; Zanno et al., 2011; Tsuihiji et al., 2014). Moreover, in unenlagiines the intercondylar groove extends onto the anterior surface of the articulation, whereas among troodontids the anterior surface is smooth, as can be observed for example in *Talos* (Zanno et al., 2011). A similar morphology to that of troodontids is also present in most basal avialans (e.g., *Archaeopteryx* and *Confuciusornis*, Wellnhofer, 1974; Chiappe et al., 1999), and is the plesiomorphic condition for coelurosaurs (Fig. 20). While it is correct that the articular morphology observed in unenlagiines is not as markedly ginglymoid as in more derived dromaeosaurids, it differs unquestionably from the smooth distal articular surface of other coelurosaurs.

**Flexor heel on phalanx II-2 small and asymmetrically developed and morphology of pedal digit II:** In both dromaeosaurids and troodontids the proximoventral end of pedal phalanx II-2 is posteriorly extended into a flexor heel, which covers the distal and ventral surface of the phalanx II-1 when they are articulated. This process varies in size and development among dromaeosaurids. In most eudromaeosaurids the heel is large and reaches far posteriorly, and is symmetrically developed in dorsal/ventral view. This represents the derived condition among dromaeosaurids, relative to the shorter and more asymmetric flexor heel encountered in unenlagiine and microraptorine taxa. However, even among unenlagiines we observe some morphological variation in this process, as noted above. The transversely compressed and medially displaced proximoventral heel of *Buitreraptor* differs from the anatomy of later unenlagiines.

A derived pedal digit II, that allows hyper-extension of phalanx II-2, and bears an enlarged ungual phalanx that is significantly more recurved than the other pedal unguals, is a feature that differentiates dromaeosaurids and troodontids from other coelurosaurs (Fig. 20). This morphology is observed in most troodontids and dromaeosaurids, including *Mahakala* and unenlagiines. It has long been considered a hallmark synapomoprhy of Deinonychosauria, a result we did not recover here, instead recovering these traits as convergent between the two clades. Some previous studies (Agnolín & Novas, 2011) considered traits associated with a raptorial digit II as widespread in paravians, since it is purportedly also be observed in basal avialans. In fact other authors (Paul, 2002; Mayr et al., 2007) have studied the morphology of the pedal digit II of *Archaeopteryx* and suggested that it possessed the capacity for hyper-extension of phalanx II-2. According to these authors, phalanx II-1 of *Archaeopteryx* has a distal articulation which is markedly proximodorsally developed and would allow a remarkable extension of the phalanx II-2. Paul (2002) also refers to other possible evidence indicating that *Archaeopteryx* would have keep digit II extended while walking, without contact with the substrate, e.g., a strengthening of metatarsals III and IV which allowed the animal support its weigth only on the digits III and IV. Nevertheless, in dromaeosaurids and troodontids the distal articulation of the phalanx II-1 is clearly more dorsally extended than in *Archaeopteryx*, as can be observed in *Sinornithoides*, *Sinovenator*, *Dromaeosaurus*, *Deinonychus*, *Sinornithosaurus* and *Hesperonychus* (Colbert & Russell, 1969; Ostrom, 1969; Currie & Dong, 2001; Xu, 2002; Longrich & Currie, 2009). Among unenlagiines, phalanx II-1 is also very similar to the latter taxa. In dromeosaurids, including unenlagiines, and in troodontids, phalanx II-2 is highly modified, with a markedly dorsoventrally constricted shaft and an expanded distal articulation, especially proximoventrally. These traits are not observed in *Archaeopteryx* (Wellnhofer, 1974; Peter J. Makovicky, 2006, personal observation of the Berlin specimen, HMN MB. 1880/81) or other basal avialans, such as *Jeholornis* (Zhou & Zhang, 2002, 2003a). Other authors (Turner, Makovicky & Norell, 2012) agree that in *Archaeopteryx* phalanx II-1 has a distal articular surface that is not markedly proximodorsally developed and phalanx II-2 lacks both an expanded distal articulation and a proximoventral heel. Lastly, in dromaeosaurids and troodontids the ungual of digit II is comparatively larger with respect to the other pedal unguals and with a well-developed flexor tubercle. On the other hand, in *Archaeopteryx* and *Jeholornis* the ungual of the digit II is not significantly larger than those of digits III and IV (Wellnhofer, 1974; Paul, 2002; Zhou & Zhang, 2002; Senter et al., 2004; Mayr et al., 2007).

## Conclusion

*Buitreraptor* is the most complete Gondwanan dromaeosaurid found to date. Several postcranial characters support its assignment to Dromaeosauridae, including a pedal digit II with a second phalanx markedly modified for extreme hyper-extension and an ungual comparatively more strongly curved and larger than the ungual of digit III; parapophyses of the dorsal vertebrae projected on pedicels; a bicipital scar on the ulna developed as a slightly raised ridge; an ischium with a longitudinal ridge on the lateral surface; and a ginglymoid distal articular surface of the metatarsal II.

*Buitreraptor* has a number of postcranial characters that distinguish it from other unenlagiines and dromaeosaurids generally, which include anterior cervical centra extending beyond the posterior end of the neural arch; the dorsal rim of the ilium strongly laterally everted; posterior lateroventral ridges on the centra of the eighth and ninth cervical vertebrae terminating posteriorly in small tubercles; dorsal vertebrae with pneumatic foramina only in the first and second dorsal (reversal); a scapular blade transversely expanded at mid-length; and a pneumatic furcula with two pneumatic foramina flanking the hypocleideum.

The number and fusion of sacral vertebrae probably increased with ontogeny, since specimens representing younger individuals (MPCA 245) have five sacrals and the last vertebra unfused to the rest of the sacrum, whereas specimens representing older individuals exhibit a fused last sacral (MPCA 238) and six completely fused sacrals (MPCN-PV-598).

The ginglymoid distal articular surface of the metatarsal II of *Buitreraptor* is not as developed as in derived dromaeosaurids and is similar to that observed in other unenlagiines and microraptorines. However, this articular surface differs significantly from that of troodontids and other coelurosaurs, in which the condyles are less developed and the intercondylar groove is much less marked or absent. Thus, the ginglymus of metatarsal II in *Buitreraptor* and other unenlagiines may represent an intermediate condition between the plesiomorphic state and a more specialized morphology in derived dromaeosaurids.

The almost complete skeleton of *Buitreraptor* permits comprehensive characterization of a Gondwanan dromaeosaurid and merits a description and discussion of the features common to unenlagiines and those characters that link unenlagiines with other dromaeosaurids.

## Supplemental Information

10.7717/peerj.4558/supp-1 Supplemental Information 1 Strict consensus of the MPTs obtained from the phylogenetic analysis.

10.7717/peerj.4558/supp-2 Supplemental Information 2 Selected measurements of the postcranial bones of the holotype and referred specimens of *Buitreraptor gonzalezorum*.

10.7717/peerj.4558/supp-3 Supplemental Information 3 Character list used for phylogenetic analysis.

10.7717/peerj.4558/supp-4 Supplemental Information 4 Character matrix.

## Institutional Abbreviations

FMNH: Field Museum of Natural History, Chicago, US
HMN MB: Museum für Naturkunde, Berlin, Germany
IGM: Mongolian Institute of Geology, Ulaan Bataar, Mongolia
IVPP: Institute of Vertebrate Paleontology and Paleoanthropology, Beijing, China
MCZ: Museum of Comparative Zoology, Harvard University, Cambridge, US
MCF PVPH: Museo “Carmen Funes”, Plaza Huincul, Neuquén, Argentina.
MML: Museo Municipal de Lamarque, Río Negro, Argentina
MPCA: Museo Provincial “Carlos Ameghino,” Cipolletti, Río Negro, Argentina
MPCN: Museo Patagónico de Ciencias Naturales, General Roca, Río Negro, Argentina.
MUCPv: Museo de Geología y Paleontología de la Universidad Nacional del Comahue, Neuquén, Argentina.
UA: Université d’Antananarivo, Antananarivo, Madagascar.

## Additional Information and Declarations

### Data Availability

The following information was supplied regarding data availability: The raw data are provided in the Supplemental Dataset Files.
