# Supplementary material for: Postcranial skeletal anatomy of the holotype and referred specimens of Buitreraptor gonzalezorum Makovicky, Apesteguía and Agnolín 2005 (Theropoda, Dromaeosauridae), from the Late Cretaceous of Patagonia
Source: PeerJ. 2018 Mar 26;6:e4558. doi: 10.7717/peerj.4558 (PMC5875404; doi:10.7717/peerj.4558)
Supplement: Supplemental Information 3 [file peerj-06-4558-s003.docx]

**Supplemental File**

Characters added to the list of 853 phenotypic characters of Brusatte et al. (2014).

**Character 854:** **Length of the skull (Novas et al., 2009)**

0: less than the length of the femur

1: the same or 25% greater than the length of the femur

2: more than 25% the length of the femur

Modified: a new state 1 was added because some taxa, e.g., *Yanornis martini*, have a skull longer than the femur but less than 25% of the length of this bone.

**Character 855: Postantral wall of the maxilla posteriorly extended (Novas et al., 2009)**

0: absent

1: present

**Character 856:** **Striations on the crowns of the teeth (Gianechini et al., 2011)**

0: absent

1: present

Striations on the crowns are considered as two or more longitudinal furrows on the labial and/or lingual surface. Isolated furrows, as those observed in *Sinornithosaurus millenii*, are not considered as striations.

Characters from Hu et al. (2009)

**Character 857:** **Dorsal centra**

0: ≥ 1.2×taller than long

1: height ≤ length

**Character 858: Length of manual digit II (including metacarpal)**

0: less than 1.25×femoral length

1: ≥ 1.25×femoral length

**Character 859: Manual phalanx I-1 (Pérez-Moreno et al., 1994)**

0: longer than metacarpal II

1: shorter than metacarpal II

**Character 860: Strong kink of pubis at midshaft**

0: absent

1: present, displacing distal half of pubis caudally

**Character 861:** **In adult, femur length**

0: longer than tibia

1: shorter than tibia

**Character 862:** **Metatarsus length**

0: less than half length of femur

1: more than half femoral length

**Character 863: Length of pedal phalanx II-2**

0: between 0.6×and 1× length of phalanx II-1

1: ≤0.6 × length of phalanx II-1

2: ≥1× length of phalanx II-1

**Character 864:** **Manual phalanx II-1**

0: shorter than I-1

1: longer than I-1

**Character 865:** **Pneumatopores in anterior caudal vertebrae**

0: absent

1: present

**Character 866:** **Length of manual phalanx III-2**

0: subequal to length of phalanx III-1

1: significantly shorter than phalanx III-1

2: significantly longer than phalanx III-1

**Character 867:** **Location of the anteroventral process of ilium**

0: anteriorly located, close to the anterior end of the bone

1: posteriorly located, significantly away from the anterior end

**Character 868: Location of the postorbital process of the jugal**

0: located significantly anterior to the posterior end of the jugal

1: close to the posterior end, and consequently the quadratojugal process is very short

Characters from Gianechini et al. (2017)

**Character 869:** **Inclination of the ventral process of the lacrimal**

0: ventrally directed so the process is vertical

1: anteroventrally inclined

**Character 870:** **Inclination of the dorsal portion of the ilium above or slightly posterior to the acetabulum**

0: in the same plane than the remain of the iliac blade

1: strongly laterally curved, so the lateral surface of the iliac blade can be observed in ventral view

**Character 871:** **Extension of the supracetabular crest of the ilium**

0: extended only along the dorsal border of the acetabulum

1: anteriorly extended along the lateral surface of the pubic peduncle, reaching or almost reaching the ventral end of the peduncle

**Character 872:** **Angle between the posterior border of the pubic shaft and the dorsal border of the pubic boot**

0: less than 90°

1: greater than 90°

**Character 873:** **Dorsal surface of the acetabulum**

0: perpendicular with the lateral surface of the iliac blade, so the acetabulum is totally open

1: dorsal surface of the acetabulum ventrally and medially inclined, so the acetabulum tends to be obliterated

**Character 874:** **Anteroposterior extension of the pubic peduncle**

0: less than the anteroposterior extension of the acetabulum

1: between 1 and 1.5 the anteroposterior extension of the acetabulum

2: > 1.5 the anteroposterior extension of the acetabulum

**Character 875: Foramen in the ventral part of the splenial (mylohyoid foramen) (character from Rauhut, 2003)**

0: absent

1: completely enclosed in the splenial

2: opened anteroventrally

**Character 876: Middle of maxillary tooth row, spacing between teeth**

0: narrow, teeth separated by less than one crown width

1: wide, adjacent teeth separated by a gap corresponding to one crown width or more.

New characters

**Character 877: Pennaceous feathers on the body**

0: absent

1: present

**Character 878: Elongated pennaceous feathers on forelimbs**

0: absent

1: present and symmetric

2: present and asymmetric

**Character 879: Alula**

0: absent

1: present

**Character 880: Elongated pennaceous feathers on the tibia**

0: absent

1: present

**Character 881**: **Elongated pennaceous feathers on the foot (Metatarsus and/or and pedal phalanges)**

0: absent

1: present

**Character 882: Elongated pennaceous feathers on the tail**

0: absent

1: present

**Character 883: A pair of extremely elongated pennaceous feathers (e.g. PRPFs)**

0: absent

1: present

**Character 884: Egg geometry**

0: symmetric

1: asymmetric

2: asymmetric with modern avian outline

**Phylogenetic analysis performed from the original character ordering scheme proposed by Brusatte et al. (2014)**

A heuristic search for the most parsimonious topologies was conducted by performing 1000 replicates of Wagner trees (using RAS) followed by TBR branch swapping (holding 10 trees per replicate). Zero length branches were collapsed during the analysis. The search resulted in 1670 most parsimonious trees (MPTs) of 3676 steps, found 167 times out of the 1000 replications. A subsequent round of TBR branch swapping on these 1670 trees finally found more than 999,999 MPTs. The strict consensus showed a massive polytomy at the base of Maniraptoriformes (Supplemental Figure 1). After the pruning of *Kinnareemimus*, *Pyroraptor* and *Pamparaptor* this polytomy was partially resolved, although a polytomy prevailed at the base of Paraves, in which the microraptorines and the unenlagiines are outside Dromaeosauridae (Supplemental Figure 2).


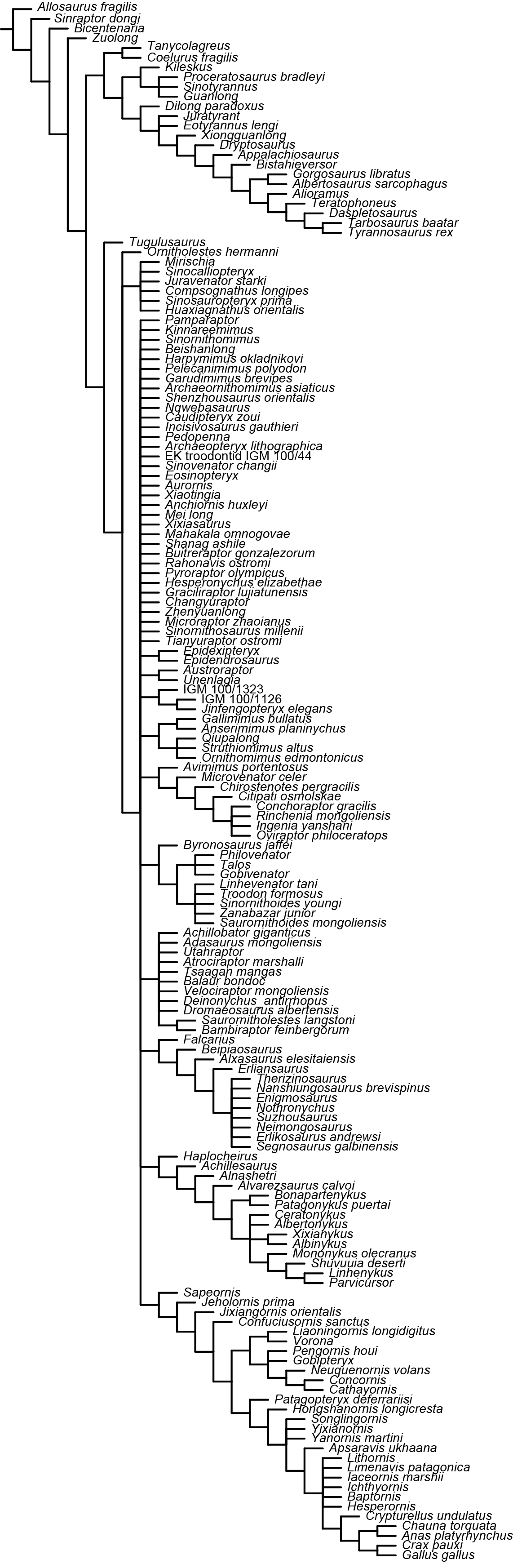


Supplemental figure 1: Strict consensus tree obtained from the phylogenetic analysis.


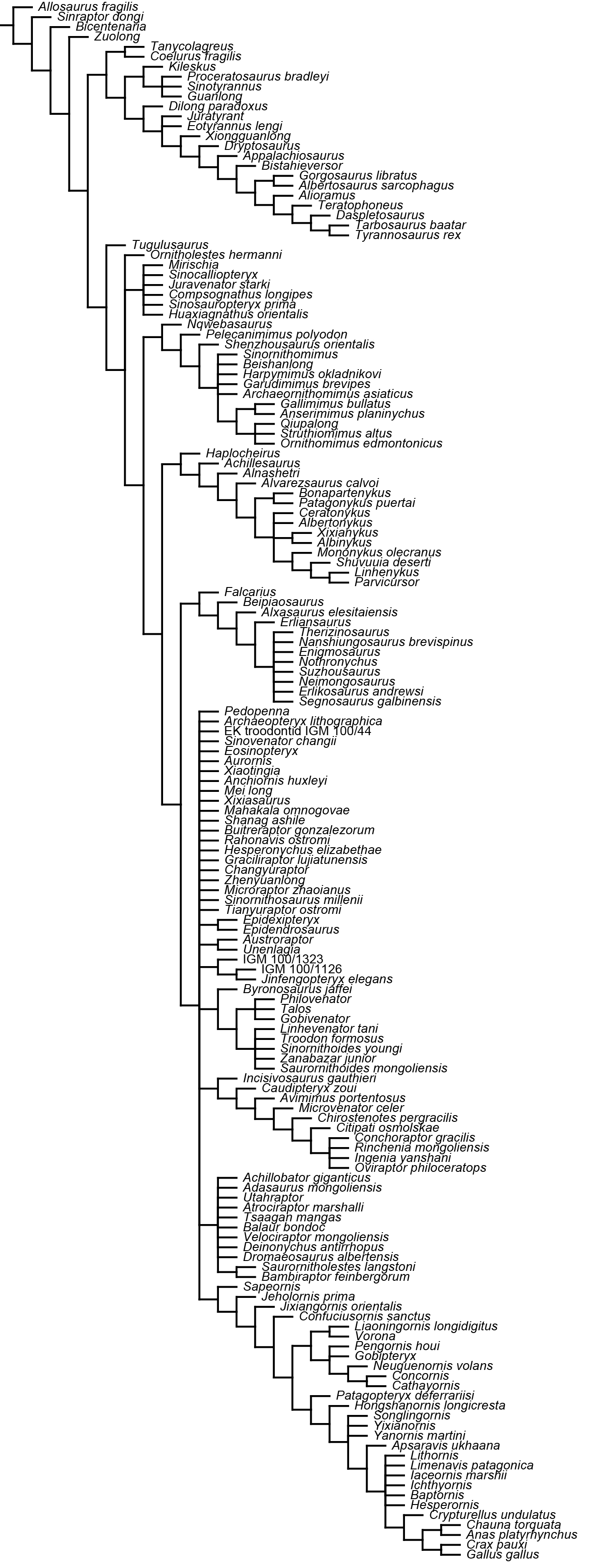


Supplemental figure 2: reduced consensus tree obtained from the phylogenetic analysis, after the pruning of *Kinnareemimus*, *Pamparaptor* and *Pyroraptor*.

**Supplemental references**

Brusatte SL, Lloyd GT, Wang SC, Norell MA. 2014. Gradual assembly of avian body plan culminated in rapid rates of evolution across the dinosaur-bird transition. *Current Biology* 24:2386–2392.

Gianechini FA, Makovicky PJ, Apesteguía S. 2011. The teeth of the unenlagiine theropod *Buitreraptor* from the Cretaceous of Patagonia, Argentina, and the unusual dentition of the Gondwanan dromaeosaurids. *Acta Palaeontologica Polonica* 56:279‒290.

Gianechini FA, Makovicky PJ, Apesteguía S. 2017. The cranial osteology of *Buitreraptor gonzalezorum* Makovicky, Apesteguía, and Agnolín, 2005 (Theropoda, Dromaeosauridae), from the Late Cretaceous of Patagonia, Argentina. *Journal of Vertebrate Paleontology* 37:e1255639. DOI: 10.1080/02724634.2017.1255639

Hu D, Hou L, Zhang L, Xu X. 2009. A pre-*Archaeopteryx* troodontid theropod from China with long feathers on the metatarsus. *Nature* 461:640‒643.

Novas FE, Pol D, Canale JI, Porfiri JD, Calvo JO. 2009. A bizarre Cretaceous theropod dinosaur from Patagonia and the evolution of Gondwanan dromaeosaurids. *Proceedings of the Royal Society B* 276:1101‒1107.

Pérez-Moreno BP, Sanz JL, Buscalioni AD, Moratalla JJ, Ortega F, Rasskin-Gutman D. 1994. A unique multitoothed ornithomimosaur from the Lower Cretaceous of Spain. *Nature* 370:363–367.

Rauhut OWM. 2003. The interrelationships and evolution of basal theropod dinosaurs. *The Palaeontological Association, Special Papers in Paleontology* 69:1‒214.
